# Supplementary figures and images for: Ethylenediurea (EDU) inhibits OsORAP1 expression in rice (Oryza sativa L:): Varietal differences in ozone protection efficacy
Source: PLoS One. 2025 Jul 2;20(7):e0327162. doi: 10.1371/journal.pone.0327162 (PMC12220992; doi:10.1371/journal.pone.0327162)

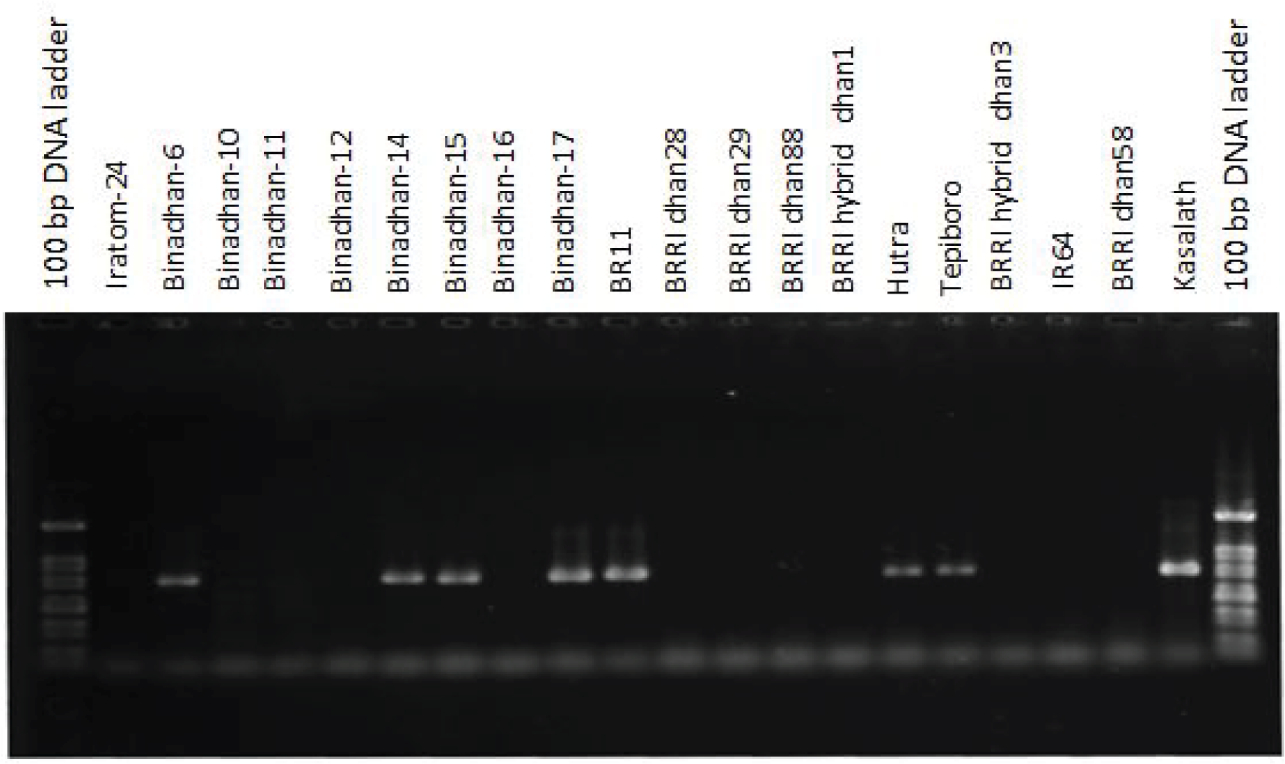

Supplement: S1 Fig — “S1_fig.tif”. (TIF) [file pone.0327162.s001.tif]

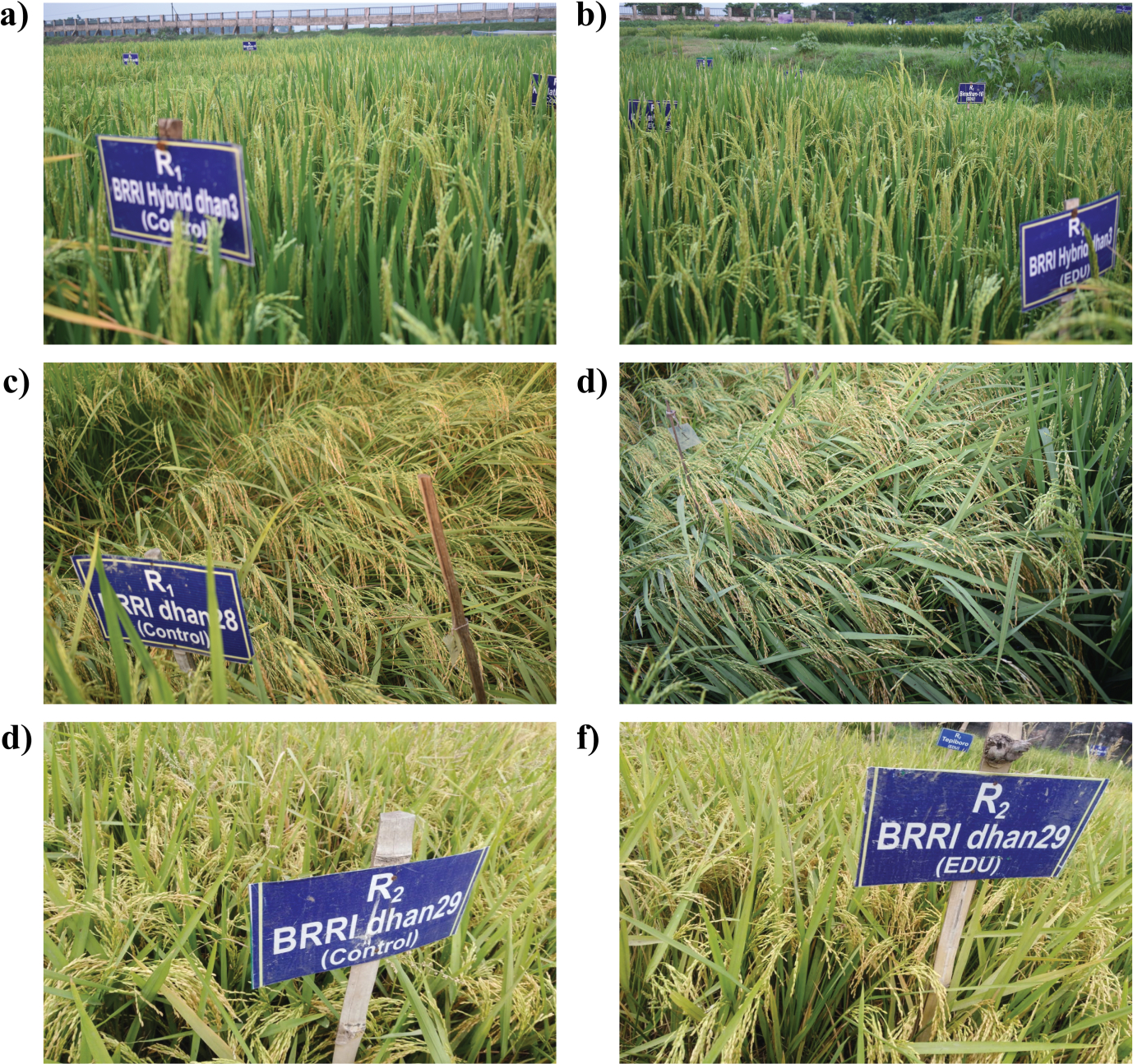

Supplement: S2 Fig — [Shown are images of six rice variety with two treatments: (a) BRRI hybrid dhan3 without EDU (Control), (b) BRRI hybrid dhan3 with EDU, (c) BRRI dhan28 without EDU (Control), (d) BRRI dhan28 with EDU, (e) BRRI dhan29 without EDU (Control), and (f) BRRI dhan29 with EDU. Plants were exposed to ambient ozone stress, and EDU (ethylenediurea) was applied to assess its protective effects against ozone stress]. “S2_fig.tif”. (TIF) [file pone.0327162.s003.tif]
