# Supplementary material for: Ethylenediurea (EDU) inhibits OsORAP1 expression in rice (Oryza sativa L:): Varietal differences in ozone protection efficacy
Source: PLoS One. 2025 Jul 2;20(7):e0327162. doi: 10.1371/journal.pone.0327162 (PMC12220992; doi:10.1371/journal.pone.0327162)
Supplement: S1 Raw Image Fig — “S1_fig.pdf”. (PDF) [file pone.0327162.s002.pdf]

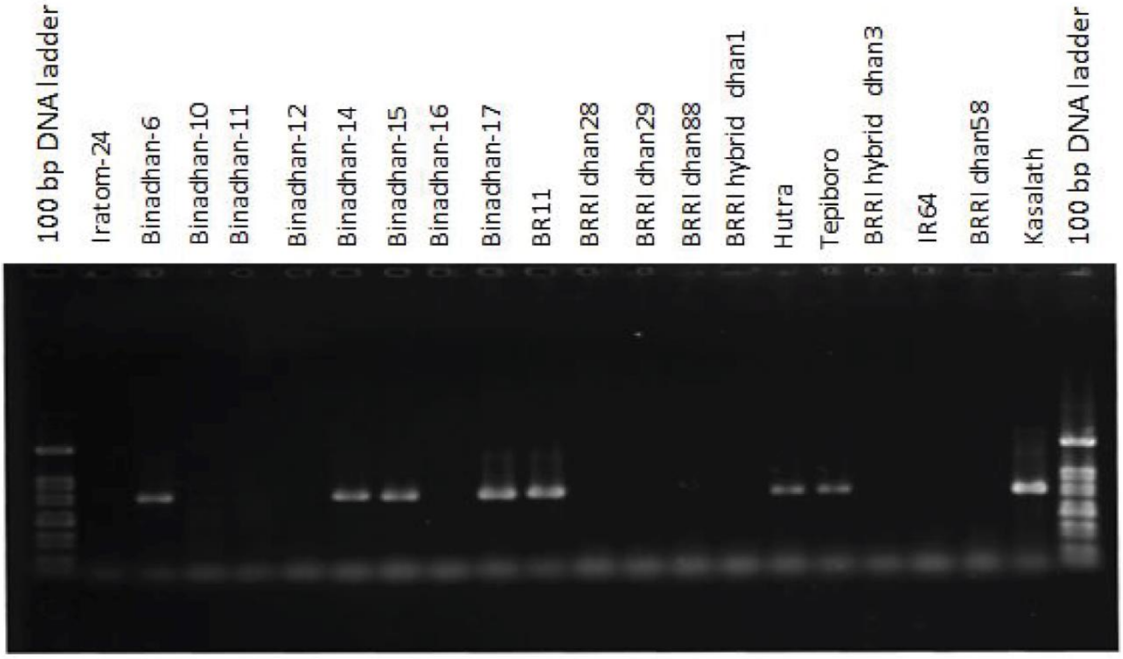

Manuscript main figure. The DNA profile of 20 rice genotypes using primer KAS_1_2, which showing the band on Kasalath-type (specific) genotypes

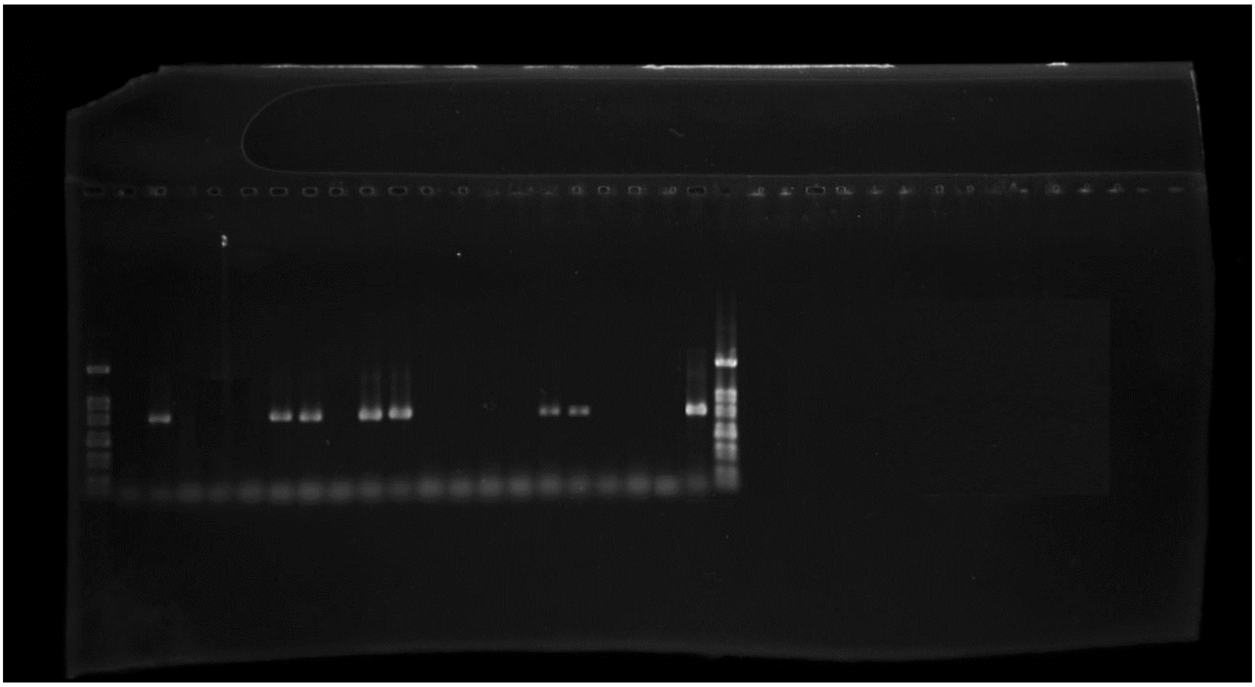

Supplemental figures. The DNA profile of 20 rice genotypes using primer KAS_1_2, which showing the band on Kasalath-type (specific) genotypes
