# Supplementary material for: Ethylenediurea (EDU) inhibits OsORAP1 expression in rice (Oryza sativa L:): Varietal differences in ozone protection efficacy
Source: PLoS One. 2025 Jul 2;20(7):e0327162. doi: 10.1371/journal.pone.0327162 (PMC12220992; doi:10.1371/journal.pone.0327162)
Supplement: Attachment — Submitted filename: PONE-D-25-00588_R1.pdf-rev1.pdf [file pone.0327162.s009.pdf]

PLOS ONE

Expression of OsORAP1 allele in rice (Oryza sativa L.) at ambient ozone stress in relation to ethylenediurea (EDU) application

--Manuscript Draft--

**Manuscript Number:**	PONE-D-25-00588R1
**Article Type:**	Research Article
**Full Title:**	Expression of OsORAP1 allele in rice (Oryza sativa L.) at ambient ozone stress in relation to ethylenediurea (EDU) application
**Short Title:**	Expression of OsORAP1 allele in rice at ambient ozone stress under EDU
**Corresponding Author:**	Md Ashrafuzzaman, PhD<br>Shahjalal University of Science and Technology<br>Sylhet, BANGLADESH
**Keywords:**	Ozone, Stress, Ethylenediurea (EDU), OsORAP1, Rice, Food security, Global change
**Abstract:**	Rising tropospheric ozone concentrations pose a significant threat to crop production in many Asian countries like Bangladesh, necessitating the development of ozone-tolerant rice (Oryza sativa L.) varieties. This study investigates the response of OsORAP1 allele in different rice varieties, particularly Kasalath-type and Nipponbare-type, to Ethylenediurea (EDU) treatment under ambient ozone stress. The experiment, conducted during the 2022 rainfed season, involved growing of 20 rice varieties under a split-plot design with and without EDU treatment, followed by genomic DNA collection at the vegetative stage to differentiate Kasalath-types and Nipponbare-types, and RNA extraction from 10 selected varieties at the flowering stage for gene expression analysis. The research revealed distinct genetic responses between the two types, with Nipponbare-type varieties showing significant increases in OsORAP1 expression and more leaf bronzing score (LBS) following EDU treatment. In contrast, Kasalath-type varieties exhibited less pronounced responses due to their inherent tolerance to ozone stress. The effects of EDU on with Kasalath-type varieties responding notably in parameters such as ineffective tillers and straw yield, while Nipponbare-types showed significant changes in unfilled grains and grain yield. Additionally, differences were observed in how EDU treatment influenced reflectance indices parameters, indicating variability in how these varieties mitigate ozone stress. The study underscores the complex interaction between genetic factors, environmental conditions, and treatment in shaping the response of rice varieties to EDU, and suggests future research to further explore the genetic mechanisms, particularly the role of OsORAP1, that contribute to these differential responses.
**Order of Authors:**	Rigyan Gupta, MSc
	Shamsul H. Prodhan, PhD
	Md. Nazmul Hasan, PhD
	Shamsun Nahar Begum, PhD
	Mohammad Hasanuzzaman Rani, PhD
	Mirza Mofazzal Islam, PhD
	Md Ashrafuzzaman, PhD
	Editorial Team
**Response to Reviewers:**	Academic editor and Reviewer response<br><br>Academic editor<br>Comments 1: Please ensure that your manuscript meets PLOS ONE's style requirements, including those for file naming. The PLOS ONE style templates can be found at<br><br>https://journals.plos.org/plosone/s/file?id=wjVg/PLOSOne_formatting_sample_main_body.pdf and

Powered by Editorial Manager® and ProduXion Manager® from Aries Systems Corporation

https://journals.plos.org/plosone/s/file?id=ba62/PLOSOne_formatting_sample_title_authors_affiliations.pdf

Author response: Thank you for your valuable comments. We have revised the manuscript to ensure it fully complies with PLOS ONE's style requirements, including formatting and file naming, as per the provided guidelines.

Comments 2: PLOS ONE now requires that authors provide the original uncropped and unadjusted images underlying all blot or gel results reported in a submission's figures or Supporting Information files. This policy and the journal's other requirements for blot/gel reporting and figure preparation are described in detail at https://journals.plos.org/plosone/s/figures#loc-blot-and-gel-reporting-requirements and https://journals.plos.org/plosone/s/figures#loc-preparing-figures-from-image-files. When you submit your revised manuscript, please ensure that your figures adhere fully to these guidelines and provide the original underlying images for all blot or gel data reported in your submission. See the following link for instructions on providing the original image data: https://journals.plos.org/plosone/s/figures#loc-original-images-for-blots-and-gels.

In your cover letter, please note whether your blot/gel image data are in Supporting Information or posted at a public data repository, provide the repository URL if relevant, and provide specific details as to which raw blot/gel images, if any, are not available. Email us at plosone@plos.org if you have any questions.

Author response: Thank you for your valuable comments. We have revised our submission to fully comply with PLOS ONE's guidelines. The original uncropped and unadjusted images underlying all blot or gel results have been provided as per the journal's requirements. In the cover letter we have mention the blot/gel image data are in Supporting Information.

Comments 3: Please include captions for your Supporting Information files at the end of your manuscript, and update any in-text citations to match accordingly. Please see our Supporting Information guidelines for more information: http://journals.plos.org/plosone/s/supporting-information.

Author response: Thank you for your valuable comments. We have revised our submission to fully comply with PLOS ONE's guidelines. We have included captions for the Supporting Information files at the end of the manuscript and updated all in-text citations accordingly.

Comments 4: We notice that your supplementary figures are uploaded with the file type 'Other'. Please amend the file type to 'Supporting Information'. Please ensure that each Supporting Information file has a legend listed in the manuscript after the references list.

Author response: Thank you for your valuable comments. We have revised our submission to fully comply with PLOS ONE's guidelines. We have updated the file type of the supplementary figures to 'Supporting Information' and ensured that each Supporting Information file has a corresponding legend listed in the manuscript after the references list.

Reviewer #1

Comments 1: The study included 20 rice varieties, but the criteria for their selection were not clearly defined. For example, whether these varieties are representative of all major cultivar types and whether they contain sufficient genetic diversity are not addressed. Without a comprehensive and representative sample, the generalizability of the study results may be compromised.

Author response: Thank you for your valuable comment. We selected 20 diverse indica rice varieties to ensure genetic variation and broad representation. These included popular mega varieties of Bangladesh where nine inbred varieties developed by the Bangladesh Institute of Nuclear Agriculture (BINA), seven varieties from the Bangladesh Rice Research Institute (BRRI) (including two hybrid and five inbred), three locally cultivated inbred varieties from Bangladesh (including Kasalath, an ozone-tolerant variety), and one variety from the International Rice Research Institute (IRRI). Details are now provided in Table 1 and elaborated in Section 2.1 of the manuscript.

Comments 2: Although the experiment included four replications, whether the statistical analysis adequately accounted for random error and replication should be detailed.

Author response: Thank you for your comment. We used four replications to ensure data reliability. Our statistical analysis accounted for variability, and no significant random errors or differences among replications were observed. Section 2.5 of the

Powered by Editorial Manager® and ProduXion Manager® from Aries Systems Corporation

manuscript now details our approach to addressing replication in the analysis
Comments 3: EDU is applied weekly by foliar spraying, but specific times and conditions of application (e.g., light, temperature) are not detailed. These factors may greatly influence the effectiveness of EDU.
Author response: Thank you for this insight. We have now included specific details regarding EDU application conditions, such as light and temperature, in Section 2.4 of the manuscript. We have also refined the research objectives for greater coherence and precision.
Comments 4: The experimental procedure is described in detail in the “Materials and Methods” section, but the order in which the results are presented in the “Results” section does not correspond to the experimental design, which may confuse the reader.
Author response: Thank you for your suggestion. We have revised the "Materials and Methods" section for greater clarity and adjusted the "Results" section to enhance logical flow and coherence while maintaining our focus on the relationship between OsORAP1 expression and yield-related data.
Comments 5: Although the study mentions using R software for statistical analysis, the specific methods (e.g., whether multiple comparison corrections were applied) are not detailed. Moreover, for significant results (e.g., p-values), the biological implications are not further explained.
Author response: Thank you for your valuable comment. Section 2.5 now provides details on our statistical methods, including multiple comparison corrections. We have also expanded the discussion on the biological implications of significant results, particularly regarding EDU effects.
Comments 6: The study does not deeply explore the potential reasons for the differential responses to EDU among varieties. For example, why Kasalath-type varieties show less significant responses to EDU compared to Nipponbare-type varieties is not discussed. Are these differences related to genetic background or environmental adaptability?
Author response: Thank you for your insightful comment. We have examined differential responses by analyzing OsORAP1 expression. Previous studies (Ashrafuzzaman et al., 2017, 2018) suggest that tolerant genotypes exhibit minimal response to EDU compared to sensitive ones. This variation is likely due to genetic background, which may influence EDU’s effectiveness in mitigating ozone stress.
Comments 7: The figures in the study could be further optimized. For example, some data labels in the figures are unclear, or sufficient legends are not provided.
Author response: Thank you for your observation. We have revised the figures to improve clarity, including better data labeling and more comprehensive legends for enhanced readability.
Comments 8: When presenting experimental results, some data lack complete statistical information. For example, for certain parameters (e.g., chlorophyll indices), only the mean values are provided without standard deviations or confidence intervals.
Author response: We have reviewed and updated statistical details in Section 2.2, incorporating standard deviations or confidence intervals where necessary. Figure labels have also been improved for better clarity.
Reviewer #2
Comments 1: In line 88-90, authors have mentioned “absence of OsORAP1…”. Does this mean OsRAP1 gene does not exist in tolerant genotype?
Author response: Thank you for your comment. The phrase "absence of OsORAP1" refers to its low or no expression in tolerant genotypes, not the gene’s physical absence. We have revised the text for clarity.
Comments 2: In line 95 – 97, higher induction of OsORAP1 should lead to susceptibility not tolerance.
Author response: Thank you for pointing this out. We have revised the manuscript to accurately convey this concept.
Comments 3: Please provide methodology used for deriving various indices (NDVI, PRI, SIPI etc.)
Author response: Thank you for your valuable comment. We have provided the methodology for deriving various indices (NDVI, PRI, SIPI, etc.) in the "Materials and Methods" section, specifically in Section 2.2 ("Growing of Varieties and Design of the Experiments").
Comments 4: In line 168-170, authors have mentioned “Twenty genotypes (genotypes with Kasalath-type OsRAP1 allele)”. However, all the 20 genotypes were not kasalath-type. This has to be rewritten.

Powered by Editorial Manager® and ProduXion Manager® from Aries Systems Corporation

Author response: We apologize for the oversight. The sentence has been revised to: "Among the twenty genotypes, selection was based on the presence or absence of a PCR band for further analyses."
Comments 5: In line no 170-173, results of PCR have been described, which may be shifted to results section.
Author response: Thank you for your valuable comment. Since our study primarily focuses on the expression of OsORAP1, we included the PCR band results in the "Materials and Methods" section to provide necessary context for our experimental approach. However, we have reconsidered the placement to ensure clarity and coherence in the manuscript.
Comments 6: Was the primer KAS_1_2 designed in this study or was it sources from literature. If it is designed by the authors, the methodology and the basis has to be elaborated.
Author response: The primer KAS_1_2 was sourced from Ashrafuzzaman et al., 2020. This clarification has been added to the manuscript.
Comments 7: The KAS_1_2 primer validation should be undertaken between Kasalath and Nipponbare. This is essential step for establishing genotypes as Kasalath-type and Nipponbare-type. However, Nipponbare is missing in the study.
Author response: Thank you for your suggestion. Prior studies (Ashrafuzzaman et al., 2020) confirm KAS_1_2 amplifies only in Kasalath-type genotypes, while Nipponbare-type primers produce bands exclusively in Nipponbare-type genotypes. We have clarified this in the manuscript.
Comments 8: Methodology used for scoring leaf bronzing, yield traits and reflectance indices should be included in the materials and methods section.
Author response: Thank you for your valuable comment. We appreciate your suggestion and have now included the methodology for scoring leaf bronzing, yield traits, and reflectance indices in the "Materials and Methods" section of the manuscript.
Comments 9: In line 118 of materials and methods section, it has been mentioned that 20 genotypes were evaluated in field. However, ANOVA for 10 selected genotypes has been presented.
Author response: Thank you for your valuable comment. Out of the 20 genotypes evaluated, we selected 10 for further analysis (5 Kasalath-type and 5 Nipponbare-type). Therefore, the ANOVA was conducted based on these 10 selected genotypes, which is why the results are presented accordingly.
Comments 10: Please shift the content of line no 248-249 to respective discussion section.
Author response: Thank you for your valuable comment. We have moved the content from lines 248-249 to the respective discussion section as suggested.
Comments 11: In fig 3, Kasalath is negatively responding to EDU treatment for leaf bronzing. However, authors have mentioned that Kasalath is not responsive to EDU treatment. This needs to be clarified.
Author response: Thank you for your valuable comment. We acknowledge the discrepancy in Figure 3, where Kasalath appears to negatively respond to EDU treatment for leaf bronzing. This response may be due to environmental factors or other variables influencing the outcome. We have clarified this in the manuscript to address the observed effect.
Comments 12: In Nipponbare-type varieties, BRRI hybrid dhan3 and BRRI dhan29 exhibited significant increase in grain yield/ plant and straw yield/ plant in response to EDU treatment, while other varieties did not show significant changes in either parameter. This needs to be discussed.
Author response: Thank you for your valuable comment. In Nipponbare-type varieties, BRRI hybrid dhan3 and BRRI dhan29 showed a significant increase in grain yield per plant and straw yield per plant in response to EDU treatment, while other varieties did not exhibit statistically significant changes. However, other Nipponbare-type varieties still demonstrated higher grain and straw yields, which may be influenced by genetic or environmental factors. We have included this discussion in the manuscript to provide further clarification.
Comments 13: Among the Kasalath-type allele carrying varieties, EDU treatment in one of the variety (BR11) has increased the grain and straw yield, while none of the remaining varieties have responded. This needs to be discussed.
Author response: Thank you for your valuable comment. Among the Kasalath-type allele-carrying varieties, BR11 exhibited an increase in grain and straw yield following EDU treatment, whereas the other varieties did not respond. This variation may be attributed to genetic or environmental factors. We have already addressed this point in

	the manuscript's discussion section.<br>Comments 14: Association between the reflectance indices with yield traits should be estimated.<br>Author response: Thank you for your valuable comment. We have analyzed the association between reflectance indices and yield traits for both Kasalath-type and Nipponbare-type varieties, with and without EDU treatment, and also have incorporated this information into the manuscript.<br>Comments 15: Line No. 327-329, authors have mentioned that "EDU treatment and its interaction with varieties did not yield significant responses across any of the studied reflectance indices parameters. Nonetheless, significant differences were observed among the varieties themselves for all parameters, suggesting distinct genetic responses irrespective of the EDU treatment". This indicates that there exist inherent differences among the genotypes for the tested traits. This does not imply the variation in genotypes is due to EDU treatment or due to their interaction.<br>Author response: Thank you for your valuable comment. We acknowledge the clarification. Our statement aimed to highlight that while EDU treatment and its interaction with Kasalath-type and Nipponbare-type varieties where it did not show significant effects on reflectance indices, the inherent genetic differences among the varieties were evident. We agree that this variation is independent of EDU treatment.<br>Comments 16: In Table 3, among Kasalath-type genotypes, Binadhan-17, Hutra and Kasalath the yield and related parameters have reduced upon EDU treatment over control. Similar situation exists for Nipponbare-type genotypes. This is contradictory. Needs thorough discussion.<br>Author response: Thank you for your valuable comment. In Table 3, the reduction in yield and related parameters upon EDU treatment in Kasalath-type genotypes (Binadhan-17, Hutra, and Kasalath), as well as in some Nipponbare-type genotypes, may indicate that EDU has little to no protective effect on ozone-tolerant genotypes. Additionally, the observed variations could be influenced by environmental conditions or other genetic factors. We further elaborated on this in the discussion section to provide a clearer interpretation.<br>Comments 17: The OsORAP1 gene expression level is reduced by EDU treatment. However, establishing its association with the yield and other related parameters is essential.<br>Author response: Thank you for your valuable comment. Throughout our study, we have aimed to establish the association between OsORAP1 gene expression, yield, and other related parameters. We have further clarified this relationship in the discussion section to enhance the interpretation of our findings.<br>Comments 18: The treatment × variety interaction is non-significant for majority of the parameters under study. This indicates that the expression levels of OsRAP1 have no association with any of the tested parameters.<br>Author response: Thank you for your valuable comment. While the treatment × variety interaction is non-significant for some parameters, it is significant for key parameters such as OsORAP1 expression and grain yield per plant in both Kasalath-type and Nipponbare-type varieties, which form the foundation of our study. We have already ensured these in the manuscript.<br>Comments 19: In line 335-337, authors have mentioned that "These findings suggest that EDU application can effectively mitigate ambient ozone stress in Nipponbare-type varieties, with different reflectance indices indicating its positive effects on leaf greenness, and photosynthesis content". The statement is contradicting as the variation for reflectance indices under treatment and interaction component is non-significant.<br>Author response: We appreciate the reviewer's critical observation. We acknowledge that the statement in lines 335–337 may not have been clearly articulated. While the interaction effect and treatment variation for some reflectance indices were non-significant, the overall trends observed in leaf greenness and photosynthetic parameters indicate a positive influence of EDU application. To clarify this, we have revised the statement as follows:<br>"These findings suggest that EDU application can provide some degree of protection against ambient ozone stress in Nipponbare-type varieties. Although the variation for certain reflectance indices under treatment and interaction effects was not statistically significant, trends in leaf greenness and photosynthesis-related parameters indicate a positive influence of EDU."<br>This revision ensures consistency with the statistical results while maintaining the interpretation of EDU's protective effects. Thank you for pointing this out.

	Comments 20: Although, the ANOVA indicates no significant variation for treatment and interaction components for most of the parameters tested. However, authors indicate that there exists influence of treatment and its interaction with genotype although the text. Which is contradictory.<br>Author response: Thank you for your valuable comment. We acknowledge that ANOVA indicates no significant variation for treatment and interaction components for most of the parameters tested. However, within the varieties, while some parameters showed non-significant effects, key parameters such as OsORAP1 expression and grain yield per plant exhibited significant differences in both Kasalath-type and Nipponbare-type varieties. These significant findings form the foundation of our study and support our conclusions regarding the impact of EDU treatment under ozone stress. Thank you for your insightful feedback.<br>Comments 21: Line no 478 and 479 – Authors have mentioned that Nipponbare-type varieties have increased symptom formation and expression under EDU treatment. This contradicts the hypothesis of the paper.<br>Author response: Thank you for your valuable comment. We acknowledge that the statement in lines 478–479 may appear contradictory to our hypothesis. However, our findings indicate that Nipponbare-type varieties displayed significant increases in OsORAP1 expression and visible symptom formation (leaf bronzing score) in response to EDU treatment, while Kasalath-type varieties showed less significant responses due to their inherent tolerance to ozone stress. This suggests that EDU's protective effects may vary depending on the genetic background of the variety. To avoid confusion, we have revised the text for clarity and ensured that our interpretation aligns with the study's hypothesis. We appreciate your feedback in helping us improve the manuscript.
**Additional Information:**	
**Question**	**Response**
**Financial Disclosure**<br><br>Enter a financial disclosure statement that describes the sources of funding for the work included in this submission. Review the submission guidelines for detailed requirements. View published research articles from *PLOS ONE* for specific examples.<br><br>This statement is required for submission and **will appear in the published article** if the submission is accepted. Please make sure it is accurate.<br><br>**Funded studies**<br>Enter a statement with the following details:<br>• Initials of the authors who received each award<br>• Grant numbers awarded to each author<br>• The full name of each funder<br>• URL of each funder website<br>• Did the sponsors or funders play any role in the study design, data collection and analysis, decision to publish, or preparation of the manuscript?	The author(s) received no specific funding for this work.

Did you receive funding for this work?	
**Competing Interests**<br><br>Use the instructions below to enter a competing interest statement for this submission. On behalf of all authors, disclose any competing interests that could be perceived to bias this work—acknowledging all financial support and any other relevant financial or non-financial competing interests.<br><br>This statement is required for submission and **will appear in the published article** if the submission is accepted. Please make sure it is accurate and that any funding sources listed in your Funding Information later in the submission form are also declared in your Financial Disclosure statement.<br><br>View published research articles from *PLOS ONE* for specific examples.<br><br>**NO authors have competing interests**<br><br>Enter: *The authors have declared that no competing interests exist.*<br><br>**Authors with competing interests**<br><br>Enter competing interest details beginning with this statement:<br><br>*I have read the journal's policy and the authors of this manuscript have the following competing interests: [insert competing interests here]*<br><br>* typeset	The authors have declared that no competing interests exist.
**Ethics Statement**<br><br>Enter an ethics statement for this submission. This statement is required if the study involved:	N/A

• Human participants<br>• Human specimens or tissue<br>• Vertebrate animals or cephalopods<br>• Vertebrate embryos or tissues<br>• Field research<br><br>Write "N/A" if the submission does not require an ethics statement.<br><br>General guidance is provided below. Consult the submission guidelines for detailed instructions. **Make sure that all information entered here is included in the Methods section of the manuscript.**	

Powered by Editorial Manager® and ProduXion Manager® from Aries Systems Corporation

**Format for specific study types**<br><br>**Human Subject Research (involving human participants and/or tissue)**<br>• Give the name of the institutional review board or ethics committee that approved the study<br>• Include the approval number and/or a statement indicating approval of this research<br>• Indicate the form of consent obtained (written/oral) or the reason that consent was not obtained (e.g. the data were analyzed anonymously)<br><br>**Animal Research (involving vertebrate animals, embryos or tissues)**<br>• Provide the name of the Institutional Animal Care and Use Committee (IACUC) or other relevant ethics board that reviewed the study protocol, and indicate whether they approved this research or granted a formal waiver of ethical approval<br>• Include an approval number if one was obtained<br>• If the study involved *non-human primates*, add *additional details* about animal welfare and steps taken to ameliorate suffering<br>• If anesthesia, euthanasia, or any kind of animal sacrifice is part of the study, include briefly which substances and/or methods were applied<br><br>**Field Research**<br><br>Include the following details if this study involves the collection of plant, animal, or other materials from a natural setting:<br>• Field permit number<br>• Name of the institution or relevant body that granted permission	
**Data Availability**<br><br>Authors are required to make all data underlying the findings described fully available, without restriction, and from the time of publication. PLOS allows rare exceptions to address legal and ethical concerns. See the PLOS Data Policy and FAQ for detailed information.	Yes - all data are fully available without restriction

A Data Availability Statement describing where the data can be found is required at submission. Your answers to this question constitute the Data Availability Statement and **will be published in the article**, if accepted.<br><br>**Important:** Stating 'data available on request from the author' is not sufficient. If your data are only available upon request, select 'No' for the first question and explain your exceptional situation in the text box.<br><br>Do the authors confirm that all data underlying the findings described in their manuscript are fully available without restriction?	
**Describe where the data may be found in full sentences. If you are copying our sample text, replace any instances of XXX with the appropriate details.**<br><br>• If the data are **held or will be held in a public repository**, include URLs, accession numbers or DOIs. If this information will only be available after acceptance, indicate this by ticking the box below. For example: *All XXX files are available from the XXX database (accession number(s) XXX, XXX.*).<br>• If the data are all contained **within the manuscript and/or Supporting Information files**, enter the following: *All relevant data are within the manuscript and its Supporting Information files.*<br>• If neither of these applies but you are able to provide **details of access elsewhere**, with or without limitations, please do so. For example:<br><br>*Data cannot be shared publicly because of [XXX]. Data are available from the XXX Institutional Data Access / Ethics Committee (contact via XXX) for researchers who meet the criteria for access to confidential data.*<br><br>*The data underlying the results presented in the study are available from (include the name of the third party*	All data are in the manuscript and/or supporting information files.

and contact information or URL).<br>• This text is appropriate if the data are owned by a third party and authors do not have permission to share the data.<br><br>* typeset	
Additional data availability information:	

Manuscript Click here to access/download;Manuscript;Manuscript.docx

Expression of *OsORAP1* allele in rice (*Oryza sativa* L.) at ambient ozone stress in relation to ethylenediurea (EDU) application

Rigyan Gupta[1,2], Shamsul H. Prodhan[1], Md. Nazmul Hasan[1], Shamsun Nahar Begum[2], Mohammad Hasanuzzaman Rani[2], Mirza Mofazzal Islam[2], Md Ashrafuzzaman[1*]

[1]Department of Genetic Engineering & Biotechnology (GEB), School of Life Sciences, Shahjalal University of Science and Technology (SUST), Sylhet, Bangladesh

[2]Plant Breeding Division, Bangladesh Institute of Nuclear Agriculture (BINA), Mymensingh, Bangladesh

*Corresponding author

E-mail: azamanbt@gmail.com

Abstract

Rising tropospheric ozone concentrations pose a significant threat to crop production in many Asian countries like Bangladesh, necessitating the development of ozone-tolerant rice (*Oryza sativa* L.) varieties. This study investigates the response of *OsORAP1* allele in different rice varieties, particularly Kasalath-type and Nipponbare-type, to Ethylenediurea (EDU) treatment under ambient ozone stress. The experiment, conducted during the 2022 rainfed season, involved growing of 20 rice varieties under a split-plot design with and without EDU treatment, followed by genomic DNA collection at the vegetative stage to differentiate Kasalath-types and Nipponbare-types, and RNA extraction from 10 selected varieties at the flowering stage for gene expression analysis. The research revealed distinct genetic responses between the two types, with Nipponbare-type varieties showing significant increases in *OsORAP1* expression and more leaf bronzing score (LBS) following EDU treatment. In contrast, Kasalath-type varieties exhibited less pronounced responses due to their inherent tolerance to ozone stress. The effects of EDU on with Kasalath-type varieties responding notably in parameters such as ineffective tillers and straw yield, while Nipponbare-types showed significant changes in unfilled grains and grain yield. Additionally, differences were observed in how EDU treatment influenced reflectance indices parameters, indicating variability in how these varieties mitigate ozone stress. The study underscores the complex interaction between genetic factors, environmental conditions, and treatment in shaping the response of rice varieties to EDU, and suggests future research to further explore the genetic mechanisms, particularly the role of *OsORAP1*, that contribute to these differential responses.

Key words: Ozone, Stress, Ethylenediurea (EDU), *OsORAP1*, Rice, Food security, Global change

1. Introduction

The presence of tropospheric ozone (O_3) in the atmosphere is a major threat to crop production globally. Its high phytotoxicity and occurrence above agrarian regions make it a crucial atmospheric contaminant [1, 2, 3]. The formation of O_3 is a result of the photochemical reactions of precursor gases that are mainly emitted from anthropogenic sources such as nitrogen oxides, carbon monoxide, volatile organic compounds, and methane [4, 5, 6]. The adverse effects of ambient O_3 can lead to estimated yield losses of 4% globally and 10% regionally for rice [2, 7] and 4-17% for wheat, 10-14% for soybean, and 3-6% for maize, resulting in a projected annual economic loss of US$ 12-21 billion based on global crop models [8].

The harmful effects of O_3 on vegetation were first identified in North America during the 1950s and later observed in Europe and Japan in the 1970s [5, 9]. Ground-level O_3 concentrations have significantly increased in urban areas and are even higher in rural areas than in cities [10, 11]. The first recorded incident of photochemical smog and O_3 damage to vegetation was reported by Middleton *et al.* [12], while Richards *et al.* [13] found O_3 as a phytotoxic constituent of photochemical smog while investigating leaf injury in grape vines (*Vitis vinifera*) in Southern California.

Upon entering plant stomata, O_3 dissolves in the apoplast and generates different reactive oxygen species (ROS). These ROS can change cellular functions that result in premature senescence, cell death, and the up- or down-regulation of specific genes [14, 15]. Rice (*Oryza sativa* L.) is a staple crop in Asia but is severely affected by tropospheric O_3, particularly in

South and East Asia. Since its cropping season overlaps with peak O_3 concentrations, breeding O_3-tolerant rice varieties is necessary to ensure global food supply [16] as the demand for food is projected to double by 2050. The chemical compound Ethylenediurea (EDU; N- [2-(2-oxo-1-imidazolidinyl) ethyl]-N′-phenylurea) was initially introduced by Carnahan, Jenner, and Wat in 1978 [17] and has been utilized for a considerable time to assess the effects of O_3 on plants [18, 19, 20]. EDU is conveniently sprayed on leaves, works as an antiozonant, and provides a "control" without O_3 damage. The mode of action of EDU against O_3 is still unclear. It can be used to screen plants for O_3 sensitivity under ambient conditions, verify foliar O_3 symptoms in the field, and thus suppress O_3 injury in plants [19, 21].

Previous research has identified genetic factors associated with O_3 tolerance in rice, suggesting that this trait is controlled by multiple medium-effect loci rather than a single large-effect locus [22, 23]. In a study by Frei *et al.* [24] using a mapping population derived from the cultivars Nipponbare (susceptible) and Kasalath (tolerant), certain O_3-related quantitative trait loci (QTLs) were identified. One of the identified QTLs, OzT9, was associated with visible leaf symptom formation in response to O_3 stress, where the allele from the tolerant cultivar Kasalath conferred tolerance. The effectiveness of OzT9 was supported by SL41, a chromosomal segment substitution line carrying a chromosomal introgression from Kasalath at the OzT9 locus in the Nipponbare background, which showed fewer visible symptoms than Nipponbare [24]. Further transcriptomic analysis revealed differential gene expression profiles between Nipponbare and SL41A under O_3 stress [25]. Physiologically, apoplastic ascorbate (ASA) is a line of defense against O_3 entering the plant through stomata. Several studies, including Luwe *et al.* [26], Plöchl *et al.* [27], and Feng *et al.* [28], support this connection between the annotation of candidate genes and O_3 stress tolerance. Moreover, a previous study showed that an AO-overexpressing

tobacco plant affects O_3 tolerance adversely due to altered apoplastic reduced ASA content and ASA redox conditions [29]. This transformative evidence suggests that the expression of the putative AO gene is also associated with O_3 stress tolerance in rice and the formation of significant symptoms.

According to a study by Frei *et al.* [25], the expression of the *OsORAP1* (OZONE RESPONSIVE APOPLASTIC PROTEIN1) gene in response to O_3 stress was significantly lower in the O_3-tolerant SL41 variety compared to the cv. Nipponbare. The gene was located near a QTL and showed differential regulation between a sensitive parent and a tolerant introgression line. Additional analysis revealed that the promoter region of *OsORAP1* had widespread polymorphisms, with the OzT9 donor variety responsible for this variation between the O_3-susceptible cv. Nipponbare and the O_3-tolerant cv. Kasalath. *OsORAP1* encodes an apoplastic protein that is involved in cell death processes but has no ascorbate oxidase activity. The lower or no expression of *OsORAP1* enhanced O_3 tolerance in rice, leading to its designation as OZONE RESPONSIVE APOPLASTIC PROTEIN1. A previous study utilized a transgenic T-DNA insertion line to suppress *OsORAP1* expression and confer O_3 tolerance. However, it is unclear whether this suppression can also be achieved naturally through polymorphism. Generally, the Nipponbare type allele, carried by a group of rice varieties, showed a significantly higher level of foliar symptom formation, lipid peroxidation, and biomass loss when exposed to elevated O_3 in a controlled setting [25]. Furthermore, the Nipponbare types exhibited significantly higher induction of *OsORAP1* expression in response to O_3 stress, suggesting that *OsORAP1* plays a crucial role in regulating O_3 response and susceptibility or tolerance in rice.

Therefore, the aim of this study was to investigate the impact of O_3 stress on rice and to assess the potential of EDU as a bio-monitoring tool for O_3 in highly affected developing

countries such as Bangladesh. Since conventional O_3 experimental facilities like open top chamber (OTC) and free-air concentration enrichment (FACE) O_3 are lacking in these countries, this study aimed to evaluate the expression of *OsORAP1* gene and its correlation with O_3 tolerance when the plants are continuously exposed to an average of 70 ppb or more ambient O_3 applying EDU by collecting the leaf samples during the flowering stage to assess gene expression. The study was conducted based on 10 Bangladeshi rice varieties during their flowering stage with and without the application of EDU (300 ppm, once a week after transplanting until harvesting).

2. Materials and Methods

2.1 Experimental site and plant materials

The experiment was set in Bangladesh Institute of Nuclear Agriculture (BINA) HQs, Mymensingh farm (24° 43' N latitude and 90° 25' E longitude), Bangladesh which is situated in Old Brahmaputra Floodplain (AEZ-23) at rainfed (boro) season of 2022 (which is denoted as dry season usually depend on irrigated water and the crops transplanted from December to early February and harvested between April and June). A total of twenty high yielding popular (inbred and hybrid) and local diversified *indica and Aus* rice genotypes were used in this experiment, and 10 (Binadhan-15, Binadhan-17, BR11, Hutra, Kasalath, Binadhan-12, Binadhan-16, BRRI dhan28, BRRI dhan29, and BRRI hybrid dhan3) representative were selected for gene expression analysis with details provided in table 1.

2.2 Growing of varieties, design of the experiments and measurement of data

The seeds of twenty varieties were wrapped up in water container for 24 hours. After that, the seeds were removed from water and kept in gunny bags and leave in dark room. It was required about 3-5 days to germinate the all seed samples. Then, the germinated seed were sown in nursery beds with definite spacing and well tagged with a good layout. The experimental fields were prepared by ploughing and laddering with a tractor. By removing all the stubbles and weeds the field was then prepared. Then thirty-five day old seedlings were detached from the nursery bed with healthy root system. The experiments were than laid out as split-plot designs with four replicates arranged in blocks, including EDU treatment as the main plot (i.e. with and without EDU), and genotype as the subplot. Each subplot was 4 m^2 (2 × 2 m) size of which a central one m^2 area were used for yield estimation at harvest and the border plants may be used for destructive sampling during the growing season. After that, the seedling were transplanted in the main field at the rate one seedling per hill with a spacing 20 cm row to row distance and 15 cm hill to hill distance. Considering two treatments and four replicates, each experimental field was approximate size of 1050 m^2 (1000 m^2 experimental area plus dikes, bunds, irrigation channels, etc.). Urea, triple super phosphate, muriate of potash, gypsum, zinc sulphate and boron (the main source of N, P, K, S, Zn and B respectively) with a dose @ 270 Kg, 115 Kg, 150 Kg, 75 Kg, 6 Kg and 7 Kg, respectively were applied in the experimental field. During the final land preparation, Carbofuran-5G @ 10 Kg/ha (a granular insecticide) and all fertilizers excluding urea and zinc sulphate were applied; one-third of the urea dose was applied before the full dose of zinc sulphate was applied 12 days after transplanting. Again, urea were used in two split doses at 30 and 45 days after transplanting. For the reductions of insect and other pest infestation the pesticides were applied as when necessary. Three times of weeding were done at 15, 30 and 45 DAT.

Before each measurement, five randomly selected plants from each subplot were tagged. After harvesting, data were collected from a 1 m^2 area in each subplot, along with the five selected plants. The measured parameters included plant height (cm), total number of tillers per plant, number of effective and non-effective tillers per plant, panicle length (cm), filled grain count per plant, filled grain weight per plant, unfilled grain count per plant, unfilled grain weight per plant, grain yield, straw yield, and thousand-kernel weight. The harvest index (%) was measured by using the following formula:

$$\text{Harvest index (\%)} = \text{(Grain yield)/(Total biomass)} \times 100$$

A leaf bronzing score (LBS) ranging from 0 to 10 was used to assess ozone-induced leaf damage, following the method described by Ueda et al. [23]. A score of 0 represented no visible symptoms on any leaves, while a score of 10 indicated severe damage affecting the entire plant due to ozone stress.

Stomatal conductance was assessed using a leaf porometer (Model SC1, Decagon Devices, Pullman, WA) on the youngest fully expanded leaves of a selected plant per subplot. Measurements were taken on sunny days between 9:30 AM and 2:00 PM. To evaluate ozone stress, spectral reflectance was recorded with a handheld spectroradiometer (Polypen RP410, PRI, Czech Republic). The normalized difference vegetation index (NDVI) was calculated using the formula: $(R_{780} - R_{630}) / (R_{780} + R_{630})$. Similarly, the Lichtenthaler Index 2 (Lic2) was determined as the ratio of R_{440} to R_{690}, Simple ratio index (SR) = R_{NIR}/R_{RED}, Photochemical reflectance index (PRI) = $(R_{531} - R_{570})/(R_{531} + R_{570})$, Anthocyanin reflectance index (ARI) = $(R_{550})^{-1} - (R_{700})^{-1}$. Here, in the vegetation indices, R refers to reflectance and subscript indicates the wave bands in nanometers.

2.3 Plant material collection for extraction of Genomic DNA and analysis

Young, vigorously growing fresh leaf samples from the 20 rice genotypes were collected from the seedlings at vegetative stage (85 days old seedlings) to extract genomic DNA. Initially, healthy portion of the youngest leaves of the seedlings were cut apart with sterilized scissors and washed in distilled water and ethanol and dried on fresh tissue paper to remove spore of microorganisms and any other source of foreign DNA. The collected leaf samples were then put into polythene bags and kept on ice in an icebox. Then, the polythene bags were wrapped in aluminium foil and stored at -80^{0}C freezer.

Genomic DNA of the selected rice genotypes were extracted from leaf samples using a DNeasy Plant Mini Kit (Qiagen, Düsseldorf, Germany). For confirmation of genomic DNA, agarose gel electrophoresis were done by visualizing the DNA band in the Gel Doc system (Biometra, Japan). For the determination of quantity and purity the extracted samples were analyzed with Nanodrop 2000c (Thermo Fisher Scientific Inc., Wilmington, DE, USA). Then the DNA concentrations were adjusted to 25 ng/ µl. Then, PCR reactions were performed on each DNA sample in a 10µl reaction mix containing the following reagents: 1µl Ampli Taq polymerase buffer (10X), 2.5µl Primer (10µM), 1µl dNTPs (250µM), 0.2µl Ampli Taq DNA polymerase, 2µl Genomic DNA (25ng/µl) and 3.3µl Sterile deionized water. DNA amplification were performed in an oil-free thermal cycler. The reaction mix were preheated at 94^{0}C for 5 minutes followed by 35 cycles of 1 min denaturation at 94^{0}C, 1 min annealing at 54^{0}C and, elongation or extension at 72^{0}C for 2 minutes. After the last cycle, a final step for 7 minutes at 72^{0}C were allowed to complete the extension of all amplified fragments. After completion of the

cycling program, reactions were held at 4^0C. The primer KAS_1_2 sequence that were used for the initial screening is 5´-GCCTTCCTCCTTGTGGTCG-3´ (forward) and 5´-AGGGAACGTCCCACTGTTG-3´ (reverse) for the Kasalath-type *OsORAP1* allele (annealing temperature 57 °C).

Again, agarose gel electrophoresis were done by ethidium bromide staining for visualizing the band (Kasalath-type) in the Gel Doc system (Biometra, Japan). Among the twenty genotypes the selection was based on the presence and absence of a PCR band [30] for further analyses (Table 1). Based on PCR bands we classified the varieties into two distinct clades: Kasalath-types and Nipponbare-types and found that among the 20 rice varieties, 8 varieties showed PCR bands of were Kasalath-type *OsORAP1* allele and the remaining 12 varieties which were not show PCR bands of were Nipponbare-type *OsORAP1* allele [30].

Table 1: Brief description, collection and/or origin of the rice varieties investigated in this experiment carrying different alleles at the *OsORAP1* locus using primer KAS_1_2 [30]

Sl. No.	Variety name	Short description	Collection and/or origin	Subspecies	*OsORAP1* allele
1	Iratom-24	Early maturing high-yielding variety (HYV)	BINA / Bangladesh	*Indica*	Nipponbare-type
2	Binadhan-6	HYV	BINA / Bangladesh	*Indica*	Kasalath-type
3	Binadhan-10	Salt tolerant HYV	BINA / Bangladesh	*Indica*	Nipponbare-type

4	Binadhan-11	Submergence tolerant	BINA / Bangladesh	*Indica*	Nipponbare-type
5	Binadhan-12	Submergence tolerant (fine grain)	BINA / Bangladesh	*Indica*	Nipponbare-type
6	Binadhan-14	Late sowing potential HYV	BINA / Bangladesh	*Indica*	Kasalath-type
7	Binadhan-15	Premium quality	BINA / Bangladesh	*Indica*	Kasalath-type
8	Binadhan-16	Short duration HYV	BINA / Bangladesh	*Indica*	Nipponbare-type
9	Binadhan-17	HYV	BINA / Bangladesh	*Indica*	Kasalath-type
10	BR11	Widely grown HYV	BRRI / Bangladesh	*Indica*	Kasalath-type
11	BRRI dhan28	Early maturing HYV	BRRI / Bangladesh	*Indica*	Nipponbare-type
12	BRRI dhan29	HYV	BRRI / Bangladesh	*Indica*	Nipponbare-type
13	BRRI dhan88	Early maturing HYV	BRRI / Bangladesh	*Indica*	Nipponbare-type
14	BRRI hybrid dhan1	High-yielding hybrid variety	BRRI / Bangladesh	*Indica*	Nipponbare-type

15	Hutra	Landrace	Chapainawabganj/ Bangladesh	*Aus*	Kasalath-type
16	Tepiboro	Landrace	Chapainawabganj/ Bangladesh	*Aus*	Kasalath-type
17	BRRI hybrid dhan3	High-yielding hybrid variety	BRRI / Bangladesh	*Indica*	Nipponbare-type
18	IR64	International mega-variety	BRRI / IRRI, Philippines	*Indica*	Nipponbare-type
19	Kasalath	Landrace	BRRI / Bangladesh	*Aus*	Kasalath-type
20	BRRI dhan58	HYV	BRRI / Bangladesh	*Indica*	Nipponbare-type

Note: BINA = Bangladesh Institute of Nuclear Agriculture, BRRI = Bangladesh Rice Research Institute and IRRI = International Rice Research Institute

2.4 Ozone treatment and monitoring

After transplanting, the two treatments viz. with EDU (Ethylenediurea) and without EDU (control) were applied and plants were continuously exposed to natural field conditions of BINA HQs farm, Mymensingh, Bangladesh where the ambient ozone concentration was more than 70 ppb, quantified by a well handheld ozone device (series 500; Aeroqual Ltd. Auckland, New Zealand) at every day after transplanting. The chemical EDU at the concentration of 300 ppm once a week were applied as a foliar spray in the morning (9 am to 12 pm) to mitigate the ozone effects [16]. The controls plant were sprayed with similar of water which was needed for spraying EDU.

2.5 Collection of leaf samples for RNA isolation and gene expression analyses

At the flowering stage (115 days old) between 10 a.m. and 12 p.m., the leaf samples of the selected rice varieties viz. 5 Kasalath-types varieties (Binadhan-15, Binadhan-17, BR11, Hutra, and Kasalath) and 5 Nipponbare–type (Binadhan-12, Binadhan-16, BRRI dhan28, BRRI dhan29, and BRRI hybrid dhan3) were collected. Two randomly selected plants of each genotype from each treatment plot were collected and leaves were pooled for one representative sample. Then the samples were flash-frozen in liquid nitrogen and stored at −80 °C for RNA extraction.

Again, each genotype among the 10 genotypes was extracted from 3 replicates (of 4) of each treatment, yielding a total of 6 samples. In the next step, whole leave samples were ground in liquid nitrogen to a fine powder, and total RNA was extracted with the Promega RNA Isolation Kit containing RQ1 DNase (Promega). In order to determine the quantities and purity of the extracted samples, spectrophotometers NanoDropTM 2000/2000c (Thermo Fisher Scientific Inc., Wilmington, DE, USA) was used (Thermo Fisher Scientific Inc., Wilmington, DE, USA). Reverse transcription of 300 ng of total RNA was performed using TAKARA PrimeScriptTM 1st strand cDNA Synthesis Kit (Cat. # 6110A). In accordance with the manufacturer's instructions, quantitative PCR was performed with TAKARA TB Green Premix Ex Taq (Tli RNase H Plus) (Cat. # RR420A). In the reaction mixture, there were 7.5µl of TB Green Premix Ex Taq (Tli RNaseH Plus) (2X), 0.4µl of each gene-specific primer (10 µM), 0.2µl of ROX Reference Dye (50X), 4.5µl of nuclease-free water, and 2µl of cDNA Template (15 ng) sample. The following thermal conditions prevailed: denaturation at 95 °C for 10 min, 40 cycles

of 15 s of denaturation at 95 °C, and 1 min of annealing and extension at 60 °C. After each reaction, melting curves were analyzed to verify primer specificity. In this study, the housekeeping gene named 18S rRNA (AK059783) was amplified using 5´-CTACGTCCCTGCCCTTTGTACA-3´ (forward) and 5´-ACACTTCACCGGACCATTCAA-3´ (reverse) cast-off as an endogenous reference [31]. A delta-delta (Δ-Δ) CT quantification method was applied to quantify target genes according to Frei *et al.* [25]. Based on a cDNA dilution series, the efficiency of amplification was more than 90% for the primer pair. Analytical duplicates were used for gene expression analyses. An expression analysis of *OsORAP1* was conducted using the primer pair's 5´-CATCGAGGCGCACTTCTT-3´ (forward) and 5´-CGAACGGCTAGCTTCTGGT-3´ (reverse).

2.5 Statistical analysis

For analysis of variance the experimental data were subjected to mixed model analysis using the package lme4 in R [32] with Satterthwaite's method where EDU treatment, variety, and their interaction were set as fixed effects. Pairwise comparisons of individual varieties with and without EDU treatment were obtained using the procedure emmeans in R and adjusted using the Tukey's method. The total analysis was conducted in R 4.2.1 (R Core Team 2022) with R packages lmerTest [33], multcomp [34] and performance [35].

3. Results

3.1 Ozone concentrations levels in study site

During whole growing period the average ozone concentrations were more than 70 ppb (76.49 ppb) where the threshold level of ozone for crop production is 40 ppb [36]. Daily average

ozone concentration were given in Fig 1 where it was found that the ozone concentrations was more than the threshold level which drastically affects the production of rice in Mymensingh, Bangladesh.

Fig 1. Daily average ozone concentration in experimental location (Myemensingh) in the *Boro* rice growing seasons 2022.

3.2. Varietal differences in the responsiveness of *OsORAP1* expression to EDU treatment in rice

This study analyzed the *OsORAP1* locus among different rice varieties and revealed two distinct clades of varieties: the Nipponbare-type and the Kasalath-type allele. To investigate the effect of EDU on mRNA expression, 10 rice varieties were grown and exposed to ambient O_3 stress in a field condition where the O_3 concentration was higher than 70 ppb. The Kasalath and Nipponbare types both showed highly significant (P <0.001) *OsORAP1* expression in EDU treatment, variety and, EDU treatment and varietal interaction which indicate the EDU have effect on *OsORAP1* expression. (Table 2).

Table 2. Analysis of variance (ANOVA) of different traits (Molecular, Agronomic and physiological) of ten selected rice varieties grown in ozone-polluted location of Mymensingh, Bangladesh in rainfed'22 season with the application of EDU or without application of EDU (control)

Traits	ANOVA						Treatment Means	
	EDU-Treatment		Variety		Interaction		Control	EDU
	Pr(>F)	F values	Pr(>F)	F values	Pr(>F)	F values		

**Kasalath-type**								
Molecular								
OsORAP1 Expression	<0.000***	19.483	<0.000***	30.415	<0.000***	31.076	0.567	1.508
Agronomic								
Leaf bronzing score (lbs)	1.000	0.000	<0.000***	6.565	0.074	2.411	2.400	2.400
No. of effective tiller plant^{-1}	0.081	3.272	0.032*	3.038	0.157	1.790	9.170	9.950
No. of ineffective tiller plant^{-1}	0.023*	5.845	0.002**	5.681	0.824	0.376	0.800	0.570
No. of filled grain plant^{-1}	<0.000***	17.871	<0.000***	42.807	0.723	0.518	1362.000	1522.490
No. of unfilled grain plant^{-1}	0.366	0.847	<0.000***	22.610	0.250	1.433	537.120	515.580
Thousand kernel weight (TKW)	0.783	0.077	<0.000***	1536.219	0.979	0.107	24.076	24.063
Grain yield plant^{-1}	0.144	2.253	0.001**	5.902	0.020*	3.435	274.333	291.318
Straw yield plant^{-1}	<0.000***	7.729	<0.000***	8.136	0.404	1.038	18.824	20.987
Harvest index (%)	0.554	0.358	0.015*	3.661	0.313	1.244	52.316	51.433
Physiological								
NDVI	0.052	3.868	<0.000***	20.637	0.044	2.539	0.690	0.698
SR	0.028*	4.950	<0.000***	20.622	0.053	2.411	5.503	5.683
SIPI	0.245	1.368	<0.000***	24.909	0.111	1.928	0.754	0.756
Lic2	0.010*	6.803	<0.000***	10.389	0.376	1.068	0.675	0.690
PRI	0.432	0.620	<0.000***	8.533	0.250	1.367	0.055	0.056
**Nipponbare-type**								
Molecular								
OsORAP1 Expression	<0.000***	65.595	<0.000***	15.000	<0.000***	9.512	3.839	1.270
Agronomic								
Leaf bronzing score (lbs)	<0.000***	128.000	<0.000***	21.375	0.363	1.125	3.900	2.300
No. of effective tiller plant^{-1}	0.670	0.185	0.035*	2.957	<0.000***	8.999	10.270	10.090
No. of ineffective tiller plant^{-1}	0.364	0.850	0.049*	2.701	0.935	0.202	0.870	0.710
No. of filled grain plant^{-1}	0.172	1.962	<0.000***	122.829	<0.000***	4.429	1563.730	1490.400
No. of unfilled grain plant^{-1}	0.009**	7.711	<0.000***	19.897	0.177	1.696	556.510	385.123
Thousand kernel weight (TKW)	0.154	2.153	<0.000***	2387.456	0.408	1.033	23.263	23.096
Grain yield plant^{-1}	0.031*	5.176	<0.000***	35.249	0.035*	3.026	245.273	268.394
Straw yield plant^{-1}	0.018	6.345	<0.000***	11.885	0.066	2.502	20.394	24.297
Harvest index (%)	0.507	0.451	<0.000***	14.024	0.243	1.448	51.895	53.072
Physiological								
NDVI	0.219	1.527	<0.000***	6.091	0.192	1.554	0.672	0.680

SR	0.304	1.067	<0.000***	6.920	0.138	1.780	5.202	5.318
SIPI	0.180	1.818	<0.000***	6.987	0.380	1.060	0.738	0.744
Lic2	0.632	0.231	0.012*	3.328	0.146	1.743	0.682	0.686
PRI	0.267	1.241	<0.000***	9.358	0.893	0.276	0.041	0.045

Note: NDVI = Normalized difference vegetation index, SR = Simple ratio, SIPI = Structure intensive pigment index, Lic2 = Lichtenthaler indices 2, PRI = Photochemical reflectance index.

Further analysis showed that within the varieties, Kasalath-type varieties showed less *OsORAP1* expression in both EDU treated varieties and control varieties except Kasalath, while the Nipponbare-types showed higher significant *OsORAP1* expression in control varieties than EDU treated varieties except Binadhan-16 and BRRI hybrid dhan3 which showed higher *OsORAP1*expression but not significant (Fig 2). These results suggest that the expression of *OsORAP1* is more responsive to EDU application in Nipponbare-type varieties than Kasalath-type varieties.

Fig 2. Expression of *OsORAP1* in 5 Kasalath-type and 5 Nipponbare-type rice varieties exposed to ambient ozone stress with the treatment of EDU and without EDU (control) at Real-time PCR data. Means values and standard errors (n = 3) are shown. Asterisks indicate significant differences. *, $P < 0.05$; **, $P < 0.01$; ***, $P < 0.001$; no asterisks means not significant.

3.3 Responses to ambient O_3 stress of the varieties carrying different alleles at *OsORAP1* locus due to EDU application

3.3.1. Leaf bronzing score

Upon prolonged exposure to ambient O_3 stress during the flowering or late flowering stage, visible symptoms of O_3 damage were observed. The formation of visible symptoms due to ambient ozone stress was found to differ significantly between the two allele groups when EDU treatments were applied. Nipponbare-type varieties displayed highly significant response, while

Kasalath-type varieties showed insignificant response in EDU treatment. Again, Kasalath-type varieties showed insignificant response within the varieties and interaction between varieties and EDU treatment. On the other hand, Nipponbare-type varieties showed highly significant response within the variety but insignificant between varietal and EDU treatment interactions (Table 2).

In Nipponbare-type varieties, the response of EDU was prominent and the score in control plots were higher and significant, where the EDU treated varieties plots scores were less. Kasalath-type varieties response to EDU treatment were not significant except Kasalath because this variety is highly tolerant to ozone stress [16, 37] and not responsive to EDU this is why the EDU treated Kasalath variety gave significant response while the control Kasalath variety did not (Fig 3).

Fig 3. Leaf bronzing scores of 5 Kasalath-type and 5 Nipponbare-type rice varieties exposed to ambient ozone concentration with and without application of ethylenediurea (EDU); bars indicate mean values of Mymensingh for Boro season 2022 with four replicates and standard errors (n=4); asterisks above the bars indicate significant difference between control and EDU treatment with one variety by Tukey's test (* $p<0.05$, **$p<0.01$, ***$p<0.001$).

3.3.2. Yield parameters

The effects of Ethylenediurea (EDU) on yield parameters were investigated in both Kasalath-type and Nipponbare-type rice varieties, revealing varying responses across different parameters and varieties. In Kasalath-type varieties, significant responses to EDU treatment were observed for parameters including the number of ineffective tillers plant^{-1}, number of filled grains plant^{-1}, and straw yield plant^{-1}. However, other parameters such as the number of effective

tillers plant^{-1}, number of unfilled grains plant^{-1}, thousand kernel weight (TKW), grain yield plant^{-1}, and harvest index (%) did not display significant responses to EDU treatment. Within the Kasalath-type varieties, responses were highly significant for all studied characters, but the interaction between EDU treatment and varieties was not significant except for grain yield plant^{-1}. Similarly, in Nipponbare-type varieties, significant responses to EDU treatment were observed for parameters such as the number of unfilled grains plant^{-1} and grain yield plant^{-1}. Conversely, other parameters like the number of effective tillers plant^{-1}, number of ineffective tillers plant^{-1}, number of filled grains plant^{-1}, TKW, straw yield plant^{-1}, and harvest index (%) did not exhibit significant responses to EDU treatment. Within the Nipponbare-type varieties, responses were highly significant for all studied characters, and the interaction between EDU treatment and varieties was highly significant for parameters including the number of effective tillers plant^{-1}, number of filled grains plant^{-1}, and grain yield plant^{-1} and other parameters were not significantly response (Table 2).

Specifically focusing on grain yield plant^{-1} and straw yield plant^{-1}, Kasalath-type varieties generally did not exhibit significant gains or losses in response to EDU treatment, except for BR11, which showed a significant increase in both parameters. Conversely, in Nipponbare-type varieties, BRRI hybrid dhan3 and BRRI dhan29 exhibited significant gains in grain yield plant^{-1} and straw yield plant^{-1} in response to EDU treatment, while other varieties did not show significant changes in either parameter (Table 3, Fig 4a and 4b).

Table 3. Parameters of contrasting rice varieties exposed to control (without EDU) and EDU treated varieties in ambient ozone stress conditions.

**Sl. No.**	**Name of the varieties**	**Grain yield plant^{-1} (g)**			**Straw yield plant^{-1} (g)**			**Harvest Index (%)**		
		**Control**	**EDU**	**Gain /**	**Control**	**EDU**	**Gain**	**Control**	**EDU**	**Gain /**

		**(Without EDU)**	**treated**	**Loss (%)**	**(Without EDU)**	**treated**	**/ Loss (%)**	**(Without EDU)**	**treated**	**Loss (%)**
**Varieties with Kasalath-type *OsORAP1* allele**										
**1**	Binadhan-15	27.11	28.76	6.08	16.63	19.08	14.73	53.11	49.48	-7.34
**2**	Binadhan-17	27.11	26.74	-1.37	19.47	20.29	4.21	57.95	56.90	-1.84
**3**	BR11	27.92	37.66	34.88	20.85	25.89	24.17	47.69	53.17	10.31
**4**	Hutra	24.63	24.33	-1.22	16.35	18.21	11.38	51.91	49.13	-5.67
**5**	Kasalath	30.38	28.17	-7.27	20.82	21.46	3.07	50.92	48.49	-5.02
**Average**		27.43	29.13	6.20	18.82	20.99	11.51	52.32	51.43	-1.91
**Varieties with Nipponbare-type *OsORAP1* allele**										
**6**	Binadhan-12	23.82	23.26	-2.35	19.19	24.55	27.93	51.34	47.72	-7.59
**7**	Binadhan-16	25.40	26.09	2.71	15.19	11.66	-23.23	59.62	64.08	6.96
**8**	BRRI dhan28	18.16	16.48	-9.25	21.15	21.23	0.38	44.69	41.73	-7.10
**9**	BRRI dhan29	23.05	28.65	24.29	25.01	33.66	34.58	46.46	53.79	13.62
**10**	BRRI hybrid dhan3	32.35	39.72	22.78	21.43	30.38	41.76	57.37	58.05	1.71
**Average**		24.53	26.84	9.41	20.39	24.30	19.13	51.90	53.07	1.52

Regarding the parameter harvest index (%), neither Kasalath-type nor Nipponbare-type varieties exhibited significant gains or losses due to EDU treatment (Table 3, Fig 4c). So, the variability in the response of different rice varieties to EDU treatment in terms of yield parameters, with some varieties exhibiting significant improvements while others show no significant response.

Fig 4. Yield parameters [a. Grain yield plant^{-1} b. Straw yield plant^{-1} and c. Harvest index (%)] of 5 Kasalath-type and 5 Nipponbare-type rice varieties grown in ambient ozone concentration with and without application of ethylenediurea (EDU); bars indicate mean values of Mymensingh for rainfed season 2022 with four replicates and standard errors (n=4); asterisks above the bars indicate significant difference between control and EDU treatment with one variety by Tukey’s test (* p<0.05, **p<0.01, ***p<0.001).

3.2.3. Reflectance indices

The impact of the EDU application on alleviating ambient ozone stress varied significantly across different parameters and rice varieties. Among Kasalath-type varieties, significant effects were observed for the parameters simple ratio (SR) and Lichtenthaler indices 2 (Lic2), while other reflectance indices such as normalized difference vegetation index (NDVI), structure intensive pigment index (SIPI), and photochemical reflectance index (PRI) did not show significant responses to the EDU treatment. However, within Kasalath-type varieties, notable responses were observed, indicating a significant interaction between varieties and EDU treatment across all studied reflectance indices parameters. Conversely, in Nipponbare-type varieties, the EDU treatment and its interaction with varieties did not yield significant responses across any of the studied reflectance indices parameters. Nonetheless, significant differences were observed among the varieties themselves for all parameters, suggesting distinct genetic responses irrespective of the EDU treatment (Table 2).

Regarding the parameter simple ratio (SR), indicative of vegetation health, Kasalath-type varieties generally did not exhibit significant responses to the EDU treatment, except for Binadhan-17. Notably, Binadhan-17 displayed a significant response to EDU, possibly influenced by specific alleles or other abiotic factors. Conversely, Nipponbare-type varieties did not show significant responses to the EDU treatment, although there was an overall improvement in leaf greenness, except for Binadhan-12, possibly influenced by genetic or environmental factors (Fig 5a). For the structure intensive pigment index (SIPI), estimating the ratio of carotenoids to chlorophyll, both Kasalath-type and Nipponbare-type varieties displayed insignificant responses to the EDU treatment. However, Kasalath-type varieties showed higher responses overall to EDU, except for Kasalath itself. In contrast, Nipponbare-type varieties

indicated higher responses to EDU, except for Binadhan-12, suggesting variability in response possibly due to genetic or environmental influences (Fig 5b). "These findings suggest that EDU application can provide some degree of protection against ambient ozone stress in Nipponbare-type varieties. Although the variation for certain reflectance indices under treatment and interaction effects was not statistically significant, trends in leaf greenness and photosynthesis-related parameters indicate a positive influence of EDU. Overall, the study underscores the variability in the response of rice varieties to the EDU treatment in mitigating ambient ozone stress, influenced by genetic factors, environmental conditions, and interactions between varieties and treatments.

Fig 5. Physiological traits [a. Simple ratio (SR) and b. Structure insensitive pigment index (SIPI)] of 5 Kasalath-type and 5 Nipponbare-type rice varieties grown in ambient ozone concentration with and without application of ethylenediurea (EDU); bars indicate mean values of Mymensingh for rainfed season 2022 with four replicates and standard errors (n=4); asterisks above the bars indicate significant difference between control and EDU treatment with one variety by Tukey’s test (* $p<0.05$, **$p<0.01$, ***$p<0.001$).

4. Discussion

4.1. Detrimental ambient ozone stress and *OsORAP1* expression in different rice varieties

Rice is an important staple food for more than half of the world's population [38]. However, rice production is threatened by various environmental stresses, such as ozone pollution [39]. Ozone is a major air pollutant that causes significant damage to crops, including

rice [40, 41]. The rising tropospheric ozone concentrations in South Asia is very explicit [41]. It is one of the most important stress for rice production because rice production is now greatly hampered this region [16]. All of the varieties that we selected for our study were based on Bangladeshi rice production and these are the popular varieties of Bangladesh. To study this, we selected 10 rice varieties where 5 were carrying Kasalath type allele and 5 were carrying Nipponbare type allele. The OzT9 locus, previously identified as a QTL affecting foliar symptom formation in ozone stress, was used as a marker for ozone tolerance [24]. The ozone exposure level is continuously observed in crop-growing environments in South Asia, and daytime ozone peaks exceeding 80 ppb frequently occur, especially from February to April [42]. Still, during the study period i.e. in rainfed season, it was observed that the daytime ozone peaks in Mymensingh, Bangladesh, exceeded the average 70 ppb (Fig 1). Thus, the ambient ozone exposure in the field is important for breeding ozone-tolerant rice on the South Asian subcontinent, especially Bangladesh. In this study comparing *OsORAP1* expression between Kasalath and Nipponbare rice types under EDU treatment, both types exhibited highly significant differences ($P < 0.001$) in response to the treatment, as well as in varietal differences and their interactions (Table 1). Further analysis revealed that within the Kasalath-type group, there was generally lower *OsORAP1* expression in both EDU-treated and control varieties, except for the Kasalath variety itself, which showed high tolerance and did not respond to EDU treatment (not responsive to EDU) [36]. On the other hand, the Nipponbare-types displayed higher *OsORAP1* expression in the control varieties compared to EDU-treated ones. Notably, Binadhan-16 and BRRI hybrid dhan3, within the Nipponbare group, exhibited higher *OsORAP1* expression, though these increases were not statistically significant (Fig 2). This suggests a

complex interaction between variety type and response to EDU treatment, with Kasalath showing inherent tolerance and Nipponbare-types being more sensitive to the conditions tested.

One of the mechanisms by which plants respond to ozone stress is through the upregulation of the expression of various genes involved in stress tolerance. One such gene is *OsORAP1*, which is involved in oxidative stress response and has been shown to be upregulated in response to ozone stress in rice. Previous studies have shown that the presence of the QTL affects traits of agronomic importance such as spikelet sterility [44] and chalkiness [45] and the quality of rice straw [46]. Although the QTL has not been fine mapped or cloned to date, experiments with rice mutants suggested that the gene *OsORAP1* may be underlying the QTL effect [7]. We analyzed the *OsORAP1* gene in 20 rice varieties and found that the polymorphisms occurring in the original two contrasting clades are largely conserved. We observed a dramatic difference in terms of foliar symptom formation between the two allele classes, Kasalath type and Nipponbare type (Fig.3). The likely mechanism underlying this phenomenon is that *OsORAP1* is involved in the oxidative cell death cycle, which leads to the induction of programmed cell death in ozone stress [47]. The transcriptional regulation of *OsORAP1* appears to constitute an important switch for the induction of programmed cell death under ozone stress in rice. This information is crucial for rice breeders to develop ozone tolerant rice varieties that can withstand the rising tropospheric ozone concentrations in South Asia.

4.2. Response of EDU treatment on different alleles at *OsORAP1* locus for yield and physiological parameters

Tropospheric ozone concentration is increasing throughout the Asian countries due to influence of the activities of human, population growth and rapid development of industry and

economy [48]. Thus it is very crucial hazardous gases concisely effecting the agricultural crop production [49] now and even in the future, the crop production will be challenged without using tolerant varieties [22]. Several studies have demonstrated that EDU treatment can effectively mitigate the negative impact of ambient O_3 stress on plant growth, yield, and physiological processes of rice [16, 37, 50]. The underlying mechanism behind the protective role of EDU treatment on plants is thought to be due to its ability to scavenge reactive oxygen species and reduce oxidative damage in plants by protects the surface [51]. The Leaf Bronzing Score (LBS) is commonly employed as a phenotypic measure to assess variances in rice genotypes regarding their tolerance to diverse abiotic stresses, including ozone stress, zinc (Zn) deficiency, salinity, or iron toxicity [52, 53, 54]. Leaf bronzing score which is the marker of oxidative stress enriched the tolerant mechanism of Kasalath-type *OsORAP1* allele where the Nipponbare-type *OsORAP1* allele boosted the expression of gene in hazardous ozone concentrated area. Similarly, in our study, after application of EDU to the Kasalath-type and Nipponbare-type varieties, Kasalath-type varieties show tolerant i.e. less/no leaf bronzing score to EDU and insignificant response while the Nipponbare-type showed highly significant response and higher leaf bronzing score (Table 2). So, the Nipponbare-type varieties were EDU responsive i.e. EDU protects the plants from prolonged cell death [47] and Kasalath-type varieties were less/no EDU responsive i.e. the plants tolerant mechanism itself protects the plants from visible leaf injury (Fig 3). Different antioxidative defense mechanisms may account for the differential response of these two allele groups to ambient ozone stress. The findings of the present study are consistent with previous research that reported the efficacy of EDU treatment in reducing the adverse effects of ambient O_3 stress in plants [55].

EDU treatment in EDU responsive varieties i.e. Nipponbare type varieties increases the grain yield of rice moderately or not significantly [37, 49, 55]. In Kasalath-type rice varieties, notable reactions in Kasalath-type *OsORAP1* allele to the application of EDU treatment were noted across various parameters. Conversely, in Nipponbare-type rice varieties, distinct responses to the EDU treatment were observed in Nipponbare-type *OsORAP1* allele, primarily reflected in alterations in the number of unfilled grains plant^{-1} and the resultant grain yield plant^{-1} (Table 2). Focusing specifically on grain yield per plant and straw yield per plant, the response to EDU treatment varied among Kasalath-type rice varieties. Generally, there were no substantial gains or losses observed, with the exception of BR11, which exhibited a notable increase in both parameters following the treatment may be due to genetic or environmental influences i.e. involvement of some other allele responsible other than *OsORAP1* allele. In contrast, among Nipponbare-type rice varieties, BRRI hybrid dhan3 and BRRI dhan29 showed significant increases in grain yield per plant and straw yield per plant in response to EDU treatment in Nipponbare-type *OsORAP1* allele similar to observations reported by Ashrafuzzaman *et al.* [37] in BRRI dhan28 (BR28), while other varieties did not demonstrate significant changes in either parameter (Table 3, Fig 4a and 4b) and other varieties did not exhibit statistically significant changes. However, other Nipponbare-type varieties still demonstrated higher grain and straw yields, which may be influenced by genetic or environmental factors. Similarly, the cultivars such as BRRI dhan28 and BRRI dhan29 underwent prior evaluation in controlled environmental trials regarding their reaction to ozone, which were extensively cultivated varieties tailored for the rainfed season, exhibited a yield reduction of 30 to 40% in open-top chamber experiments when exposed to ozone levels ranging from 77 to 100 ppb throughout the growth period [37, 57]. By increasing the number of filled grains per plant, EDU increases the yield of rice varieties [16]

with Nipponbare-type *OsORAP1* alleles. EDU treatment has diverse effects on *OsORAP1* alleles in Kasalath and Nipponbare rice varieties, emphasizing the importance of tailoring agricultural practices for optimal yield outcomes. In Table 3, the reduction in yield and related parameters upon EDU treatment in Kasalath-type genotypes (Binadhan-17, Hutra, and Kasalath), as well as in some Nipponbare-type genotypes, may indicate that EDU has little to no protective effect on ozone-tolerant genotypes. These observations underscore the complex interplay of genetic makeup and environmental conditions in determining the physiological responses of rice varieties to agricultural treatments.

The impact of EDU application on mitigating ambient ozone stress varied significantly across parameters and the Kasalath-type and Nipponbare-type rice varieties. To assess the physiological yield response to EDU in both allele varieties, various reflectance indices were gathered. NDVI was employed to assess chlorophyll levels at both the leaf and canopy or ecosystem scales [37]. In our study NDVI, SIPI and PRI were not significant due to EDU treatment in both the allele groups but showed higher response (Table 2) and in previous studies, NDVI was significantly increased by EDU treatment in rice [16], wheat [58] and soybeans [59]. Previously, Lic2 has been proposed as a reliable physiological marker of ozone stress, conducive to high-throughput and non-invasive measurement through spectral reflectance [60]. Similarly, PRI has been recommended as a remote sensing technique for detecting ozone stress in vegetation before visible symptoms manifest [61]. Among Kasalath-type varieties, significant effects were noted for SR and Lic2, with notable interactions observed across all reflectance indices. Conversely, in Nipponbare-type varieties, no significant responses were observed for the studied reflectance indices (Table 2). While SIPI and SR responses were insignificant, Kasalath-type generally exhibited higher responses except Kasalath itself, contrasting with Nipponbare-type (Fig 5a and

5b). These results highlight the nuanced interactions between EDU treatment and rice varieties in combating ozone stress, emphasizing the role of genetic and environmental factors responsible for the EDU response in Kasalath-type and Nipponbare-type varieties. These findings have important implications for the selection of rice varieties for cultivation in areas with high ambient ozone levels, and underscore the need for further research to elucidate the molecular mechanisms underlying the observed differences in response to ozone stress between these two allele groups.

5. Conclusions

The response of different rice varieties, specifically Kasalath-type and Nipponbare-type, to Ethylenediurea (EDU) treatment under ambient ozone stress showed distinct genetic responses between these varieties. Nipponbare-type varieties displayed significant increases in *OsORAP1* expression and leaf bronzing score in response to EDU treatment, while Kasalath-type varieties showed less significant responses due to their inherent tolerance to ozone stress. Additionally, the effects of EDU on yield parameters varied among the varieties. Kasalath-type varieties showed significant responses in certain parameters like the number of ineffective tillers plant^{-1} and straw yield plant^{-1}, whereas Nipponbare-type varieties responded significantly in parameters such as the number of unfilled grains plant^{-1} and grain yield plant^{-1}. Furthermore, the impact of EDU treatment on alleviating ambient ozone stress differed across reflectance indices parameters, with notable variability observed between Kasalath-type and Nipponbare-type varieties. Overall, the study highlights the intricate interplay of genetic factors, environmental conditions, and treatment interactions in influencing the response of rice varieties to EDU treatment in mitigating ambient ozone stress. Building on the findings of this study, future

research should aim to identify the genetic mechanisms and also the understanding the role of *OsORAP1* that drive the differential responses of Kasalath-type and Nipponbare-type rice varieties to EDU treatment under ambient ozone stress, to enhance ozone tolerance and improve yield stability.

Acknowledgment

The author thanks to Professor Dr. Michael Frei, Department of Agronomy and Crop Physiology, Justus-Liebig-University, Giessen, Germany, for his continuous support including finance for the field experiment and his lab research facilities.

References

1. Agathokleous E, Koike T, Watanabe M, Hoshika Y, Saitanis CJ. Ethylenediurea (EDU), an effective phytoproctectant against O_3 deleterious effects and a valuable research tool. J. Agric. Meteorol. 2015; 71: 185-195. http://dx.doi.org/10.2480/agrmet.D-14-00017.
2. Ainsworth EA. Understanding and improving global crop response to ozone pollution. Plant. J. 2016; 90: 886–897. https://doi.org/10.1111/tpj.13298.
3. Tai APK, Val Martin M, Heald CL. Threat to future global food security from climate change and ozone air pollution. Nat. Clim. Chang. 2014; 4: 817–821. https://doi.org/10.1038/nclimate2317.

4. Paoletti E, Castagna A, Ederli L, Pasqualini S, Ranieri A, Manning WJ. Gene expression in snapbeans exposed to ozone and protected by ethylenediurea. Environ. Pollut. 2014; 193: 1–5. https://doi.org/10.1016/j.envpol.2014.06.001.
5. The Royal Society. Ground-level ozone in the 21st century: future trends, impacts and policy implications. 2008. The Royal Society, London, UK. https://royalsociety.org/-/media/policy/publications/2008/7925.pdf.
6. Yamaji K, Ohara T, Uno I, Tanimoto H, Kurokawa J, Akimoto H. Analysis of the seasonal variation of ozone in the boundary layer in East Asia using the Community Multi-scale Air Quality model: what controls surface ozone levels over Japan? Atmos. Environ. 2006; 40: 1856–1868. https://doi.org/10.1016/j.atmosenv.2005.10.067.
7. Ueda Y, Siddique S, Frei M. A novel gene, OZONE-RESPONSIVE APOPLASTIC PROTEIN1, enhances cell death in ozone stress in Rice. Plant Physiol. 2015b; 169: 873–889. https://doi.org/10.1104/pp.15.00956.
8. Avnery S, Mauzerall DL, Liu J, Horowitz LW. Global crop yield reductions due to surface ozone exposure: 2. Year 2030 potential crop production losses and economic damage under two scenarios of O_3 pollution. Atmos. Environ. 2011; 45(13): 2297–2309. https://doi.org/10.1016/j.atmosenv.2011.01.002.
9. Haagen-Smit AJ. Chemistry and physiology of Los Angeles smog. Ind. Eng. Chem. 1952; 44(6): 1342–1346. https://doi.org/10.1021/ie50510a045.
10. Rai R, Agrawal M. Evaluation of physiology and biochemical responses of two rice (*Oryza sativa* L.) cultivars to ambient air pollution using open top chambers at a rural site in India. Sci. Total Environ. 2008; 407: 679–69. https://doi.org/10.1016/j.scitotenv.2008.09.010.

11. Tiwari S, Rai R, Agrawal M. Annual and seasonal variations in tropospheric ozone concentrations around Varanasi. Int. J. Remote. Sens. 2008; 29: 4499–4514. https://doi.org/10.1080/01431160801961391.
12. Middleton JT, Kendrick JB, Schwalm, HW. Injury to Herbaceous Plants by Smog or Air Pollution. Plant. Disease Reptr. 1950; 34: 245. https:// https://hero.epa.gov/hero/index.cfm/reference/details/reference_id/14944.
13. Richards BL, Middleton JT, Hewitt WB. Air pollution with relation to agronomic crops. V. Oxidant stipple of grape. Agron. J. 1958; 50: 559-561. https://doi.org/10.2134/agronj1958.00021962005000090019x.
14. Cho K, Shibato J, Agrawal GK, Jung YH, Kubo A, Jwa NS, Tamogami S, Satoh K, Kikuchi S, Higashi T, Kimura S, Saji H, Tanaka Y, Iwahashi H, Masuo Y, Rakwal R. Integrated transcriptomics, proteomics, and metabolomics analyses to survey ozone responses in the leaves of rice seedlings. J. Proteome. Res. 2008; 7: 2980–2998. https://doi.org/10.1021/pr800128q.
15. Sarkar A, Agrawal SB. Identification of ozone stress in Indian rice through foliar injury and differential protein profile. Environ. Monit. Assess. 2010; 161: 283–302. https://doi.org/10.1007/s10661-008-0738-z.
16. Frei M, Ashrafuzzaman M, Piepho HP, Herzog E, Begum SN, Islam MM. Evidence for tropospheric ozone effects on rice production in Bangladesh. Sci. Total Environ. 2024; 909: 168560. https://doi.org/10.1016/j.scitotenv.2023.168560.
17. Carnahan JE, Jenner EL, Wat EKW. Prevention of ozone injury to plants by a new protectant chemical. Phytopathol. 1978; 68: 1225–1229.

https://www.apsnet.org/publications/phytopathology/backissues/Documents/1978Articles/Phyto68n08_1225.PDF.

18. Feng Z, Pang J, Nouchi I, Kobayashi K, Yamakawa T, Zhu J. Apoplastic ascorbate contributes to the differential ozone sensitivity in two varieties of winter wheat under fully open-air field conditions. Environ. Pollut. 2010a; 158: 3539–3545. https://doi.org/10.1016/j.envpol.2010.08.019.
19. Manning WJ, Paoletti E, Sandermann H, Ernst D. Ethylenediurea (EDU): a research tool for assessment and verification of the effects of ground level ozone on plants under natural conditions. Environ. Pollut. 2011; 159: 3283-3293. http://dx.doi.org/10.1016/j.envpol.2011.07.005.
20. Paoletti E, Contran N, Manning WJ, Ferrara AM. Use of the antiozonant ethylenediurea (EDU) in Italy: Verification of the effects of ambient ozone on crop plants and trees and investigation of EDU's mode of action. Environ. Pollut. 2009; 157: 1453–1460. https://doi.org/10.1016/j.envpol.2008.09.021.
21. Agathokleous E. Perspectives for elucidating the ethylenediurea (EDU) mode of action for protection against O_3 phytotoxicity. Ecotoxicol. Environ. Saf. 2017; 142: 530–537. https://doi.org/10.1016/j.ecoenv.2017.04.057.
22. Frei M. Breeding of ozone resistant rice: relevance, approaches and challenges. Environ. Pollut. 2015; 197: 144–155. https://doi.org/10.1016/j.envpol.2014.12.011.
23. Ueda Y, Siddique S, Frei M. A novel gene, ozone-responsive apoplastic protein1, enhances cell death in ozone stress in rice. Plant. Physiol. 2015; 169(1): 873–889. https://doi.org/10.1104/pp.15.00956.

24. Frei M, Tanaka JP, Wissuwa M. Genotypic variation in tolerance to elevated ozone in rice: Dissection of distinct genetic factors linked to tolerance mechanisms. J. Exp. Bot. 2008; 59(13): 3741–3752. https://doi.org/10.1093/jxb/ern222.
25. Frei M, Tanaka JP, Chen CP, Wissuwa M. Mechanisms of ozone tolerance in rice: Characterization of two QTLs affecting leaf bronzing by gene expression profiling and biochemical analyses. J. Exp. Bot. 2010; 61(5): 1405–1417. https://doi.org/10.1093/jxb/erq007.
26. Luwe M, Takahama U, Heber U. Role of ascorbate in detoxifying ozone in the apoplast of spinach (Spinacia oleracea L.) leaves. Plant. Physiol. 1993; 101: 969–976. https://doi.org/10.1104/pp.101.3.969.
27. Plöchl M, Lyons T, Ollerenshaw J, Barnes J. Simulating ozone detoxification in the leaf apoplast through the direct reaction with ascorbate. Planta. 2000; 210: 454–467. https://doi.org/10.1007/PL00008153.
28. Feng Z, Wang S, Szantoi Z, Chen S, Wang X. Protection of plants from ambient ozone by applications of ethylenediurea (EDU): a meta-analytic review. Environ. Pollut. 2010b; 158: 3236-3242. http://dx.doi.org/10.1016/j.envpol.2010.07.009.
29. Sanmartin M, Drogoudi PA, Lyons T, Pateraki I, Barnes J, Kanellis AK. Over-expression of ascorbate oxidase in the apoplast of transgenic tobacco results in altered ascorbate and glutathione redox states and increased sensitivity to ozone. Planta. 2003; 216: 918–928. https://doi.org/10.1007/s00425-002-0944-9.
30. Ashrafuzzaman M, Ueda Y, Frei M. Natural sequence variation at the *OsORAP1* locus is a marker for ozone tolerance in Asian rice. Environ. Exp. Bot. 2020; 178 (104153). https://doi.org/10.1016/j.envexpbot.2020.104153

31. Jain M, Nijhawan A, Tyagi AK, Khurana JP. Validation of housekeeping genes as internal control for studying gene expression in rice by quantitative real-time PCR. Biochem. Biophys. Res. Commun. 2006; 345(2): 646-51. https://doi.org/10.1016/j.bbrc.2006.04.140

32. Bates D, Mächler M, Bolker B, Walker S. Fitting Linear Mixed-Effects Models Using lme4. J. Stat. Softw. 2015; 67(1): 1–48. https://doi.org/10.18637/jss.v067.i01.

33. Kuznetsova A, Brockhoff PB, Christensen RHB. lmerTest Package: Tests in Linear Mixed Effects Models. J. Stat. Softw. 2017; 82(13): 1–26. https://doi.org/10.18637/jss.v082.i13

34. Hothorn T, Bretz F, Westfall P. Simultaneous Inference in General Parametric Models. Biom. J. 2008; 50(3): 346–363. https://CRAN.R-project.org/package=multcomp.

35. Lüdecke D, Ben-Shachar MS, Patil I, Waggoner P, Makowski D. Performance: An R Package for Assessment, Comparison and Testing of Statistical Models. J. Open Source Softw. 2021; 6(60): 3139. https://doi.org/10.21105/joss.03139.

36. Ren S, Stroud C, Belair S, Leroyer S, Moran M, Zhang J, Akingunola A, Makar P. Impact of Urban Land Use and Anthropogenic Heat on Air Quality in Urban Environments. In International Technical Meeting on Air Pollution Modelling and its Application; Springer Proceedings in Complexity; Springer: Cham, Switzerland, 2020; 153–158. https://doi.org/10.1007/978-3-030-22055-6_24

37. Ashrafuzzaman M, Lubna FA, Holtkamp F, Manning WJ, Kraska T, Frei M. Diagnosing ozone stress and differential tolerance in rice (*Oryza sativa* L.) with ethylenediurea (EDU). Environ. Pollut. 2017; 230: 339-350. http://dx.doi.org/10.1016/j.envpol.2017.06.055.

38.

39. Fukagawa NK, Ziska LH. Rice: Importance for Global Nutrition. J. Nutr. Sci. Vitaminol. 2019; 65: S2-S3. https://doi.org/10.3177/jnsv.65.S2.

40. Zhang G, Hu Y, Pan X, Cao R, Hu Q, Fu R, Risalat H, Shang B. Effects of increased ozone on rice panicle morphology. iScience. 2023; 26(4): 106471. https://doi.org/10.1016/j.isci.2023.106471.

41. Xu J, Dong X, Zhang T, Liu J, Tao S. 2022. Mitigation of air pollutant impacts on rice yields in China by sector. Environ. Res. Lett. 2022; 17: 054037. DOI 10.1088/1748-9326/ac681c.

42. Anagha KS, Kuttippurath J, Sharma M, Cuesta J, A comprehensive assessment of yield loss in rice due to surface ozone pollution in India during 2005–2020: A great concern for food security. Agric. Syst. 2024; 215:103849. https://doi.org/10.1016/j.agsy.2023.103849.

43. Li S, Yang Y, Wang H, Li P, Li K, Ren L, Wang P, Li B, Mao Y, Liao H. Rapid increase in tropospheric ozone over Southeast Asia attributed to changes in precursor emission source regions and sectors. Atmos. Environ. 2023; 304: 119776. https://doi.org/10.1016/j.atmosenv.2023.119776.

44. Debaje SB, Kakade AD, Jeyakumar SJ. Air pollution effect of O_3 on crop yield in rural India. J. Hazard. Mater. 2010; 183: 773–779. https://doi.org/10.1016/j.jhazmat.2010.07.093.

45. Wang Y, Yang L, Höller M, Zaisheng S, Pariasca-Tanaka J, Wissuwa M, Frei M. Pyramiding of ozone tolerance QTLs *OzT8* and *OzT9* confers improved tolerance to

season-long ozone exposure in rice. Environ. Exp. Bot. 2014; 104: 26–33. https://doi.org/10.1016/j.envexpbot.2014.03.005.

46. Jing LQ, Dombinov V, Shen SB, Wu YZ, Yang LX, Wang YX, Frei M. Physiological and genotype-specific factors associated with grain quality changes in rice exposed to high ozone. Environ. Pollut. 2016; 210: 397–408. https://doi.org/10.1016/j.envpol.2016.01.023.

47. Frei M, Kohno Y, Wissuwa M, Makkar HPS, Becker K. Negative effects of tropospheric ozone on the feed value of rice straw are mitigated by an ozone tolerance QTL. Glob. Change Biol. 2011; 17: 2319–2329. https://doi.org/10.1111/j.1365-2486.2010.02379.x.

48. Kangasjarvi J, Jaspers P, Kollist H. Signalling and cell death in ozone-exposed plants. Plant Cell Environ. 2005; 28: 1021–1036. https://doi.org/10.1111/j.1365-3040.2005.01325.x.

49. Zeng Y, Cao Y, Qiao X, Seyler BC, Tang Y. Air pollution reduction in China: Recent success but great challenge for the future. Sci. Total Environ. 2019; 663: 329–337. https://doi.org/10.1016/j.scitotenv.2019.01.262.

50. Sampedro J, Waldhoff ST, Van de Ven DJ, Pardo G, Van Dingenen R, Arto I, Prado A, Sanz MJ. Future impacts of ozone driven damages on agricultural systems. Atmos. Environ. 2020; 231: 117538. https://doi.org/10.1016/j.atmosenv.2020.117538.

51. Shang B, Fu R, Agathokleous E, Dai L, Zhang G, Wu R, Feng Z. Ethylenediurea offers moderate protection against ozone-induced rice yield loss under high ozone pollution. Sci. Total Environ. 2022; 806(3): 151341. https://doi.org/10.1016/j.scitotenv.2021.151341.

52. Ashrafuzzaman M, Zahidul H, Ali B, Mathew B, Yu P, Hochholdinger F, Neto JBA, McGillen MR, Ensikat HJ, Manning WJ, Frei M. Ethylenediurea (EDU) mitigates the negative effects of ozone in rice: Insights into its mode of action. Plant Cell Environ. 2018; 41:2882–2898. https://doi.org/10.1111/pce.13423.
53. Quijano-Guerta C, Kirk GJD, Portugal AM, Bartolome VI, McLaren GC. Tolerance of rice germplasm to zinc deficiency. Field Crops Res. 2002; 76: 123-130. https://doi.org/10.1016/S0378-4290(02)00034-5.
54. Sohn JK, Lee JJ, Kwon YK, Kim KM. Varietal differences and inheritance of resistance to ozone stress in rice (Oryza sativa L.). SABRAO J. Breed. Genet. 2002; 34: 65-71. https://www.cabidigitallibrary.org/doi/full/10.5555/20033016398.
55. Takehisa H, Ueda T, Fukuta Y, Obara M, Abe T, Yano M, Yamaya T, Kameya T, Higashitani A, Sato T. Epistatic interaction of QTLs controlling leaf bronzing in rice (Oryza sativa L.) grown in a saline paddy field. Breed. Sci. 2006; 56: 287-293. https://doi.org/10.1270/jsbbs.56.287.
56. Surabhi S, Pande V, Pandey V. Ethylenediurea (EDU) mediated protection from ambient ozone-induced oxidative stress in wheat (*Triticum aestivum* L.) under a high CO_2 environment. Atmos. Pollut. Res. 2022; 13(8): 101503. https://doi.org/10.1016/j.apr.2022.101503.
57. Pandey AK, Majumder B, Keski-Saari S, Kontunen-Soppela S, Mishra A, Sahu N, *et al*. Searching for common responsive parameters for ozone tolerance in 18 rice cultivars in India: results from ethylene diurea studies. Sci. Total Environ. 2015; 532: 230–238. https://doi.org/10.1016/j.scitotenv.2015.05.040.

58. Akhtar N, Yamaguchi M, Inada H, Hoshino D, Kondo T, Fukami M, *et al*. Effects of ozone on growth, yield and leaf gas exchange rates of four Bangladeshi cultivars of rice (*Oryza sativa* L.). Environ. Pollut. 2010; 158: 2970–2976. https://doi.org/10.1016/j.envpol.2010.05.026.

59. Gupta SK, Sharma M, Majumder B, Maurya VK, Lohani M, Deeba F, *et al.* Impact of Ethylenediurea (EDU) on growth, yield and proteome of two winter wheat varieties under high ambient ozone phytotoxicity. Chemosphere. 2018; 196: 161–173. https://doi.org/10.1016/j.chemosphere.2017.12.150.

60. Rai R, Agrawal M, Choudhary KK, Agrawal SB, Emberson L, Büker P. Application of ethylene diurea (EDU) in assessing the response of a tropical soybean cultivar to ambient O3: Nitrogen metabolism, antioxidants, reproductive development and yield. Ecotoxicol. Environ. Saf. 2015; 112: 29–38. https://doi.org/10.1016/j.ecoenv.2014.10.031.

61. Begum H, Alam MS, Feng Y, Koua P, Ashrafuzzaman M, Shrestha A, *et al.* Genetic dissection of bread wheat diversity and identification of adaptive loci in response to elevated tropospheric ozone. Plant Cell Environ. 2020; 43: 2650–2665. https://doi.org/10.1111/pce.13864.

62. Meroni M, Panigada C, Rossini M, Picchi V, Cogliati S, Colombo R. Using optical remote sensing techniques to track the development of ozone-induced stress. Environ. Pollut. 2009; 157: 1413–1420. https://doi.org/10.1016/j.envpol.2008.09.018.

Supporting information

S1 Fig. The DNA profile of 20 rice genotypes using primer KAS_1_2, which showing the band on Kasalath-type (specific) genotypes

S2 Fig. Photographs of different rice varieties under ambient ozone stress conditions with and without the application of EDU during the irrigated (Boro) season at Mymensingh, Bangladesh (2022). [Shown are images of six rice variety with two treatments: (a) BRRI hybrid dhan3 without EDU (Control), (b) BRRI hybrid dhan3 with EDU, (c) BRRI dhan28 without EDU (Control), (d) BRRI dhan28 with EDU, (e) BRRI dhan29 without EDU (Control), and (f) BRRI dhan29 with EDU. Plants were exposed to ambient ozone stress, and EDU (ethylene diurea) was applied to assess its protective effects against ozone stress]

S1 File. Ozone concentration (ppb) data during the growing period

S2 File. Yield and yield contributing characters data of Kasalath and Nipponbare type varieties at ambient ozone stress with EDU and without EDU (control) application

S3 File. Reflectance index data of Kasalath and Nipponbare type varieties at ambient ozone stress with EDU and without EDU (control) application

S4 File. Expression analysis data from delta-delta CT quantification method

Figure 1

Click here to access/download;Figure;Figure_1.tif

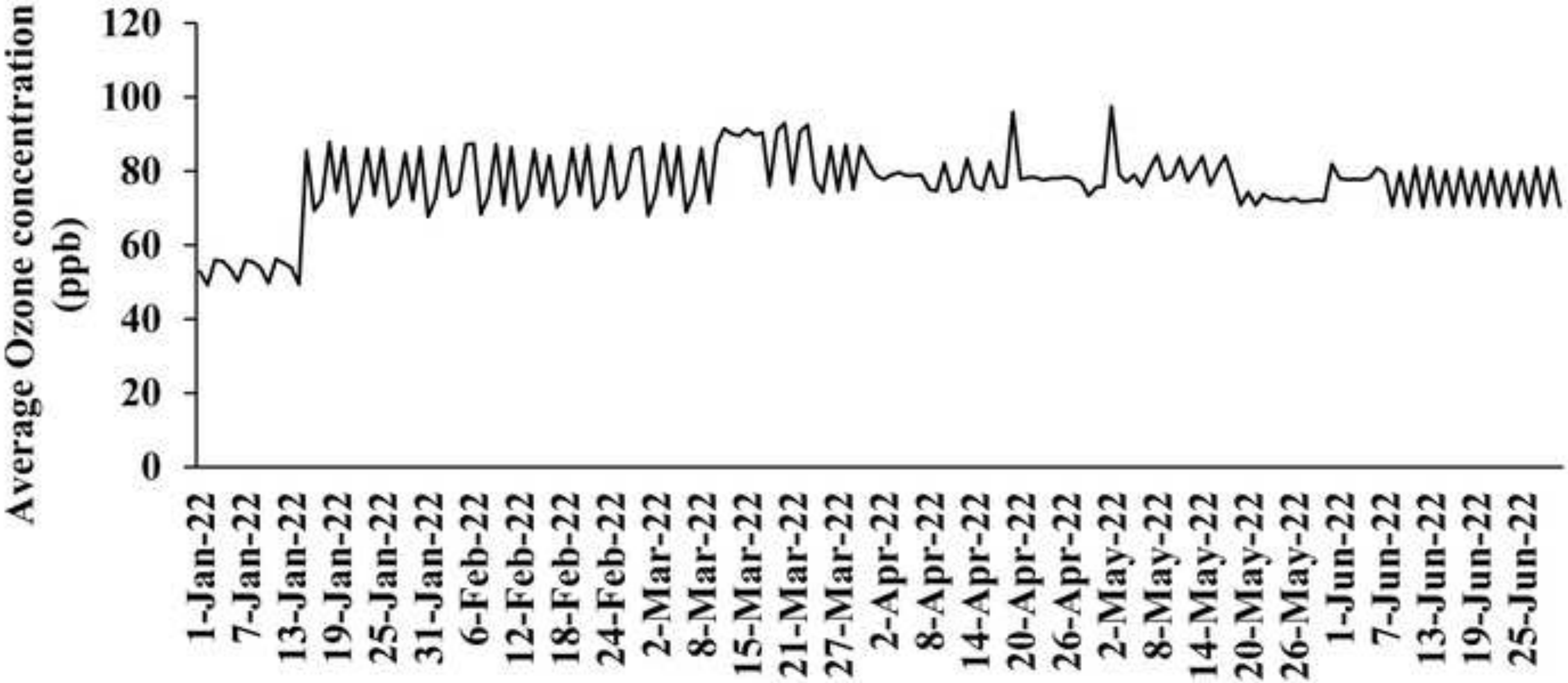

Figure 2

Click here to access/download;Figure;Figure_2.tif

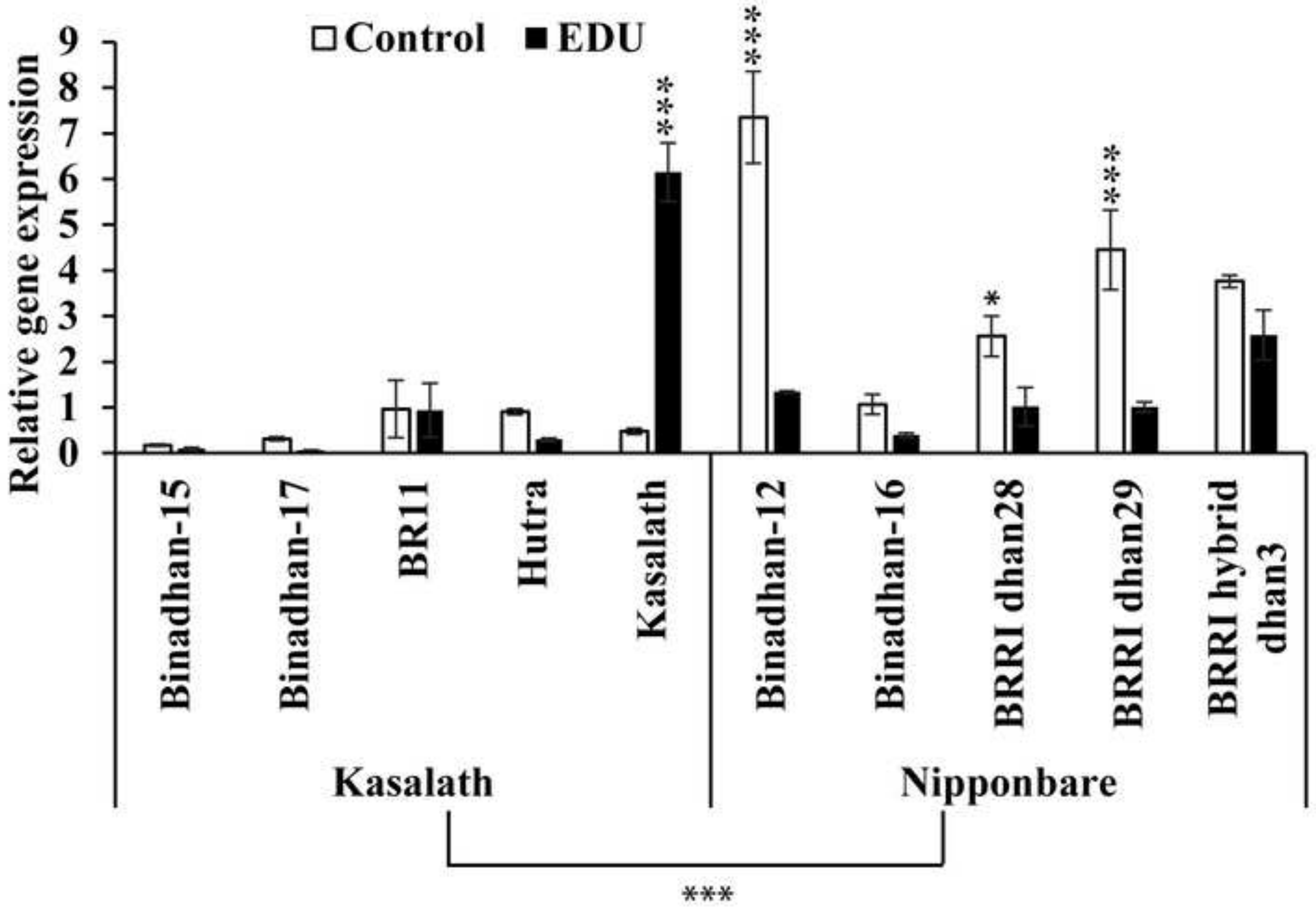

Figure 3

Click here to access/download;Figure;Figure_3.tif

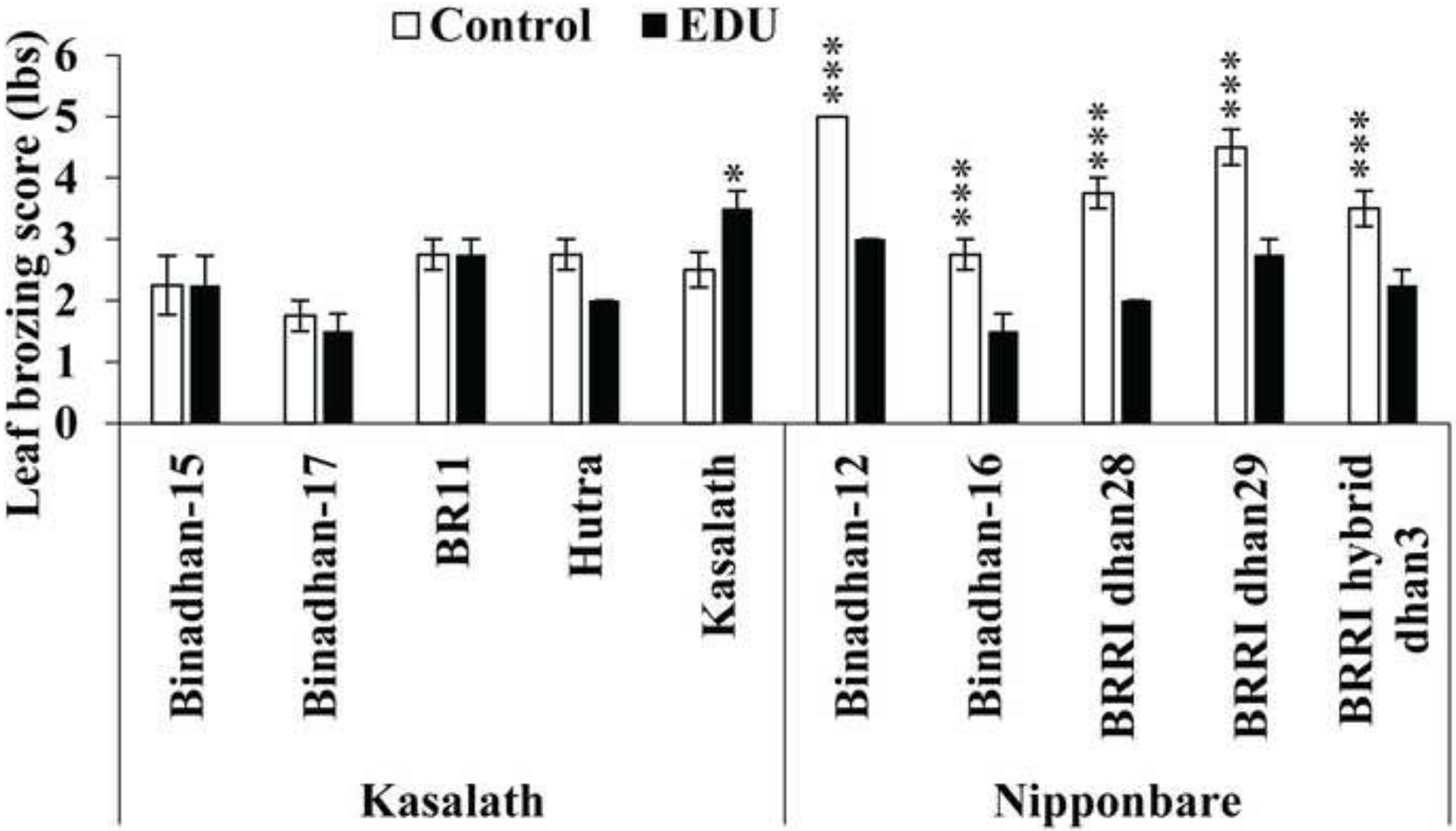

Figure 4

Click here to access/download;Figure;Figure_4.tif

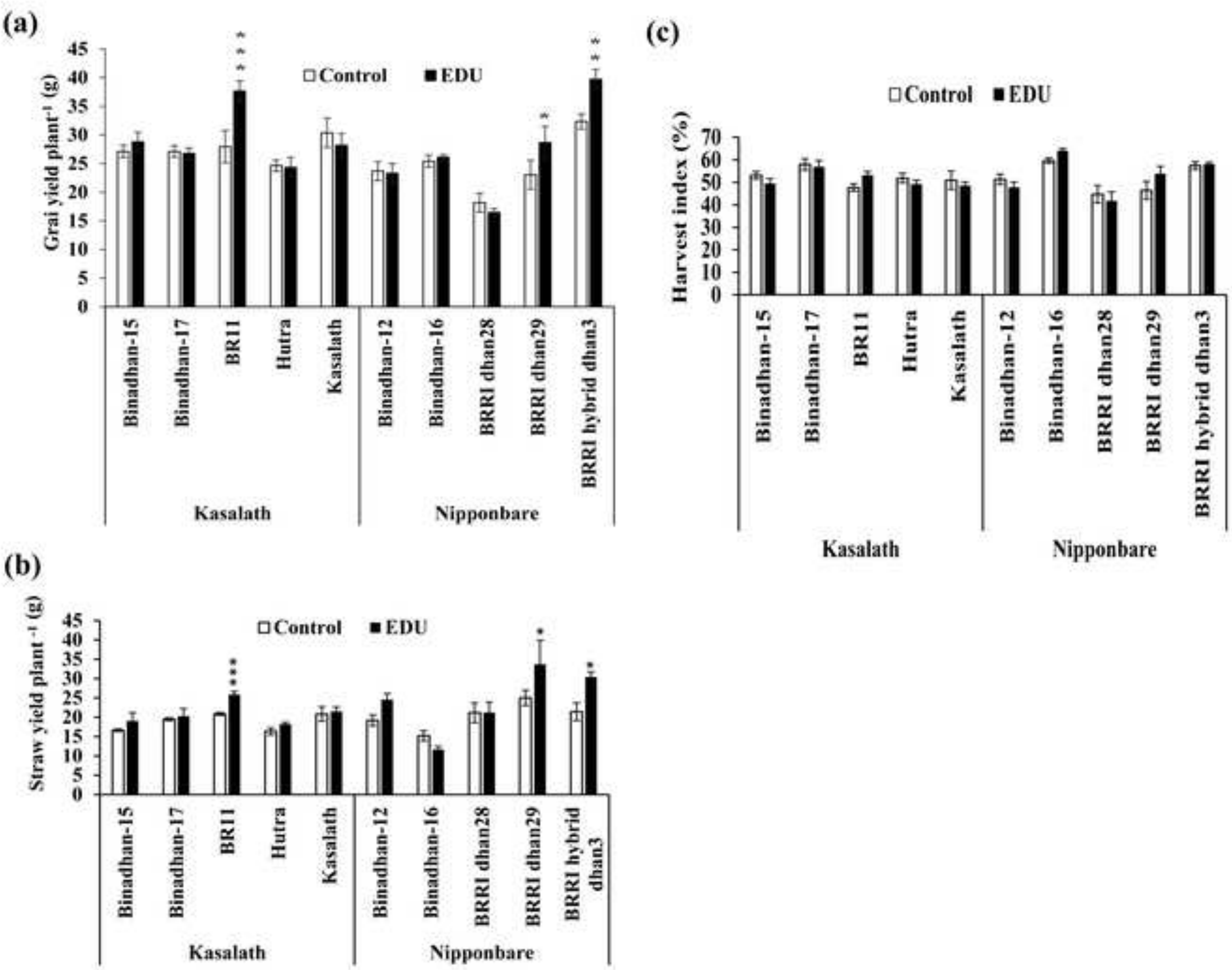

Figure 5

Click here to access/download;Figure;Figure_5.tif

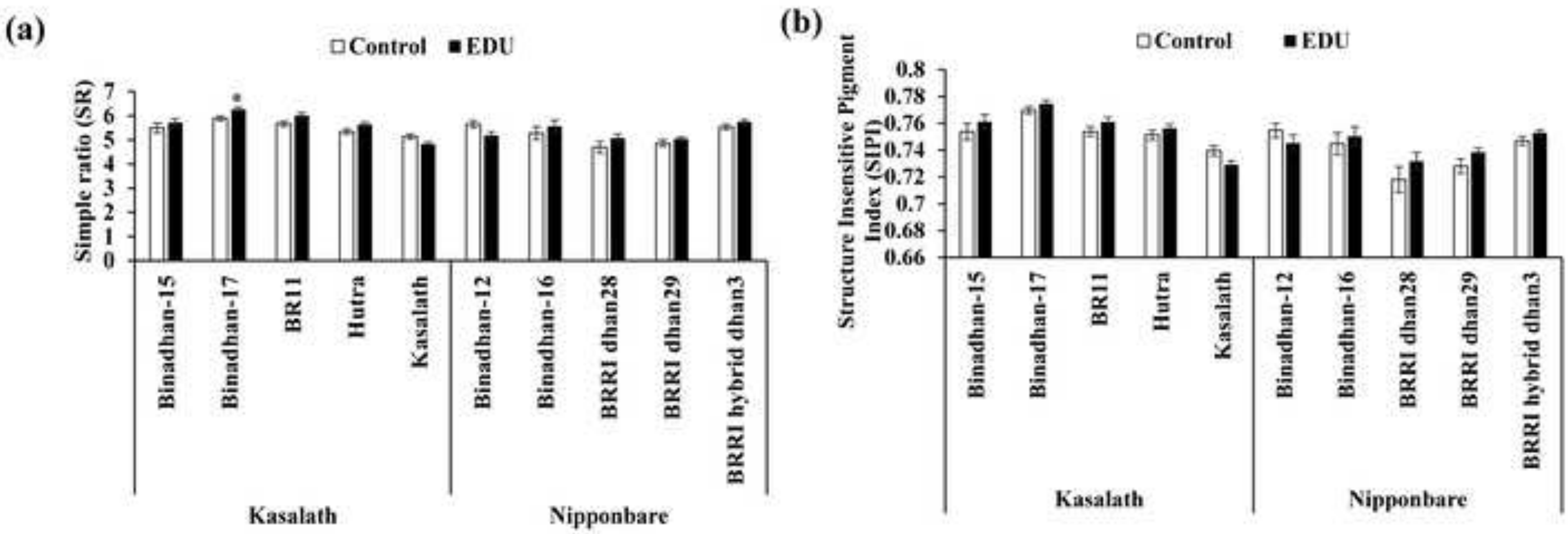

S1 Fig

Click here to access/download
Supporting Information
S1 Fig.tif

S1 File

Click here to access/download
Supporting Information
S1 File.xlsx

S1 raw image

Click here to access/download
Supporting Information
S1_raw_images.pdf

S2 Fig

Click here to access/download
Supporting Information
S2 Fig.tif

S2 File

Click here to access/download
Supporting Information
S2 File.xlsx

S3 File

Click here to access/download
Supporting Information
S3 File.xlsx

S4 File

Click here to access/download
Supporting Information
S4 File.xlsx

Expression of *OsORAP1* allele in rice (*Oryza sativa* L.) at ambient ozone stress in relation to ethylenediurea (EDU) application

Rigyan Gupta[1,2], Shamsul H. Prodhan[1], Md. Nazmul Hasan[1], Shamsun Nahar Begum[2], Mohammad Hasanuzzaman Rani[2], Mirza Mofazzal Islam[2], Md Ashrafuzzaman[1*]

[1]Department of Genetic Engineering & Biotechnology (GEB), School of Life Sciences, Shahjalal University of Science and Technology (SUST), Sylhet, Bangladesh

[2]Plant Breeding Division, Bangladesh Institute of Nuclear Agriculture (BINA), Mymensingh, Bangladesh

*Corresponding author

E-mail: azamanbt@gmail.com

Formatted: Font: Italic

Abstract

Rising tropospheric ozone concentrations pose a significant threat to crop production in many Asian countries like Bangladesh, necessitating the development of ozone-tolerant rice (*Oryza sativa* L.) varieties. This study investigates the response of *OsORAP1* allele in different rice varieties, particularly Kasalath-type and Nipponbare-type, to Ethylenediurea (EDU) treatment under ambient ozone stress. The experiment, conducted during the 2022 rainfed season, involved growing of 20 rice varieties under a split-plot design with and without EDU treatment, followed by genomic DNA collection at the vegetative stage to differentiate Kasalath-types and Nipponbare-types, and RNA extraction from 10 selected varieties at the flowering stage for gene expression analysis. The research revealed distinct genetic responses between the two types, with Nipponbare-type varieties showing significant increases in *OsORAP1* expression and more leaf bronzing score (LBS) following EDU treatment. In contrast, Kasalath-type varieties exhibited less pronounced responses due to their inherent tolerance to ozone stress. The effects of EDU on with Kasalath-type varieties responding notably in parameters such as ineffective tillers and straw yield, while Nipponbare-types showed significant changes in unfilled grains and grain yield. Additionally, differences were observed in how EDU treatment influenced reflectance indices parameters, indicating variability in how these varieties mitigate ozone stress. The study underscores the complex interaction between genetic factors, environmental conditions, and treatment in shaping the response of rice varieties to EDU, and

Formatted: Indent: First line: 0.5"

Formatted: Font: Italic

suggests future research to further explore the genetic mechanisms, particularly the role of *OsORAP1*, that contribute to these differential responses.

Key words: Ozone, Stress, Ethylenediurea (EDU), *OsORAP1*, Rice, Food security, Global change

1. Introduction

The presence of tropospheric ozone (O_3) in the atmosphere is a major threat to crop production globally. Its high phytotoxicity and occurrence above agrarian regions make it a crucial atmospheric contaminant [1, 2, 3]. The formation of O_3 is a result of the photochemical reactions of precursor gases that are mainly emitted from anthropogenic sources such as nitrogen oxides, carbon monoxide, volatile organic compounds, and methane [4, 5, 6]. The adverse effects of ambient O_3 can lead to estimated yield losses of 4% globally and 10% regionally for rice [2, 7] and 4-17% for wheat, 10-14% for soybean, and 3-6% for maize, resulting in a projected annual economic loss of US$ 12-21 billion based on global crop models [8].

Formatted: Indent: First line: 0.5"

The harmful effects of O_3 on vegetation were first identified in North America during the 1950s and later observed in Europe and Japan in the 1970s [5, 9]. Ground-level O_3 concentrations have significantly increased in urban areas and are even higher in rural areas than in cities [10, 11]. The first recorded incident of photochemical smog and O_3 damage to vegetation was reported by Middleton *et al.* [12], while Richards *et al.* [13] found O_3 as a phytotoxic constituent of photochemical smog while investigating leaf injury in grape vines (*Vitis vinifera*) in Southern California.

Upon entering plant stomata, O_3 dissolves in the apoplast and generates different reactive oxygen species (ROS). These ROS can change cellular functions that result in premature senescence, cell death, and the up- or down-regulation of specific genes [14, 15]. Rice (*Oryza sativa* L.) is a staple crop in Asia but is severely affected by tropospheric O_3, particularly in South and East Asia. Since its cropping season overlaps with peak O_3 concentrations, breeding O_3-tolerant rice varieties is necessary to ensure global food supply [16] as the demand for food is projected to double by 2050. The chemical compound Ethylenediurea (EDU; N- [2-(2-oxo-1-imidazolidinyl) ethyl]-N′-phenylurea) was initially introduced by Carnahan, Jenner, and Wat in 1978 [17] and has been utilized for a considerable time to assess the effects of O_3 on plants [18, 19, 20]. EDU is conveniently sprayed on leaves, works as an antiozonant, and provides a "control" without O_3 damage. The mode of action of EDU against O_3 is still unclear. It can be used to screen plants for O_3 sensitivity under ambient conditions, verify foliar O_3 symptoms in the field, and thus suppress O_3 injury in plants [19, 21].

Previous research has identified genetic factors associated with O_3 tolerance in rice, suggesting that this trait is controlled by multiple medium-effect loci rather than a single large-effect locus [22, 23]. In a study by Frei *et al.* [24] using a mapping population derived from the cultivars Nipponbare (susceptible) and Kasalath (tolerant), certain O_3-related quantitative trait loci (QTLs) were identified. One of the identified QTLs, OzT9, was associated with visible leaf symptom formation in response to O_3 stress, where the allele from the tolerant cultivar Kasalath conferred tolerance. The effectiveness of OzT9 was supported by SL41, a chromosomal segment substitution line carrying a chromosomal introgression from Kasalath at the OzT9 locus in the Nipponbare background, which showed fewer visible symptoms than Nipponbare [24]. Further transcriptomic analysis revealed differential gene expression profiles between Nipponbare and

SL41A under O_3 stress [25]. Physiologically, apoplastic ascorbate (ASA) is a line of defense against O_3 entering the plant through stomata. Several studies, including Luwe *et al.* [26], Plöchl *et al.* [27], and Feng *et al.* [28], support this connection between the annotation of candidate genes and O_3 stress tolerance. Moreover, a previous study showed that an AO-overexpressing tobacco plant affects O_3 tolerance adversely due to altered apoplastic reduced ASA content and ASA redox conditions [29]. This transformative evidence suggests that the expression of the putative AO gene is also associated with O_3 stress tolerance in rice and the formation of significant symptoms.

According to a study by Frei *et al.* [25], the expression of the *OsORAP1* (OZONE RESPONSIVE APOPLASTIC PROTEIN1) gene in response to O_3 stress was significantly lower in the O_3-tolerant SL41 variety compared to the cv. Nipponbare. The gene was located near a QTL and showed differential regulation between a sensitive parent and a tolerant introgression line. Additional analysis revealed that the promoter region of *OsORAP1* had widespread polymorphisms, with the OzT9 donor variety responsible for this variation between the O_3-susceptible cv. Nipponbare and the O_3-tolerant cv. Kasalath. *OsORAP1* encodes an apoplastic protein that is involved in cell death processes but has no ascorbate oxidase activity. The lower or no expression of ~~absence of~~ *OsORAP1* enhanced O_3 tolerance in rice, leading to its designation as OZONE RESPONSIVE APOPLASTIC PROTEIN1. A previous study utilized a transgenic T-DNA insertion line to suppress *OsORAP1* expression and confer O_3 tolerance. However, it is unclear whether this suppression can also be achieved naturally through polymorphism. Generally, the Nipponbare type allele, carried by a group of rice varieties, showed a significantly higher level of foliar symptom formation, lipid peroxidation, and biomass loss when exposed to elevated O_3 in a controlled setting [25]. Furthermore, the Nipponbare types

exhibited significantly higher induction of *OsORAP1* expression in response to O_3 stress, suggesting that *OsORAP1* plays a crucial role in regulating O_3 response and susceptibility or tolerance in rice.

Therefore, the aim of this study was to investigate the impact of O_3 stress on rice and to assess the potential of EDU as a bio-monitoring tool for O_3 in highly affected developing countries such as Bangladesh. Since conventional O_3 experimental facilities like open top chamber (OTC) and free-air concentration enrichment (FACE) O_3 are lacking in these countries, this study aimed to evaluate the expression of *OsORAP1* gene and its correlation with O_3 tolerance when the plants are continuously exposed to an average of 70 ppb or more ambient O_3 applying EDU by collecting the leaf samples during the flowering stage to assess gene expression. The study was conducted based on 10 Bangladeshi rice varieties during their flowering stage with and without the application of EDU (300 ppm, once a week after transplanting until harvesting).

2. Materials and Methods

2.1 Experimental site and plant materials

The experiment was set in Bangladesh Institute of Nuclear Agriculture (BINA) HQs, Mymensingh farm (24° 43' N latitude and 90° 25' E longitude), Bangladesh which is situated in Old Brahmaputra Floodplain (AEZ-23) at rainfed (boro) season of 2022 (which is denoted as dry season usually depend on irrigated water and the crops transplanted from December to early February and harvested between April and June). A total of twenty high yielding popular (inbred and hybrid) and local diversified *indica and Aus* rice genotypes were used in this experiment,

Formatted: Indent: First line: 0.5"

Formatted: Font: Italic

and 10 (Binadhan-15, Binadhan-17, BR11, Hutra, Kasalath, Binadhan-12, Binadhan-16, BRRI dhan28, BRRI dhan29, and BRRI hybrid dhan3) representative were selected for gene expression analysis with details provided in table 1.

2.2 Growing of varieties ~~and~~, design of the experiments and measurement of data

The seeds of twenty varieties were wrapped up in water container for 24 hours. After that, the seeds were removed from water and kept in gunny bags and leave in dark room. It was required about 3-5 days to germinate the all seed samples. Then, the germinated seed were sown in nursery beds with definite spacing and well tagged with a good layout. The experimental fields were prepared by ploughing and laddering with a tractor. By removing all the stubbles and weeds the field was then prepared. Then thirty-five day old seedlings were detached from the nursery bed with healthy root system. The experiments were than laid out as split-plot designs with four replicates arranged in blocks, including EDU treatment as the main plot (i.e. with and without EDU), and genotype as the subplot. Each subplot was 4 m^2 (2 × 2 m) size of which a central one m^2 area were used for yield estimation at harvest and the border plants may be used for destructive sampling during the growing season. After that, the seedling were transplanted in the main field at the rate one seedling per hill with a spacing 20 cm row to row distance and 15 cm hill to hill distance. Considering two treatments and four replicates, each experimental field was approximate size of 1050 m^2 (1000 m^2 experimental area plus dikes, bunds, irrigation channels, etc.). Urea, triple super phosphate, muriate of potash, gypsum, zinc sulphate and boron (the main source of N, P, K, S, Zn and B respectively) with a dose @ 270 Kg, 115 Kg, 150 Kg, 75 Kg, 6 Kg and 7 Kg, respectively were applied in the experimental field. During the final land

Formatted: Indent: First line: 0.5"

preparation, Carbofuran-5G @ 10 Kg/ha (a granular insecticide) and all fertilizers excluding urea and zinc sulphate were applied; one-third of the urea dose was applied before the full dose of zinc sulphate was applied 12 days after transplanting. Again, urea were used in two split doses at 30 and 45 days after transplanting. For the reductions of insect and other pest infestation the pesticides were applied as when necessary. Three times of weeding were done at 15, 30 and 45 DAT.

Before each measurement, five randomly selected plants from each subplot were tagged. After harvesting, data were collected from a 1 m^2 area in each subplot, along with the five selected plants. The measured parameters included plant height (cm), total number of tillers per plant, number of effective and non-effective tillers per plant, panicle length (cm), filled grain count per plant, filled grain weight per plant, unfilled grain count per plant, unfilled grain weight per plant, grain yield, straw yield, and thousand-kernel weight. The harvest index (%) was measured by using the following formula:

Harvest index (%) = (Grain yield)/(Total biomass) × 100

A leaf bronzing score (LBS) ranging from 0 to 10 was used to assess ozone-induced leaf damage, following the method described by Ueda et al. [23]. A score of 0 represented no visible symptoms on any leaves, while a score of 10 indicated severe damage affecting the entire plant due to ozone stress.

Stomatal conductance was assessed using a leaf porometer (Model SC1, Decagon Devices, Pullman, WA) on the youngest fully expanded leaves of a selected plant per subplot. Measurements were taken on sunny days between 9:30 AM and 2:00 PM. To evaluate ozone stress, spectral reflectance was recorded with a handheld spectroradiometer (Polypen RP410,

Formatted: Superscript

PRI, Czech Republic). The normalized difference vegetation index (NDVI) was calculated using the formula: $(R_{780} - R_{630}) / (R_{780} + R_{630})$. Similarly, the Lichtenthaler Index 2 (Lic2) was determined as the ratio of R_{440} to R_{690}, Simple ratio index (SR) = R_{NIR}/R_{RED}, Photochemical reflectance index (PRI) = $(R_{531} - R_{570})/(R_{531} + R_{570})$, Anthocyanin reflectance index (ARI) = $(R_{550})^{-1} - (R_{700})^{-1}$. Here, in the vegetation indices, R refers to reflectance and subscript indicates the wave bands in nanometers.

2.3 Plant material collection for extraction of Genomic DNA and analysis

Young, vigorously growing fresh leaf samples from the 20 rice genotypes were collected from the seedlings at vegetative stage (85 days old seedlings) to extract genomic DNA. Initially, healthy portion of the youngest leaves of the seedlings were cut apart with sterilized scissors and washed in distilled water and ethanol and dried on fresh tissue paper to remove spore of microorganisms and any other source of foreign DNA. The collected leaf samples were then put into polythene bags and kept on ice in an icebox. Then, the polythene bags were wrapped in aluminium foil and stored at -80^{0}C freezer.

Genomic DNA of the selected rice genotypes were extracted from leaf samples using a DNeasy Plant Mini Kit (Qiagen, Düsseldorf, Germany). For confirmation of genomic DNA, agarose gel electrophoresis were done by visualizing the DNA band in the Gel Doc system (Biometra, Japan). For the determination of quantity and purity the extracted samples were analyzed with Nanodrop 2000c (Thermo Fisher Scientific Inc., Wilmington, DE, USA). Then the DNA concentrations were adjusted to 25 ng/ µl. Then, PCR reactions were performed on each DNA sample in a 10µl reaction mix containing the following reagents: 1µl Ampli Taq

Formatted: Subscript
Formatted: Subscript
Formatted: Subscript
Formatted: Subscript
Formatted: Subscript
Formatted: Subscript
Formatted: Subscript
Formatted: Subscript
Formatted: Subscript
Formatted: Subscript
Formatted: Subscript
Formatted: Subscript
Formatted: Subscript
Formatted: Superscript
Formatted: Subscript
Formatted: Superscript
Formatted: Indent: First line: 0.5"

polymerase buffer (10X), 2.5µl Primer (10µM), 1µl dNTPs (250µM), 0.2µl Ampli Taq DNA polymerase, 2µl Genomic DNA (25ng/µl) and 3.3µl Sterile deionized water. DNA amplification were performed in an oil-free thermal cycler. The reaction mix were preheated at 94^0C for 5 minutes followed by 35 cycles of 1 min denaturation at 94^0C, 1 min annealing at 54^0C and, elongation or extension at 72^0C for 2 minutes. After the last cycle, a final step for 7 minutes at 72^0C were allowed to complete the extension of all amplified fragments. After completion of the cycling program, reactions were held at 4^0C. The primer KAS_1_2 sequence that were used for the initial screening is 5´-GCCTTCCTCCTTGTGGTCG-3´ (forward) and 5´-AGGGAACGTCCCACTGTTG-3´ (reverse) for the Kasalath-type *OsORAP1* allele (annealing temperature 57 °C).

Again, agarose gel electrophoresis were done by ethidium bromide staining for visualizing the band (Kasalath-type) in the Gel Doc system (Biometra, Japan). Among the Ttwenty genotypes (genotypes with Kasalath-type *OsORAP1* allele) were selected the selection was based on the presence and absence of a PCR band [30] for further analyses (Table 1). Based on PCR bands we classified the varieties into two distinct clades: Kasalath-types and Nipponbare-types and found that among the 20 rice varieties, 8 varieties showed PCR bands of were Kasalath-type *OsORAP1* allele and the remaining 12 varieties which were not show PCR bands of were Nipponbare-type *OsORAP1* allele [30].

Table 1: Brief description, collection and/or origin of the rice varieties investigated in this experiment carrying different alleles at the *OsORAP1* locus using primer KAS_1_2 [30]

Sl. No.	Variety name	Short description	Collection and/or origin	Subspecies	*OsORAP1* allele

1	Iratom-24	Early maturing high-yielding variety (HYV)	BINA / Bangladesh	*Indica*	Nipponbare-type
2	Binadhan-6	HYV	BINA / Bangladesh	*Indica*	Kasalath-type
3	Binadhan-10	Salt tolerant HYV	BINA / Bangladesh	*Indica*	Nipponbare-type
4	Binadhan-11	Submergence tolerant	BINA / Bangladesh	*Indica*	Nipponbare-type
5	Binadhan-12	Submergence tolerant (fine grain)	BINA / Bangladesh	*Indica*	Nipponbare-type
6	Binadhan-14	Late sowing potential HYV	BINA / Bangladesh	*Indica*	Kasalath-type
7	Binadhan-15	Premium quality	BINA / Bangladesh	*Indica*	Kasalath-type
8	Binadhan-16	Short duration HYV	BINA / Bangladesh	*Indica*	Nipponbare-type
9	Binadhan-17	HYV	BINA / Bangladesh	*Indica*	Kasalath-type
10	BR11	Widely grown HYV	BRRI / Bangladesh	*Indica*	Kasalath-type
11	BRRI dhan28	Early maturing	BRRI /	*Indica*	Nipponbare-

		HYV	Bangladesh		type
12	BRRI dhan29	HYV	BRRI / Bangladesh	*Indica*	Nipponbare-type
13	BRRI dhan88	Early maturing HYV	BRRI / Bangladesh	*Indica*	Nipponbare-type
14	BRRI hybrid dhan1	High-yielding hybrid variety	BRRI / Bangladesh	*Indica*	Nipponbare-type
15	Hutra	Landrace	Chapainawabganj/ Bangladesh	*Aus*	Kasalath-type
16	Tepiboro	Landrace	Chapainawabganj/ Bangladesh	*Aus*	Kasalath-type
17	BRRI hybrid dhan3	High-yielding hybrid variety	BRRI / Bangladesh	*Indica*	Nipponbare-type
18	IR64	International mega-variety	BRRI / IRRI, Philippines	*Indica*	Nipponbare-type
19	Kasalath	Landrace	BRRI / Bangladesh	*Aus*	Kasalath-type
20	BRRI dhan58	HYV	BRRI / Bangladesh	*Indica*	Nipponbare-type

Note: BINA = Bangladesh Institute of Nuclear Agriculture, BRRI = Bangladesh Rice Research Institute and IRRI = International Rice Research Institute

2.4 Ozone treatment and monitoring

After transplanting, the two treatments viz. with EDU (Ethylenediurea) and without EDU (control) were applied and plants were continuously exposed to natural field conditions of BINA HQs farm, Mymensingh, Bangladesh where the ambient ozone concentration was more than 70 ppb, quantified by a well handheld ozone device (series 500; Aeroqual Ltd. Auckland, New Zealand) at every day after transplanting. The chemical EDU at the concentration of 300 ppm once a week were applied as a foliar spray in the morning (9 am to 12 pm) to mitigate the ozone effects [16]. The controls plant were sprayed with similar of water which was needed for spraying EDU.

Formatted: Indent: First line: 0.5"

2.5 Collection of leaf samples for RNA isolation and gene expression analyses

At the flowering stage (115 days old) between 10 a.m. and 12 p.m., the leaf samples of the selected rice varieties viz. 5 Kasalath-types varieties (Binadhan-15, Binadhan-17, BR11, Hutra, and Kasalath) and 5 Nipponbare–type (Binadhan-12, Binadhan-16, BRRI dhan28, BRRI dhan29, and BRRI hybrid dhan3) were collected. Two randomly selected plants of each genotype from each treatment plot were collected and leaves were pooled for one representative sample. Then the samples were flash-frozen in liquid nitrogen and stored at −80 °C for RNA extraction.

Formatted: Indent: First line: 0.5"

Again, each genotype among the 10 genotypes was extracted from 3 replicates (of 4) of each treatment, yielding a total of 6 samples. In the next step, whole leave samples were ground in liquid nitrogen to a fine powder, and total RNA was extracted with the Promega RNA Isolation Kit containing RQ1 DNase (Promega). In order to determine the quantities and purity of the extracted samples, spectrophotometers NanoDropTM 2000/2000c (Thermo Fisher

Scientific Inc., Wilmington, DE, USA) was used (Thermo Fisher Scientific Inc., Wilmington, DE, USA). Reverse transcription of 300 ng of total RNA was performed using TAKARA PrimeScriptTM 1st strand cDNA Synthesis Kit (Cat. # 6110A). In accordance with the manufacturer's instructions, quantitative PCR was performed with TAKARA TB Green Premix Ex Taq (Tli RNase H Plus) (Cat. # RR420A). In the reaction mixture, there were 7.5µl of TB Green Premix Ex Taq (Tli RNaseH Plus) (2X), 0.4µl of each gene-specific primer (10 µM), 0.2µl of ROX Reference Dye (50X), 4.5µl of nuclease-free water, and 2µl of cDNA Template (15 ng) sample. The following thermal conditions prevailed: denaturation at 95 °C for 10 min, 40 cycles of 15 s of denaturation at 95 °C, and 1 min of annealing and extension at 60 °C. After each reaction, melting curves were analyzed to verify primer specificity. In this study, the housekeeping gene named 18S rRNA (AK059783) was amplified using 5´-CTACGTCCCTGCCCTTTGTACA-3´ (forward) and 5´-ACACTTCACCGGACCATTCAA-3´ (reverse) cast-off as an endogenous reference [31]. A delta-delta (Δ-Δ) CT quantification method was applied to quantify target genes according to Frei *et al.* [25]. Based on a cDNA dilution series, the efficiency of amplification was more than 90% for the primer pair. Analytical duplicates were used for gene expression analyses. An expression analysis of *OsORAP1* was conducted using the primer pair's 5´-CATCGAGGCGCACTTCTT-3´ (forward) and 5´-CGAACGGCTAGCTTCTGGT-3´ (reverse).

2.5 Statistical analysis

For analysis of variance the experimental data were subjected to mixed model analysis using the package lme4 in R [32] with Satterthwaite's method where~~.~~ EDU treatment, variety, and their interaction were set as fixed effects. Pairwise comparisons of individual varieties with

Formatted: Indent: First line: 0.5"

and without EDU treatment were obtained using the procedure emmeans in R and adjusted using the Tukey's method. The total analysis was conducted in R 4.2.1 (R Core Team 2022) with R packages lmerTest [33], multcomp [34] and performance [35].

3. Results

3.1 Ozone concentrations levels in study site

During whole growing period the average ozone concentrations were more than 70 ppb (76.49 ppb) where the threshold level of ozone for crop production is 40 ppb [3~~7~~6]. Daily average ozone concentration were given in Fig 1 where it was found that the ozone concentrations was more than the threshold level which drastically affects the production of rice in Mymensingh, Bangladesh.

Formatted: Indent: First line: 0.5"

Fig 1. Daily average ozone concentration in experimental location (Myemensingh) in the *Boro* rice growing seasons 2022.

3.2. Varietal differences in the responsiveness of *OsORAP1* expression to EDU treatment in rice

This study analyzed the *OsORAP1* locus among different rice varieties and revealed two distinct clades of varieties: the Nipponbare-type and the Kasalath-type allele. To investigate the effect of EDU on mRNA expression, 10 rice varieties were grown and exposed to ambient O_3 stress in a field condition where the O_3 concentration was higher than 70 ppb. The Kasalath and Nipponbare types both showed highly significant (P <0.001) *OsORAP1* expression in EDU

Formatted: Indent: First line: 0.5"

treatment, variety and, EDU treatment and varietal interaction which indicate the EDU have effect on *OsORAP1* expression. (Table 2).

Formatted: Font: Italic

Table 2. Analysis of variance (ANOVA) of different traits (Molecular, Agronomic and physiological) of ten selected rice varieties grown in ozone-polluted location of Mymensingh, Bangladesh in rainfed'22 season with the application of EDU or without application of EDU (control)

Traits	ANOVA						Treatment Means	
	EDU-Treatment		Variety		Interaction		Control	EDU
	Pr(>F)	F values	Pr(>F)	F values	Pr(>F)	F values		
**Kasalath-type**								
Molecular								
OsORAP1 Expression	<0.000***	19.483	<0.000***	30.415	<0.000***	31.076	0.567	1.508
Agronomic								
Leaf bronzing score (lbs)	1.000	0.000	<0.000***	6.565	0.074	2.411	2.400	2.400
No. of effective tiller plant^{-1}	0.081	3.272	0.032*	3.038	0.157	1.790	9.170	9.950
No. of ineffective tiller plant^{-1}	0.023*	5.845	0.002**	5.681	0.824	0.376	0.800	0.570
No. of filled grain plant^{-1}	<0.000***	17.871	<0.000***	42.807	0.723	0.518	1362.000	1522.490
No. of unfilled grain plant^{-1}	0.366	0.847	<0.000***	22.610	0.250	1.433	537.120	515.580
Thousand kernel weight (TKW)	0.783	0.077	<0.000***	1536.219	0.979	0.107	24.076	24.063
Grain yield plant^{-1}	0.144	2.253	0.001**	5.902	0.020*	3.435	274.333	291.318
Straw yield plant^{-1}	<0.000***	7.729	<0.000***	8.136	0.404	1.038	18.824	20.987
Harvest index (%)	0.554	0.358	0.015*	3.661	0.313	1.244	52.316	51.433
Physiological								
NDVI	0.052	3.868	<0.000***	20.637	0.044	2.539	0.690	0.698
SR	0.028*	4.950	<0.000***	20.622	0.053	2.411	5.503	5.683
SIPI	0.245	1.368	<0.000***	24.909	0.111	1.928	0.754	0.756
Lic2	0.010*	6.803	<0.000***	10.389	0.376	1.068	0.675	0.690
PRI	0.432	0.620	<0.000***	8.533	0.250	1.367	0.055	0.056
**Nipponbare-type**								

Molecular								
OsORAP1 Expression	<0.000***	65.595	<0.000***	15.000	<0.000***	9.512	3.839	1.270
Agronomic								
Leaf bronzing score (lbs)	<0.000***	128.000	<0.000***	21.375	0.363	1.125	3.900	2.300
No. of effective tiller plant^{-1}	0.670	0.185	0.035*	2.957	<0.000***	8.999	10.270	10.090
No. of ineffective tiller plant^{-1}	0.364	0.850	0.049*	2.701	0.935	0.202	0.870	0.710
No. of filled grain plant^{-1}	0.172	1.962	<0.000***	122.829	<0.000***	4.429	1563.730	1490.400
No. of unfilled grain plant^{-1}	0.009**	7.711	<0.000***	19.897	0.177	1.696	556.510	385.123
Thousand kernel weight (TKW)	0.154	2.153	<0.000***	2387.456	0.408	1.033	23.263	23.096
Grain yield plant^{-1}	0.031*	5.176	<0.000***	35.249	0.035*	3.026	245.273	268.394
Straw yield plant^{-1}	0.018	6.345	<0.000***	11.885	0.066	2.502	20.394	24.297
Harvest index (%)	0.507	0.451	<0.000***	14.024	0.243	1.448	51.895	53.072
Physiological								
NDVI	0.219	1.527	<0.000***	6.091	0.192	1.554	0.672	0.680
SR	0.304	1.067	<0.000***	6.920	0.138	1.780	5.202	5.318
SIPI	0.180	1.818	<0.000***	6.987	0.380	1.060	0.738	0.744
Lic2	0.632	0.231	0.012*	3.328	0.146	1.743	0.682	0.686
PRI	0.267	1.241	<0.000***	9.358	0.893	0.276	0.041	0.045

Note: NDVI = Normalized difference vegetation index, SR = Simple ratio, SIPI = Structure intensive pigment index, Lic2 = Lichtenthaler indices 2, PRI = Photochemical reflectance index.

Further analysis showed that within the varieties, Kasalath-type varieties showed less *OsORAP1* expression in both EDU treated varieties and control varieties except Kasalath ~~because the Kasalath variety is highly tolerance and it is not responsive to EDU treatment [36]~~, while the Nipponbare-types showed higher significant *OsORAP1* expression in control varieties than EDU treated varieties except Binadhan-16 and BRRI hybrid dhan3 which showed higher *OsORAP1*expression but not significant (Fig 2). These results suggest that the expression of *OsORAP1* is more responsive to EDU application in Nipponbare-type varieties than Kasalath-type varieties.

Formatted: Indent: First line: 0.5"

Fig 2. Expression of *OsORAP1* in 5 Kasalath-type and 5 Nipponbare-type rice varieties exposed to ambient ozone stress with the treatment of EDU and without EDU (control) at Real-time PCR data. Means values and standard errors (n = 3) are shown. Asterisks indicate significant differences. *, $P < 0.05$; **, $P < 0.01$; ***, $P < 0.001$; no asterisks means not significant.

3.3 Responses to ambient O_3 stress of the varieties carrying different alleles at *OsORAP1* locus due to EDU application

3.3.1. Leaf bronzing score

Upon prolonged exposure to ambient O_3 stress during the flowering or late flowering stage, visible symptoms of O_3 damage were observed. The formation of visible symptoms due to ambient ozone stress was found to differ significantly between the two allele groups when EDU treatments were applied. Nipponbare-type varieties displayed highly significant response, while Kasalath-type varieties showed insignificant response in EDU treatment. Again, Kasalath-type varieties showed insignificant response within the varieties and interaction between varieties and EDU treatment. On the other hand, Nipponbare-type varieties showed highly significant response within the variety but insignificant between varietal and EDU treatment interactions (Table 2).

Formatted: Indent: First line: 0.5"

In Nipponbare-type varieties, the response of EDU was prominent and the score in control plots were higher and significant, where the EDU treated varieties plots scores were less. Kasalath-type varieties response to EDU treatment were not significant except Kasalath because this variety is highly tolerant to ozone stress [16, ~~36~~37] and not responsive to EDU this is why the EDU treated Kasalath variety gave significant response while the control Kasalath variety did not (Fig 3).

Fig 3. Leaf bronzing scores of 5 Kasalath-type and 5 Nipponbare-type rice varieties exposed to ambient ozone concentration with and without application of ethylenediurea (EDU); bars indicate mean values of Mymensingh for Boro season 2022 with four replicates and standard errors (n=4); asterisks above the bars indicate significant difference between control and EDU treatment with one variety by Tukey's test (* $p<0.05$, ** $p<0.01$, *** $p<0.001$).

3.3.2. Yield parameters

The effects of Ethylenediurea (EDU) on yield parameters were investigated in both Kasalath-type and Nipponbare-type rice varieties, revealing varying responses across different parameters and varieties. In Kasalath-type varieties, significant responses to EDU treatment were observed for parameters including the number of ineffective tillers plant^{-1}, number of filled grains plant^{-1}, and straw yield plant^{-1}. However, other parameters such as the number of effective tillers plant^{-1}, number of unfilled grains plant^{-1}, thousand kernel weight (TKW), grain yield plant^{-1}, and harvest index (%) did not display significant responses to EDU treatment. Within the Kasalath-type varieties, responses were highly significant for all studied characters, but the interaction between EDU treatment and varieties was not significant except for grain yield plant^{-1}. Similarly, in Nipponbare-type varieties, significant responses to EDU treatment were observed for parameters such as the number of unfilled grains plant^{-1} and grain yield plant^{-1}. Conversely, other parameters like the number of effective tillers plant^{-1}, number of ineffective tillers plant^{-1}, number of filled grains plant^{-1}, TKW, straw yield plant^{-1}, and harvest index (%) did not exhibit significant responses to EDU treatment. Within the Nipponbare-type varieties, responses were highly significant for all studied characters, and the interaction between EDU treatment and varieties was highly significant for parameters including the number of effective tillers plant^{-1},

Formatted: Indent: First line: 0.5"

number of filled grains plant^{-1}, and grain yield plant^{-1} and other parameters were not significantly response (Table 2).

Specifically focusing on grain yield plant^{-1} and straw yield plant^{-1}, Kasalath-type varieties generally did not exhibit significant gains or losses in response to EDU treatment, except for BR11, which showed a significant increase in both parameters. Conversely, in Nipponbare-type varieties, BRRI hybrid dhan3 and BRRI dhan29 exhibited significant gains in grain yield plant^{-1} and straw yield plant^{-1} in response to EDU treatment, while other varieties did not show significant changes in either parameter (Table 3, Fig 4a and 4b).

Table 3. Parameters of contrasting rice varieties exposed to control (without EDU) and EDU treated varieties in ambient ozone stress conditions.

**Sl. No.**	**Name of the varieties**	**Grain yield plant^{-1} (g)**			**Straw yield plant^{-1} (g)**			**Harvest Index (%)**		
		**Control (Without EDU)**	**EDU treated**	**Gain / Loss (%)**	**Control (Without EDU)**	**EDU treated**	**Gain / Loss (%)**	**Control (Without EDU)**	**EDU treated**	**Gain / Loss (%)**
**Varieties with Kasalath-type *OsORAP1* allele**										
**1**	Binadhan-15	27.11	28.76	6.08	16.63	19.08	14.73	53.11	49.48	-7.34
**2**	Binadhan-17	27.11	26.74	-1.37	19.47	20.29	4.21	57.95	56.90	-1.84
**3**	BR11	27.92	37.66	34.88	20.85	25.89	24.17	47.69	53.17	10.31
**4**	Hutra	24.63	24.33	-1.22	16.35	18.21	11.38	51.91	49.13	-5.67
**5**	Kasalath	30.38	28.17	-7.27	20.82	21.46	3.07	50.92	48.49	-5.02
**Average**		27.43	29.13	6.20	18.82	20.99	11.51	52.32	51.43	-1.91
**Varieties with Nipponbare-type *OsORAP1* allele**										
**6**	Binadhan-12	23.82	23.26	-2.35	19.19	24.55	27.93	51.34	47.72	-7.59
**7**	Binadhan-16	25.40	26.09	2.71	15.19	11.66	-23.23	59.62	64.08	6.96
**8**	BRRI dhan28	18.16	16.48	-9.25	21.15	21.23	0.38	44.69	41.73	-7.10
**9**	BRRI dhan29	23.05	28.65	24.29	25.01	33.66	34.58	46.46	53.79	13.62
**10**	BRRI hybrid	32.35	39.72	22.78	21.43	30.38	41.76	57.37	58.05	1.71

	dhan3									
**Average**		24.53	26.84	9.41	20.39	24.30	19.13	51.90	53.07	1.52

Regarding the parameter harvest index (%), neither Kasalath-type nor Nipponbare-type varieties exhibited significant gains or losses due to EDU treatment (Table 3, Fig 4c). So, the variability in the response of different rice varieties to EDU treatment in terms of yield parameters, with some varieties exhibiting significant improvements while others show no significant response.

Formatted: Indent: First line: 0.5"

Fig 4. Yield parameters [a. Grain yield plant^{-1} b. Straw yield plant^{-1} and c. Harvest index (%)] of 5 Kasalath-type and 5 Nipponbare-type rice varieties grown in ambient ozone concentration with and without application of ethylenediurea (EDU); bars indicate mean values of Mymensingh for rainfed season 2022 with four replicates and standard errors (n=4); asterisks above the bars indicate significant difference between control and EDU treatment with one variety by Tukey's test (* $p<0.05$, ** $p<0.01$, *** $p<0.001$).

3.2.3. Reflectance indices

The impact of the EDU application on alleviating ambient ozone stress varied significantly across different parameters and rice varieties. Among Kasalath-type varieties, significant effects were observed for the parameters simple ratio (SR) and Lichtenthaler indices 2 (Lic2), while other reflectance indices such as normalized difference vegetation index (NDVI), structure intensive pigment index (SIPI), and photochemical reflectance index (PRI) did not show significant responses to the EDU treatment. However, within Kasalath-type varieties, notable responses were observed, indicating a significant interaction between varieties and EDU treatment across all studied reflectance indices parameters. Conversely, in Nipponbare-type varieties, the EDU treatment and its interaction with varieties did not yield significant responses

Formatted: Indent: First line: 0.5"

across any of the studied reflectance indices parameters. Nonetheless, significant differences were observed among the varieties themselves for all parameters, suggesting distinct genetic responses irrespective of the EDU treatment (Table 2).

Regarding the parameter simple ratio (SR), indicative of vegetation health, Kasalath-type varieties generally did not exhibit significant responses to the EDU treatment, except for Binadhan-17. Notably, Binadhan-17 displayed a significant response to EDU, possibly influenced by specific alleles or other abiotic factors. Conversely, Nipponbare-type varieties did not show significant responses to the EDU treatment, although there was an overall improvement in leaf greenness, except for Binadhan-12, possibly influenced by genetic or environmental factors (Fig 5a). For the structure intensive pigment index (SIPI), estimating the ratio of carotenoids to chlorophyll, both Kasalath-type and Nipponbare-type varieties displayed insignificant responses to the EDU treatment. However, Kasalath-type varieties showed higher responses overall to EDU, except for Kasalath itself. In contrast, Nipponbare-type varieties indicated higher responses to EDU, except for Binadhan-12, suggesting variability in response possibly due to genetic or environmental influences (Fig 5b). These findings suggest that EDU application can effectively mitigate ambient ozone stress in Nipponbare-type varieties, with different reflectance indices indicating its positive effects on leaf greenness, and photosynthesis content. "These findings suggest that EDU application can provide some degree of protection against ambient ozone stress in Nipponbare-type varieties. Although the variation for certain reflectance indices under treatment and interaction effects was not statistically significant, trends in leaf greenness and photosynthesis-related parameters indicate a positive influence of EDU. Overall, the study underscores the variability in the response of rice varieties to the EDU

treatment in mitigating ambient ozone stress, influenced by genetic factors, environmental conditions, and interactions between varieties and treatments.

Fig 5. Physiological traits [a. Simple ratio (SR) and b. Structure insensitive pigment index (SIPI)] of 5 Kasalath-type and 5 Nipponbare-type rice varieties grown in ambient ozone concentration with and without application of ethylenediurea (EDU); bars indicate mean values of Mymensingh for rainfed season 2022 with four replicates and standard errors (n=4); asterisks above the bars indicate significant difference between control and EDU treatment with one variety by Tukey's test (* $p<0.05$, ** $p<0.01$, *** $p<0.001$).

4. Discussion

4.1. Detrimental ambient ozone stress and *OsORAP1* expression in different rice varieties

Rice is an important staple food for more than half of the world's population [38]. However, rice production is threatened by various environmental stresses, such as ozone pollution [39]. Ozone is a major air pollutant that causes significant damage to crops, including rice [40, 41]. The rising tropospheric ozone concentrations in South Asia is very explicit [41]. It is one of the most important stress for rice production because rice production is now greatly hampered this region [16]. All of the varieties that we selected for our study were based on Bangladeshi rice production and these are the popular varieties of Bangladesh. To study this, we selected 10 rice varieties where 5 were carrying Kasalath type allele and 5 were carrying Nipponbare type allele. The OzT9 locus, previously identified as a QTL affecting foliar symptom formation in ozone stress, was used as a marker for ozone tolerance [24]. The ozone

exposure level is continuously observed in crop-growing environments in South Asia, and daytime ozone peaks exceeding 80 ppb frequently occur, especially from February to April [42]. Still, during the study period i.e. in rainfed season, it was observed that the daytime ozone peaks in Mymensingh, Bangladesh, exceeded the average 70 ppb (Fig 1). Thus, the ambient ozone exposure in the field is important for breeding ozone-tolerant rice on the South Asian subcontinent, especially Bangladesh. In this study comparing *OsORAP1* expression between Kasalath and Nipponbare rice types under EDU treatment, both types exhibited highly significant differences ($P < 0.001$) in response to the treatment, as well as in varietal differences and their interactions (Table 1). Further analysis revealed that within the Kasalath-type group, there was generally lower *OsORAP1* expression in both EDU-treated and control varieties, except for the Kasalath variety itself, which showed high tolerance and did not respond to EDU treatment (not responsive to EDU) [36]. On the other hand, the Nipponbare-types displayed higher *OsORAP1* expression in the control varieties compared to EDU-treated ones. Notably, Binadhan-16 and BRRI hybrid dhan3, within the Nipponbare group, exhibited higher *OsORAP1* expression, though these increases were not statistically significant (Fig 2). This suggests a complex interaction between variety type and response to EDU treatment, with Kasalath showing inherent tolerance and Nipponbare-types being more sensitive to the conditions tested.

One of the mechanisms by which plants respond to ozone stress is through the upregulation of the expression of various genes involved in stress tolerance. One such gene is *OsORAP1*, which is involved in oxidative stress response and has been shown to be upregulated in response to ozone stress in rice. Previous studies have shown that the presence of the QTL affects traits of agronomic importance such as spikelet sterility [44] and chalkiness [45] and the quality of rice straw [46]. Although the QTL has not been fine mapped or cloned to date,

experiments with rice mutants suggested that the gene *OsORAP1* may be underlying the QTL effect [7]. We analyzed the *OsORAP1* gene in 20 rice varieties and found that the polymorphisms occurring in the original two contrasting clades are largely conserved. We observed a dramatic difference in terms of foliar symptom formation between the two allele classes, Kasalath type and Nipponbare type (Fig.3). The likely mechanism underlying this phenomenon is that *OsORAP1* is involved in the oxidative cell death cycle, which leads to the induction of programmed cell death in ozone stress [47]. The transcriptional regulation of *OsORAP1* appears to constitute an important switch for the induction of programmed cell death under ozone stress in rice. This information is crucial for rice breeders to develop ozone tolerant rice varieties that can withstand the rising tropospheric ozone concentrations in South Asia.

4.2. Response of EDU treatment on different alleles at *OsORAP1* locus for yield and physiological parameters

Tropospheric ozone concentration is increasing throughout the Asian countries due to influence of the activities of human, population growth and rapid development of industry and economy [48]. Thus it is very crucial hazardous gases concisely effecting the agricultural crop production [49] now and even in the future, the crop production will be challenged without using tolerant varieties [22]. Several studies have demonstrated that EDU treatment can effectively mitigate the negative impact of ambient O_3 stress on plant growth, yield, and physiological processes of rice [16, ~~36~~37, 50]. The underlying mechanism behind the protective role of EDU treatment on plants is thought to be due to its ability to scavenge reactive oxygen species and reduce oxidative damage in plants by protects the surface [51]. The Leaf Bronzing Score (LBS) is commonly employed as a phenotypic measure to assess variances in rice genotypes regarding

Formatted: Indent: First line: 0.5"

their tolerance to diverse abiotic stresses, including ozone stress, zinc (Zn) deficiency, salinity, or iron toxicity [52, 53, 54]. Leaf bronzing score which is the marker of oxidative stress enriched the tolerant mechanism of Kasalath-type *OsORAP1* allele where the Nipponbare-type *OsORAP1* allele boosted the expression of gene in hazardous ozone concentrated area. Similarly, in our study, after application of EDU to the Kasalath-type and Nipponbare-type varieties, Kasalath-type varieties show tolerant i.e. less/no leaf bronzing score to EDU and insignificant response while the Nipponbare-type showed highly significant response and higher leaf bronzing score (Table 2). So, the Nipponbare-type varieties were EDU responsive i.e. EDU protects the plants from prolonged cell death [47] and Kasalath-type varieties were less/no EDU responsive i.e. the plants tolerant mechanism itself protects the plants from visible leaf injury (Fig 3). Different antioxidative defense mechanisms may account for the differential response of these two allele groups to ambient ozone stress. The findings of the present study are consistent with previous research that reported the efficacy of EDU treatment in reducing the adverse effects of ambient O_3 stress in plants [55].

EDU treatment in EDU responsive varieties i.e. Nipponbare type varieties increases the grain yield of rice moderately or not significantly [~~36~~37, 49, 55]. In Kasalath-type rice varieties, notable reactions in Kasalath-type *OsORAP1* allele to the application of EDU treatment were noted across various parameters. Conversely, in Nipponbare-type rice varieties, distinct responses to the EDU treatment were observed in Nipponbare-type *OsORAP1* allele, primarily reflected in alterations in the number of unfilled grains plant^{-1} and the resultant grain yield plant^{-1} (Table 2). Focusing specifically on grain yield per plant and straw yield per plant, the response to EDU treatment varied among Kasalath-type rice varieties. Generally, there were no substantial gains or losses observed, with the exception of BR11, which exhibited a notable increase in both

parameters following the treatment may be due to genetic or environmental influences i.e. involvement of some other allele responsible other than *OsORAP1* allele. In contrast, among Nipponbare-type rice varieties, BRRI hybrid dhan3 and BRRI dhan29 showed significant increases in grain yield per plant and straw yield per plant in response to EDU treatment in Nipponbare-type *OsORAP1* allele similar to observations reported by Ashrafuzzaman *et al.* [3637] in BRRI dhan28 (BR28), while other varieties did not demonstrate significant changes in either parameter (Table 3, Fig 4a and 4b) and. other varieties did not exhibit statistically significant changes. However, other Nipponbare-type varieties still demonstrated higher grain and straw yields, which may be influenced by genetic or environmental factors. -Similarly, the cultivars such as BRRI dhan28 and BRRI dhan29 underwent prior evaluation in controlled environmental trials regarding their reaction to ozone, which were extensively cultivated varieties tailored for the rainfed season, exhibited a yield reduction of 30 to 40% in open-top chamber experiments when exposed to ozone levels ranging from 77 to 100 ppb throughout the growth period [3637, 57]. By increasing the number of filled grains per plant, EDU increases the yield of rice varieties [16] with Nipponbare-type *OsORAP1* alleles. EDU treatment has diverse effects on *OsORAP1* alleles in Kasalath and Nipponbare rice varieties, emphasizing the importance of tailoring agricultural practices for optimal yield outcomes. In Table 3, the reduction in yield and related parameters upon EDU treatment in Kasalath-type genotypes (Binadhan-17, Hutra, and Kasalath), as well as in some Nipponbare-type genotypes, may indicate that EDU has little to no protective effect on ozone-tolerant genotypes. These observations underscore the complex interplay of genetic makeup and environmental conditions in determining the physiological responses of rice varieties to agricultural treatments.

The impact of EDU application on mitigating ambient ozone stress varied significantly across parameters and the Kasalath-type and Nipponbare-type rice varieties. To assess the physiological yield response to EDU in both allele varieties, various reflectance indices were gathered. NDVI was employed to assess chlorophyll levels at both the leaf and canopy or ecosystem scales [3637]. In our study NDVI, SIPI and PRI were not significant due to EDU treatment in both the allele groups but showed higher response (Table 2) and in previous studies, NDVI was significantly increased by EDU treatment in rice [16], wheat [58] and soybeans [59]. Previously, Lic2 has been proposed as a reliable physiological marker of ozone stress, conducive to high-throughput and non-invasive measurement through spectral reflectance [60]. Similarly, PRI has been recommended as a remote sensing technique for detecting ozone stress in vegetation before visible symptoms manifest [61]. Among Kasalath-type varieties, significant effects were noted for SR and Lic2, with notable interactions observed across all reflectance indices. Conversely, in Nipponbare-type varieties, no significant responses were observed for the studied reflectance indices (Table 2). While SIPI and SR responses were insignificant, Kasalath-type generally exhibited higher responses except Kasalath itself, contrasting with Nipponbare-type (Fig 5a and 5b). These results highlight the nuanced interactions between EDU treatment and rice varieties in combating ozone stress, emphasizing the role of genetic and environmental factors responsible for the EDU response in Kasalath-type and Nipponbare-type varieties. These findings have important implications for the selection of rice varieties for cultivation in areas with high ambient ozone levels, and underscore the need for further research to elucidate the molecular mechanisms underlying the observed differences in response to ozone stress between these two allele groups.

5. Conclusions

The response of different rice varieties, specifically Kasalath-type and Nipponbare-type, to Ethylenediurea (EDU) treatment under ambient ozone stress showed distinct genetic responses between these varieties. Nipponbare-type varieties displayed significant increases in *OsORAP1* expression and ~~visible symptom formation~~ leaf bronzing score in response to EDU treatment, while Kasalath-type varieties showed less significant responses due to their inherent tolerance to ozone stress. Additionally, the effects of EDU on yield parameters varied among the varieties. Kasalath-type varieties showed significant responses in certain parameters like the number of ineffective tillers plant^{-1} and straw yield plant^{-1}, whereas Nipponbare-type varieties responded significantly in parameters such as the number of unfilled grains plant^{-1} and grain yield plant^{-1}. Furthermore, the impact of EDU treatment on alleviating ambient ozone stress differed across reflectance indices parameters, with notable variability observed between Kasalath-type and Nipponbare-type varieties. Overall, the study highlights the intricate interplay of genetic factors, environmental conditions, and treatment interactions in influencing the response of rice varieties to EDU treatment in mitigating ambient ozone stress. Building on the findings of this study, future research should aim to identify the genetic mechanisms and also the understanding the role of *OsORAP1* that drive the differential responses of Kasalath-type and Nipponbare-type rice varieties to EDU treatment under ambient ozone stress, to enhance ozone tolerance and improve yield stability.

~~Declaration of Competing Interest~~

~~The authors declare that they have no conflict of interest.~~

~~Data Availability Statement~~

~~All data are in the manuscript and/or supporting information files.~~

Formatted: Indent: First line: 0.5"

Formatted: Indent: First line: 0.5"

Formatted: Indent: First line: 0.5"

Acknowledgment

The author thanks to Professor Dr. Michael Frei, Department of Agronomy and Crop Physiology, Justus-Liebig-University, Giessen, Germany, for his continuous support including finance for the field experiment and his lab research facilities.

Author Contribution

Rigyan Gupta: Methodology, Investigation, Data Curation, Formal Analysis, Software, Resources, Writing - Original Draft; Md Ashrafuzzaman: Conceptualization, Investigation, Writing - Review & Editing, Visualization, Validation, Project administration, and Supervision; Shamsul H. Prodhan: Writing - Review & Editing, and Supervision; Md. Nazmul Hasan: Investigation and Writing - Review & Editing; Shamsun Nahar Begum: Visualization, and Supervision; Mohammad Hasanuzzaman Rani: Writing - Review & Editing; Mirza Mofazzal Islam: Project administration, and Writing - Review & Editing.

References

1. Agathokleous E, Koike T, Watanabe M, Hoshika Y, Saitanis CJ. Ethylenediurea (EDU), an effective phytoproctectant against O_3 deleterious effects and a valuable research tool. J. Agric. Meteorol. 2015; 71: 185-195. http://dx.doi.org/10.2480/agrmet.D-14-00017.
2. Ainsworth EA. Understanding and improving global crop response to ozone pollution. Plant. J. 2016; 90: 886–897. https://doi.org/10.1111/tpj.13298.

3. Tai APK, Val Martin M, Heald CL. Threat to future global food security from climate change and ozone air pollution. Nat. Clim. Chang. 2014; 4: 817–821. https://doi.org/10.1038/nclimate2317.
4. Paoletti E, Castagna A, Ederli L, Pasqualini S, Ranieri A, Manning WJ. Gene expression in snapbeans exposed to ozone and protected by ethylenediurea. Environ. Pollut. 2014; 193: 1–5. https://doi.org/10.1016/j.envpol.2014.06.001.
5. The Royal Society. Ground-level ozone in the 21st century: future trends, impacts and policy implications. 2008. The Royal Society, London, UK. https://royalsociety.org/-/media/policy/publications/2008/7925.pdf.
6. Yamaji K, Ohara T, Uno I, Tanimoto H, Kurokawa J, Akimoto H. Analysis of the seasonal variation of ozone in the boundary layer in East Asia using the Community Multi-scale Air Quality model: what controls surface ozone levels over Japan? Atmos. Environ. 2006; 40: 1856–1868. https://doi.org/10.1016/j.atmosenv.2005.10.067.
7. Ueda Y, Siddique S, Frei M. A novel gene, OZONE-RESPONSIVE APOPLASTIC PROTEIN1, enhances cell death in ozone stress in Rice. Plant Physiol. 2015b; 169: 873–889. https://doi.org/10.1104/pp.15.00956.
8. Avnery S, Mauzerall DL, Liu J, Horowitz LW. Global crop yield reductions due to surface ozone exposure: 2. Year 2030 potential crop production losses and economic damage under two scenarios of O_3 pollution. Atmos. Environ. 2011; 45(13): 2297–2309. https://doi.org/10.1016/j.atmosenv.2011.01.002.
9. Haagen-Smit AJ. Chemistry and physiology of Los Angeles smog. Ind. Eng. Chem. 1952; 44(6): 1342–1346. https://doi.org/10.1021/ie50510a045.

10. Rai R, Agrawal M. Evaluation of physiology and biochemical responses of two rice (*Oryza sativa* L.) cultivars to ambient air pollution using open top chambers at a rural site in India. Sci. Total Environ. 2008; 407: 679–69. https://doi.org/10.1016/j.scitotenv.2008.09.010.
11. Tiwari S, Rai R, Agrawal M. Annual and seasonal variations in tropospheric ozone concentrations around Varanasi. Int. J. Remote. Sens. 2008; 29: 4499–4514. https://doi.org/10.1080/01431160801961391.
12. Middleton JT, Kendrick JB, Schwalm, HW. Injury to Herbaceous Plants by Smog or Air Pollution. Plant. Disease Reptr. 1950; 34: 245. https:// https://hero.epa.gov/hero/index.cfm/reference/details/reference_id/14944.
13. Richards BL, Middleton JT, Hewitt WB. Air pollution with relation to agronomic crops. V. Oxidant stipple of grape. Agron. J. 1958; 50: 559-561. https://doi.org/10.2134/agronj1958.00021962005000090019x.
14. Cho K, Shibato J, Agrawal GK, Jung YH, Kubo A, Jwa NS, Tamogami S, Satoh K, Kikuchi S, Higashi T, Kimura S, Saji H, Tanaka Y, Iwahashi H, Masuo Y, Rakwal R. Integrated transcriptomics, proteomics, and metabolomics analyses to survey ozone responses in the leaves of rice seedlings. J. Proteome. Res. 2008; 7: 2980–2998. https://doi.org/10.1021/pr800128q.
15. Sarkar A, Agrawal SB. Identification of ozone stress in Indian rice through foliar injury and differential protein profile. Environ. Monit. Assess. 2010; 161: 283–302. https://doi.org/10.1007/s10661-008-0738-z.

16. Frei M, Ashrafuzzaman M, Piepho HP, Herzog E, Begum SN, Islam MM. Evidence for tropospheric ozone effects on rice production in Bangladesh. Sci. Total Environ. 2024; 909: 168560. https://doi.org/10.1016/j.scitotenv.2023.168560.

17. Carnahan JE, Jenner EL, Wat EKW. Prevention of ozone injury to plants by a new protectant chemical. Phytopathol. 1978; 68: 1225–1229. https://www.apsnet.org/publications/phytopathology/backissues/Documents/1978Articles/Phyto68n08_1225.PDF.

18. Feng Z, Pang J, Nouchi I, Kobayashi K, Yamakawa T, Zhu J. Apoplastic ascorbate contributes to the differential ozone sensitivity in two varieties of winter wheat under fully open-air field conditions. Environ. Pollut. 2010a; 158: 3539–3545. https://doi.org/10.1016/j.envpol.2010.08.019.

19. Manning WJ, Paoletti E, Sandermann H, Ernst D. Ethylenediurea (EDU): a research tool for assessment and verification of the effects of ground level ozone on plants under natural conditions. Environ. Pollut. 2011; 159: 3283-3293. http://dx.doi.org/10.1016/j.envpol.2011.07.005.

20. Paoletti E, Contran N, Manning WJ, Ferrara AM. Use of the antiozonant ethylenediurea (EDU) in Italy: Verification of the effects of ambient ozone on crop plants and trees and investigation of EDU's mode of action. Environ. Pollut. 2009; 157: 1453–1460. https://doi.org/10.1016/j.envpol.2008.09.021.

21. Agathokleous E. Perspectives for elucidating the ethylenediurea (EDU) mode of action for protection against O_3 phytotoxicity. Ecotoxicol. Environ. Saf. 2017; 142: 530–537. https://doi.org/10.1016/j.ecoenv.2017.04.057.

22. Frei M. Breeding of ozone resistant rice: relevance, approaches and challenges. Environ. Pollut. 2015; 197: 144–155. https://doi.org/10.1016/j.envpol.2014.12.011.

23. Ueda Y, Siddique S, Frei M. A novel gene, ozone-responsive apoplastic protein1, enhances cell death in ozone stress in rice. Plant. Physiol. 2015a; 169(1): 873–889. https://doi.org/10.1104/pp.15.00956.

24. Frei M, Tanaka JP, Wissuwa M. Genotypic variation in tolerance to elevated ozone in rice: Dissection of distinct genetic factors linked to tolerance mechanisms. J. Exp. Bot. 2008; 59(13): 3741–3752. https://doi.org/10.1093/jxb/ern222.

25. Frei M, Tanaka JP, Chen CP, Wissuwa M. Mechanisms of ozone tolerance in rice: Characterization of two QTLs affecting leaf bronzing by gene expression profiling and biochemical analyses. J. Exp. Bot. 2010; 61(5): 1405–1417. https://doi.org/10.1093/jxb/erq007.

26. Luwe M, Takahama U, Heber U. Role of ascorbate in detoxifying ozone in the apoplast of spinach (Spinacia oleracea L.) leaves. Plant. Physiol. 1993; 101: 969–976. https://doi.org/10.1104/pp.101.3.969.

27. Plöchl M, Lyons T, Ollerenshaw J, Barnes J. Simulating ozone detoxification in the leaf apoplast through the direct reaction with ascorbate. Planta. 2000; 210: 454–467. https://doi.org/10.1007/PL00008153.

28. Feng Z, Wang S, Szantoi Z, Chen S, Wang X. Protection of plants from ambient ozone by applications of ethylenediurea (EDU): a meta-analytic review. Environ. Pollut. 2010b; 158: 3236-3242. http://dx.doi.org/10.1016/j.envpol.2010.07.009.

29. Sanmartin M, Drogoudi PA, Lyons T, Pateraki I, Barnes J, Kanellis AK. Over-expression of ascorbate oxidase in the apoplast of transgenic tobacco results in altered ascorbate and

glutathione redox states and increased sensitivity to ozone. Planta. 2003; 216: 918–928. https://doi.org/10.1007/s00425-002-0944-9.

30. Ashrafuzzaman M, Ueda Y, Frei M. Natural sequence variation at the *OsORAP1* locus is a marker for ozone tolerance in Asian rice. Environ. Exp. Bot. 2020; 178 (104153). https://doi.org/10.1016/j.envexpbot.2020.104153

31. Jain M, Nijhawan A, Tyagi AK, Khurana JP. Validation of housekeeping genes as internal control for studying gene expression in rice by quantitative real-time PCR. Biochem. Biophys. Res. Commun. 2006; 345(2): 646-51. https://doi.org/10.1016/j.bbrc.2006.04.140

32. Bates D, Mächler M, Bolker B, Walker S. Fitting Linear Mixed-Effects Models Using lme4. J. Stat. Softw. 2015; 67(1): 1–48. https://doi.org/10.18637/jss.v067.i01.

33. Kuznetsova A, Brockhoff PB, Christensen RHB. lmerTest Package: Tests in Linear Mixed Effects Models. J. Stat. Softw. 2017; 82(13): 1–26. https://doi.org/10.18637/jss.v082.i13

34. Hothorn T, Bretz F, Westfall P. Simultaneous Inference in General Parametric Models. Biom. J. 2008; 50(3): 346–363. https://CRAN.R-project.org/package=multcomp.

35. Lüdecke D, Ben-Shachar MS, Patil I, Waggoner P, Makowski D. Performance: An R Package for Assessment, Comparison and Testing of Statistical Models. J. Open Source Softw. 2021; 6(60): 3139. https://doi.org/10.21105/joss.03139.

36. Ren S, Stroud C, Belair S, Leroyer S, Moran M, Zhang J, Akingunola A, Makar P. Impact of Urban Land Use and Anthropogenic Heat on Air Quality in Urban Environments. In International Technical Meeting on Air Pollution Modelling and its

Application; Springer Proceedings in Complexity; Springer: Cham, Switzerland, 2020; 153–158. https://doi.org/10.1007/978-3-030-22055-6_24

36.37. Ashrafuzzaman M, Lubna FA, Holtkamp F, Manning WJ, Kraska T, Frei M. Diagnosing ozone stress and differential tolerance in rice (*Oryza sativa* L.) with ethylenediurea (EDU). Environ. Pollut. 2017; 230: 339-350. http://dx.doi.org/10.1016/j.envpol.2017.06.055.

37.38. ~~Ren S, Stroud C, Belair S, Leroyer S, Moran M, Zhang J, Akingunola A, Makar P. Impact of Urban Land Use and Anthropogenic Heat on Air Quality in Urban Environments. In International Technical Meeting on Air Pollution Modelling and its Application; Springer Proceedings in Complexity; Springer: Cham, Switzerland, 2020; 153–158. https://doi.org/10.1007/978-3-030-22055-6_24~~

38.39. Fukagawa NK, Ziska LH. Rice: Importance for Global Nutrition. J. Nutr. Sci. Vitaminol. 2019; 65: S2-S3. https://doi.org/10.3177/jnsv.65.S2.

39.40. Zhang G, Hu Y, Pan X, Cao R, Hu Q, Fu R, Risalat H, Shang B. Effects of increased ozone on rice panicle morphology. iScience. 2023; 26(4): 106471. https://doi.org/10.1016/j.isci.2023.106471.

40.41. Xu J, Dong X, Zhang T, Liu J, Tao S. 2022. Mitigation of air pollutant impacts on rice yields in China by sector. Environ. Res. Lett. 2022; 17: 054037. DOI 10.1088/1748-9326/ac681c.

41.42. Anagha KS, Kuttippurath J, Sharma M, Cuesta J, A comprehensive assessment of yield loss in rice due to surface ozone pollution in India during 2005–2020: A great concern for food security. Agric. Syst. 2024; 215:103849. https://doi.org/10.1016/j.agsy.2023.103849.

~~42.~~43. Li S, Yang Y, Wang H, Li P, Li K, Ren L, Wang P, Li B, Mao Y, Liao H. Rapid increase in tropospheric ozone over Southeast Asia attributed to changes in precursor emission source regions and sectors. Atmos. Environ. 2023; 304: 119776. https://doi.org/10.1016/j.atmosenv.2023.119776.

~~43.~~44. Debaje SB, Kakade AD, Jeyakumar SJ. Air pollution effect of O_3 on crop yield in rural India. J. Hazard. Mater. 2010; 183: 773–779. https://doi.org/10.1016/j.jhazmat.2010.07.093.

~~44.~~45. Wang Y, Yang L, Höller M, Zaisheng S, Pariasca-Tanaka J, Wissuwa M, Frei M. Pyramiding of ozone tolerance QTLs *OzT8* and *OzT9* confers improved tolerance to season-long ozone exposure in rice. Environ. Exp. Bot. 2014; 104: 26–33. https://doi.org/10.1016/j.envexpbot.2014.03.005.

~~45.~~46. Jing LQ, Dombinov V, Shen SB, Wu YZ, Yang LX, Wang YX, Frei M. Physiological and genotype-specific factors associated with grain quality changes in rice exposed to high ozone. Environ. Pollut. 2016; 210: 397–408. https://doi.org/10.1016/j.envpol.2016.01.023.

~~46.~~47. Frei M, Kohno Y, Wissuwa M, Makkar HPS, Becker K. Negative effects of tropospheric ozone on the feed value of rice straw are mitigated by an ozone tolerance QTL. Glob. Change Biol. 2011; 17: 2319–2329. https://doi.org/10.1111/j.1365-2486.2010.02379.x.

~~47.~~48. Kangasjarvi J, Jaspers P, Kollist H. Signalling and cell death in ozone-exposed plants. Plant Cell Environ. 2005; 28: 1021–1036. https://doi.org/10.1111/j.1365-3040.2005.01325.x.

~~48.~~49. Zeng Y, Cao Y, Qiao X, Seyler BC, Tang Y. Air pollution reduction in China: Recent success but great challenge for the future. Sci. Total Environ. 2019; 663: 329–337. https://doi.org/10.1016/j.scitotenv.2019.01.262.

~~49.~~50. Sampedro J, Waldhoff ST, Van de Ven DJ, Pardo G, Van Dingenen R, Arto I, Prado A, Sanz MJ. Future impacts of ozone driven damages on agricultural systems. Atmos. Environ. 2020; 231: 117538. https://doi.org/10.1016/j.atmosenv.2020.117538.

~~50.~~51. Shang B, Fu R, Agathokleous E, Dai L, Zhang G, Wu R, Feng Z. Ethylenediurea offers moderate protection against ozone-induced rice yield loss under high ozone pollution. Sci. Total Environ. 2022; 806(3): 151341. https://doi.org/10.1016/j.scitotenv.2021.151341.

~~51.~~52. Ashrafuzzaman M, Zahidul H, Ali B, Mathew B, Yu P, Hochholdinger F, Neto JBA, McGillen MR, Ensikat HJ, Manning WJ, Frei M. Ethylenediurea (EDU) mitigates the negative effects of ozone in rice: Insights into its mode of action. Plant Cell Environ. 2018; 41:2882–2898. https://doi.org/10.1111/pce.13423.

~~52.~~53. Quijano-Guerta C, Kirk GJD, Portugal AM, Bartolome VI, McLaren GC. Tolerance of rice germplasm to zinc deficiency. Field Crops Res. 2002; 76: 123-130. https://doi.org/10.1016/S0378-4290(02)00034-5.

~~53.~~54. Sohn JK, Lee JJ, Kwon YK, Kim KM. Varietal differences and inheritance of resistance to ozone stress in rice (Oryza sativa L.). SABRAO J. Breed. Genet. 2002; 34: 65-71. https://www.cabidigitallibrary.org/doi/full/10.5555/20033016398.

~~54.~~55. Takehisa H, Ueda T, Fukuta Y, Obara M, Abe T, Yano M, Yamaya T, Kameya T, Higashitani A, Sato T. Epistatic interaction of QTLs controlling leaf bronzing in rice

(Oryza sativa L.) grown in a saline paddy field. Breed. Sci. 2006; 56: 287-293. https://doi.org/10.1270/jsbbs.56.287.

55.56. Surabhi S, Pande V, Pandey V. Ethylenediurea (EDU) mediated protection from ambient ozone-induced oxidative stress in wheat (*Triticum aestivum* L.) under a high CO_2 environment. Atmos. Pollut. Res. 2022; 13(8): 101503. https://doi.org/10.1016/j.apr.2022.101503.

56.57. Pandey AK, Majumder B, Keski-Saari S, Kontunen-Soppela S, Mishra A, Sahu N, *et al*. Searching for common responsive parameters for ozone tolerance in 18 rice cultivars in India: results from ethylene diurea studies. Sci. Total Environ. 2015; 532: 230–238. https://doi.org/10.1016/j.scitotenv.2015.05.040.

57.58. Akhtar N, Yamaguchi M, Inada H, Hoshino D, Kondo T, Fukami M, *et al*. Effects of ozone on growth, yield and leaf gas exchange rates of four Bangladeshi cultivars of rice (*Oryza sativa* L.). Environ. Pollut. 2010; 158: 2970–2976. https://doi.org/10.1016/j.envpol.2010.05.026.

58.59. Gupta SK, Sharma M, Majumder B, Maurya VK, Lohani M, Deeba F, *et al.* Impact of Ethylenediurea (EDU) on growth, yield and proteome of two winter wheat varieties under high ambient ozone phytotoxicity. Chemosphere. 2018; 196: 161–173. https://doi.org/10.1016/j.chemosphere.2017.12.150.

59.60. Rai R, Agrawal M, Choudhary KK, Agrawal SB, Emberson L, Büker P. Application of ethylene diurea (EDU) in assessing the response of a tropical soybean cultivar to ambient O3: Nitrogen metabolism, antioxidants, reproductive development and yield. Ecotoxicol. Environ. Saf. 2015; 112: 29–38. https://doi.org/10.1016/j.ecoenv.2014.10.031.

~~60.~~61. Begum H, Alam MS, Feng Y, Koua P, Ashrafuzzaman M, Shrestha A, *et al.* Genetic dissection of bread wheat diversity and identification of adaptive loci in response to elevated tropospheric ozone. Plant Cell Environ. 2020; 43: 2650–2665. https://doi.org/10.1111/pce.13864.

62. Meroni M, Panigada C, Rossini M, Picchi V, Cogliati S, Colombo R. Using optical remote sensing techniques to track the development of ozone-induced stress. Environ. Pollut. 2009; 157: 1413–1420. https://doi.org/10.1016/j.envpol.2008.09.018.

Supporting information

S1 Fig. The DNA profile of 20 rice genotypes using primer KAS_1_2, which showing the band on Kasalath-type (specific) genotypes

S2 Fig. Photographs of different rice varieties under ambient ozone stress conditions with and without the application of EDU during the irrigated (Boro) season at Mymensingh, Bangladesh (2022). [Shown are images of six rice variety with two treatments: (a) BRRI hybrid dhan3 without EDU (Control), (b) BRRI hybrid dhan3 with EDU, (c) BRRI dhan28 without EDU (Control), (d) BRRI dhan28 with EDU, (e) BRRI dhan29 without EDU (Control), and (f) BRRI dhan29 with EDU. Plants were exposed to ambient ozone stress, and EDU (ethylene diurea) was applied to assess its protective effects against ozone stress]

S1 File. Ozone concentration (ppb) data during the growing period

S2 File. Yield and yield contributing characters data of Kasalath and Nipponbare type varieties at ambient ozone stress with EDU and without EDU (control) application

S3 File. Reflectance index data of Kasalath and Nipponbare type varieties at ambient ozone stress with EDU and without EDU (control) application

S4 File. Expression analysis data from delta-delta CT quantification method

~~61.~~

Formatted: Normal, Indent: Left: 0.5", No bullets or numbering

Formatted: Font: (Default) Times New Roman, 18 pt

Formatted: Normal, No bullets or numbering

Formatted: Font: 12 pt

Formatted: Normal, Indent: Left: 0.25", No bullets or numbering

Formatted: Font: 18 pt

Formatted: Normal, Left, Space After: 0 pt, Line spacing: single, No bullets or numbering, Adjust space between Latin and Asian text, Adjust space between Asian text and numbers

Academic editor and Reviewer response

Academic editor

This work studied the ambient ozone effects in rice among the varieties having different haplotypes of OsORAP1 gene. Though the work provides new information on the EDU and OsORAP1 alleles, the haplotypes characterization between the genotypes studied, effects of other covariates on yield related traits are not properly addressed as this study was performed in field conditions.

Author response: Thank you for your thoughtful feedback. We acknowledge that while our study presents new information on the EDU treatment and OsORAP1 alleles, the haplotype characterization between the genotypes, as well as the effects of other covariates on yield-related traits, could have been explored in more depth. Given that this study was conducted under field conditions, it is indeed challenging to fully account for all environmental variables and covariates that might influence yield-related traits.

Our primary focus was on correlating OsORAP1 allele expression with these traits, and while we made initial observations, we recognize that further analysis is needed to comprehensively address the role of other covariates on yield outcomes. We plan to build on this preliminary work in future studies, which will include a more detailed exploration of the interactions between haplotypes and additional covariates under controlled as well as field conditions.

Academic Editor

The Kasalath was used as control but its data was not consistent in responses to EDU compared to control, and the expression of OsORAP1 in Kasalath is not very clear.

Author response: Thank you for your valuable feedback. We would like to clarify that Kasalath is indeed a known ozone-tolerant variety, as reported by Frei et al. (2024) and Ashrafuzzaman et al. (2017). The differential response to EDU in Kasalath, compared to the control, can be attributed to its inherent tolerance to ozone. As such, EDU, which typically protects against ozone damage, may have a reduced or negligible effect in tolerant varieties like Kasalath.

We acknowledge that the expression of OsORAP1 in Kasalath is not entirely clear from the current data. This variability may be influenced by other environmental factors (such as temperature, humidity) or potential genetic factors that were not accounted for in the experimental setup. We are considering additional studies to better understand these influences and clarify the expression patterns of OsORAP1 under different conditions.

Academic Editor

The yield data of the current manuscript for BRRI dhan 28 and 29 or Kasalath were not consistent with their immediate past publication such as Frei et al. (2024).

Author response: We appreciate your observation regarding the yield data. The yield data presented in our current manuscript for BRRI dhan28, BRRI dhan29, and Kasalath is based on results from a single season (2022) and one location (Mymensingh, Bangladesh). In contrast, the study by Frei et al. (2024) reflects the average yield data collected across four locations and over three consecutive seasons. As a result, the differences in the yield data are likely due to variations in environmental conditions, management practices, and sampling durations between the two studies. We believe that this distinction explains the apparent inconsistency and highlights the importance of experimental context when interpreting yield outcomes.

Specific suggestions

Reviewer #1

Suggestion 1: Line 11, the abstract should succinctly and clearly summarize the research objectives, methods, results and conclusions of the paper. It is suggested to add some detailed data to show the variance of different varieties of rice in response to EDU, such as how much or ?% of rice yield and yield parameters were affected.

Author response: Thank you for your suggestion. Our primary focus in this study was to investigate the expression of the OsORAP1 allele following the application of EDU, rather than concentrating on the yield and yield parameters. While we acknowledge that adding specific data on how much or what percentage of rice yield and yield parameters were affected could provide additional context, the aim of this research was to highlight the molecular responses related to the OsORAP1 gene.

Suggestion 2: Line 47, 66, check the font.

Author response: Checked, the font is Times New Romans and size is 12 and O3 is corrected as O_3.

Suggestion 3: Line 98-105, It is suggested to refine the research target and scientific question and unify and clarify the study materials (10, 20, etc.) in the study.

Author response: We have accepted the reviewer comments and tried to refine, unify and clarify the research target.

Suggestion 4: The figures are in low resolution and hard to read, especially figures 4 and 5.

Author response: We have accepted the reviewer comments and the resolution of all the figures are now adjusted as per recommendation.

Suggestion 5: Cultivar screening studies through EDU (such as: Ethylenediurea (EDU) protects inbred but not hybrid cultivars of rice from yield losses due to surface ozone, https://doi.org/10.1007/s11356-021-15032-9) could be compared with this study.

Author response: Thank you for your insightful comment. In our study, we included only one indica hybrid rice variety, which limits direct comparison with the broader cultivar screening conducted in the referenced study (EDU protecting inbred but not hybrid cultivars). The focus of our research was more on the molecular response of the OsORAP1 gene to EDU treatment, rather than a comprehensive screening across multiple hybrid or inbred cultivars. Therefore, the scope and objectives of the two studies are not entirely comparable.

We acknowledge the value of such comparisons and may consider including a wider range of cultivars in future experiments to facilitate a more comprehensive analysis.

Reviewer #2

Query 1: First query was that how did you divide the rice varieties in Nipponbare type? The DNA band similar to Kasalath is Kasalath-type is acceptable, but there was no Nipponbare variety tested? Also, no reference was used.

Author response: The division of rice varieties into Nipponbare-type was based on the methodology established by Ashrafuzzaman et al. (2020). In their study, they classified rice varieties into two clades using two markers: Kasalath-type OsORAP1 allele and Nipponbare-type OsORAP1 allele. Their results showed that varieties could be identified by the presence of a band corresponding to either Kasalath-type or Nipponbare-type. In our study, we used only the Kasalath-type OsORAP1 allele as a marker. Varieties that showed a band were classified as Kasalath-type, while those that did not were considered Nipponbare-type.

Query 2: What is the theoretical or standard relationship between EDU and OsORAP1? Kasalath is an ozone tolerant variety and EDU is ozone protectant, and therefore, OsORAP1 expression is expected to be lower or no change. However, the expression of OsORAP1 expression was significantly increased with EDU treatment. Similar results found in leaf bronzing score of Kasalth in EDU. This indicate the inconsistency of the data. Because, Kasalath was used as control or/and reference of the ozone tolerance.

Author response: Thank you for your valuable comment. EDU is indeed widely recognized as an antiozonant, protecting plants from the adverse effects of ozone. The OsORAP1 gene, on the other hand, is associated with cell death and oxidative stress response, particularly under ozone stress conditions.

In the case of Kasalath, an ozone-tolerant variety, the expectation might be that EDU would result in no significant change or even a decrease in OsORAP1 expression. However, our findings showed a significant increase in OsORAP1 expression following EDU treatment in

Kasalath, which may seem counterintuitive at first. One possible explanation for this is that EDU does not entirely suppress all molecular responses to ozone stress but rather mitigates the downstream effects, such as cell death and visible damage. The observed increase in OsORAP1 expression could be part of a complex compensatory mechanism where EDU reduces the physiological damage, but the molecular signaling related to stress response is still activated.

Similarly, the increase in leaf bronzing score could be influenced by other environmental or genetic factors, despite Kasalath being used as a reference for ozone tolerance. We are currently exploring these dynamics further to better understand the relationship between EDU treatment and OsORAP1 expression in tolerant varieties like Kasalath.

Query 3: Another point is that the yield data of this study, especially for BRRI dhan 28 & 29, were not consistent with a recently published article by Frei et al. 2024.

Author response: We appreciate your observation regarding the yield data. The yield data presented in our current manuscript for BRRI dhan28, BRRI dhan29, and Kasalath is based on results from a single season (2022) and one location (Mymensingh, Bangladesh). In contrast, the study by Frei et al. (2024) reflects the average yield data collected across four locations and over three consecutive seasons. As a result, the differences in the yield data are likely due to variations in environmental conditions, management practices, and sampling durations between the two studies. We believe that this distinction explains the apparent inconsistency and highlights the importance of experimental context when interpreting yield outcomes.

Specific suggestions

Revise abstract based on the suggested revisions.

Suggestion 1: Line 15: Better to use the representative varieties in the -types

Author response: Thank you for the suggestion. The varieties in our study were classified into Kasalath-type and Nipponbare-type based on their genetic background and specific allelic differences in OsORAP1. We chose to use these classified types to provide a clearer understanding for readers and to emphasize the haplotype-based categorization. This approach allows us to better convey the genetic and phenotypic distinctions between the groups, which is central to the study’s findings.

Suggestion 2: Line 21: Better to add quantitative values in the text

Author response: Thank you for the suggestion. The varieties in our study were classified into Kasalath-type and Nipponbare-type based on their genetic background and specific allelic differences in OsORAP1. We chose to use these classified types to provide a clearer understanding for readers and to emphasize the haplotype-based categorization. This approach allows us to better convey the genetic and phenotypic distinctions between the groups, which is central to the study’s findings.

Suggestion 3: Line 42: Please revise or explain: 4% -1

Author response: We apologize for the oversight. The issue has been corrected in the text, and the range now reads as 4-17%.

Suggestion 4: Line 53~54: Better to add the reference of the severe damage due to ozone stress

Author response: Thank you for the suggestion. We have now included the appropriate reference to support the statement regarding the severe damage caused by ozone stress.

Suggestion 5: Line 100: Full form of OTC or FACE

Author response: Thank you for the suggestion. We have now provided the full forms of OTC (Open Top Chamber) and FACE (Free-Air Concentration Enrichment) in the text for clarity.

Suggestion 6: Line 102: Mention how did you measure?

Author response: Thank you for the suggestion. We have now included details on the measurement method, specifying that EDU was applied, and leaf samples were collected during the flowering stage to assess gene expression.

Suggestion 7: Line 108: elaboration of BINA

Author response: Thank you for the suggestion. We have now expanded BINA to Bangladesh Institute of Nuclear Agriculture in the text for clarity.

Suggestion 8: Table 1: How did you determine the type? Better to explain or add the reference Kasalath- and Nipponbare-type. Also, It would be good to add and show the bronzing score in leaf or damage photos in supplementary figure)

Author response: Thank you for the suggestion. We have now included the appropriate reference to explain the determination of Kasalath-type and Nipponbare-type. Additionally, as recommended, we have added contrasting images of the ozone damage plot (control) and the EDU-treated plot as supplementary figures to show the bronzing score and visible leaf damage.

Suggestion 9: Line 112: name the representative

Author response: Thank you for the suggestion. We have now named the representative genotypes in the text to support the statement.

Suggestion 10: Line 114: better to mention how many seeds per lines were sown

Author response: Thank you for the suggestion. We used 200g of seeds for each variety in a 1 x 1 m^2 seedbed and did not sow the seeds line by line.

Suggestion 11: Line 119: 35days old seedlings for transplantation? Is it ok? Seems bit old.

Author response: Thank you for the suggestion. Yes, it is correct. In the Boro (irrigated) season, farmers in Bangladesh typically use 35-40 day-old seedlings for transplantation, as the life cycle of the varieties grown during this season usually exceeds 130 days.

Suggestion 12: Line 140: How old the plants during DNA leaf sample collection

Author response: Thank you for the suggestion. The plants were 85 days old at the time of DNA leaf sample collection.

Suggestion 13: Line 166: Why in two groups? There was no Nipponbare variety tested? Better to write 'other type'

Author response: Thank you for the suggestion. While there was no Nipponbare variety tested in this study, we classified the varieties into two groups based on their haplotypes: Kasalath-type and Nipponbare-type. We already have included the appropriate reference to support this classification.

Suggestion 14: Line 173: plants were grown in natural field, it's not a treatment, right?

Author response: Thank you for the suggestion. While the plants were grown in a natural field, it was indeed part of the treatment. All plants were exposed to ambient ozone stress under natural field conditions. We applied two treatments: one group received EDU (Ethylenediurea) at a concentration of 300 ppm, sprayed once a week to mitigate ozone effects, while the control group was sprayed with an equivalent amount of water. This approach allowed us to assess the impact of EDU in comparison to untreated plants.

Suggestion 15: Line 181: why RNA-was isolated from the sample collected at flowering and late flowering. Why?

Author response: Thank you for the suggestion. RNA was isolated from samples collected during the flowering stage because this period is critical for assessing the effects of ambient ozone stress. At this stage, the plants exhibited visible leaf symptoms due to high ozone levels, and it represents the peak period of *OsORAP1* gene expression in the susceptible variety. This timing allows us to capture the gene's response to stress accurately.

Suggestion 16: Line 187: what is treatment chamber? Please explain

Author response: Thank you for the suggestion. We apologize for the error. The term "treatment chamber" should be corrected to "treatment plot." We will make this clarification in the text to accurately describe the experimental setup.

Suggestion 17: Fig 1: Why the Ozone has sharply increased after Jan 15?

Author response: Thank you for the suggestion. The sharp increase in ozone concentration after January 15 may be attributed to the enhanced intensity of sunlight and the flow of polluted air, which tends to be clearer of dust, fog etc. during daytime. These factors contribute to higher ozone levels in the atmosphere.

Reviewer #3

-Introduction-

Comment 1: Line 122- insert symbol instead of x

Author response: Thank you for the comment. We have corrected the "x" to the proper symbol "×" in the text.

Comment 2: Table 1- Serial number can be added in the rice varieties list.

Author response: Thank you for the comment. We have now included the serial numbers in table 1 for clarity.

Comment 3: Line 141- How much leaf sample was used for the study.

Author response: Thank you for the comment. We used 3-4 leaf samples for the study.

Comment 4: -How data from bands was statistically analyzed?

Author response: Thank you for the comment. We analyzed the expression value obtained from the delta-delta CT quantification method.

Comment 5: Line 173- Section 2.4 needs to be detailed, only control plants and ozone mitigation is briefly listed, how ozone stress was applied? Details of Ozone polluted location may be added in the material and methods section.

Author response: Thank you for the comment. We have revised Section 2.4 to include more detailed information on how ozone stress was applied, as well as additional details about the ozone-polluted location. All relevant information has been incorporated into the text.

Comment 6: Line 183- It will be better to mention Days to flowering, how many days after germination samples were taken?

Author response: Thank you for the comment. We have now included the days to flowering after germination in the text, which was approximately 115 days. However, we did not specify a uniform days to flowering as it varied across the different varieties used in the study.

Comment 7: -While the use of EDU is explained, there could be a more robust justification for the specific fertilizer choices and their timing, especially regarding their impact on ozone stress mitigation.

Author response: Thank you for the comment. The use of EDU in this study was specifically chosen for its well-documented role as an antiozonant, mitigating the negative effects of ozone stress on plants. Regarding the fertilizer choices and timing, standard fertilizer practices were followed in alignment with local agronomic recommendations to ensure optimal plant growth. The focus of the study was primarily on the impact of EDU on OsORAP1 expression in relation to ozone stress, and we aimed to minimize other variables. However, we agree that further research on the interaction between fertilizer application and ozone stress mitigation could provide additional insights.

Comment 8: Line 206- Instead of Delta Delta, symbol may be inserted.

Author response: Thank you for the suggestion. We have corrected the text and inserted the Δ-Δ symbol as recommended.

Comment 9: Line 225-226 - figure caption?

Author response: Thank you for the comment. We have included the figure caption at lines 225-226 in accordance with the journal's guidelines.

Comment 10: Fig. 2 instead of expression (relative gene expression should be used)

Author response: Thank you for the comment. We believe the current term "expression" is appropriate for the figure, as it aligns with the terminology used throughout the study. However, we will consider using "relative gene expression" for consistency if necessary.

-Discussion-

Comment 11: Simplifying the language/ structure could enhance readability.

Author response: Thank you for the comment. We have made efforts to simplify the language and structure throughout the manuscript to enhance readability while maintaining the scientific integrity of the content.

Comment 12: The discussion does not address any potential limitations of the study, such as environmental variability during the experiments or the specific conditions under which the results were obtained. Acknowledging these limitations could provide a more balanced perspective.

Author response: Thank you for the comment. We acknowledge that environmental variability and other genetic factors may have influenced the results of our study. While we took measures to minimize these impacts, such variability could not be entirely controlled in a field setting. We have already addressed these limitations in the Discussion section to provide a more balanced perspective on the study's findings.

Comment 13: Addition of more detailed analysis of the specific outcomes related to yield and physiological parameters could enhance the quality of discussion.

Author response: Thank you for the comment. We have provided a detailed explanation of the data obtained from both molecular and field-level analyses. The specific outcomes related to yield and physiological parameters were thoroughly discussed to ensure a comprehensive understanding of the results. We believe these discussions enhance the overall quality of the manuscript.

Reviewer #4

Major comments

Comment 1: Results to be updated with actual values rather than summary. Please include the mean difference, percent change, P values in the results section

Author response: Thank you for the comment. We have updated Table 1 based on a recently published article and believe that further updates may not be necessary. Additionally, P values have already been included as Pr(>F).

Comment 2: The marker to differentiate the Nipponbare and Kasalath alleles of OsORAP1 gene is not clear. Whether it is functional marker and reference may be included. A gel photo can be given in the manuscript showing the allelic differences. Further, gene sequence analysis would provide the clear differences between the genotypes.

Author response: Thank you for the comment. We have included the primer used to differentiate the Nipponbare and Kasalath alleles of the OsORAP1 gene, along with the appropriate reference. A gel photo showing the allelic differences between Kasalath-type and Nipponbare-type genotypes using the KAS 1_2 primer has been provided as a supplementary figure. Additionally, for the expression analysis, we conducted ΔΔCT analysis based on data obtained from qPCR.

Comment 3: Grain yield per plant of more than 200 is really very high. Average grain yield per plant in rice is ~ 30g. This is difficult to comprehend. Further, straw and grain ratio in rice is almost 1:1. However, the findings in this study showed 1:0.1. There is some issues in the data collection or reporting.

<u>Author response:</u> Thank you for the comment. We apologize for the mistake. The grain yield per plant and straw-to-grain ratio values have been corrected in the text to reflect accurate data.

Comment 4: Since this work is done in field conditions, how the temperature effects and other covariates are addressed in this study. EDU effects in grain filling also suits to minimizing the heat stress effects in rice. Therefore, limitations of this study may be highlighted in the conclusion part of the manuscript.

<u>Author response:</u> Thank you for the comment. We have addressed your comments in the conclusion section. However, EDU did not affect grain filling in this study, and heat stress was not a significant factor during the Boro (irrigated) season when the experiment was conducted.

Minor comments

Comment 1: In page2 line 23-26 write the sentence properly.

<u>Author response:</u> Thank you for the comment. We have revised the sentence on page 2, lines 23-26, to improve clarity and accuracy in the text.

Comment 2: Page 13 & 14, line 232-237, the sentence "expression in EDU treatment, variety and EDU treatment and varietal interaction" and in table2 "season with or without the application of ethylenediurea" is creating confusion, clarify it which one is control, and which one is treatment in table also.

<u>Author response:</u> Thank you for the comment. We have revised the sentences on pages 13 and 14 to clarify the distinctions between control and treatment groups regarding EDU treatment, varieties, and their interactions. Additionally, we have updated Table 2 to clearly indicate which conditions represent the control and which represent the treatment.

Comment 3: Throughout the manuscript the "control" one is written as "varieties" which is very confusing. The change of word "varieties" to "control" is recommended where it is addressing control one.

<u>Author response:</u> Thank you for the comment. In our study, we distinguished between two treatments: one with the application of EDU and one without (which we refer to as the control). We have used the term "EDU treated varieties" to specify those receiving the treatment, while "control varieties" refers to those without EDU application.

Comment 4: In page 8, line 159-161 only primer sequences are provided, the primers were designed specifically for this study or from any reference material. If from reference material, then please mention it.

Author response: Thank you for the comment. We have provided the primer names and their corresponding references in the text to clarify.

Comment 5: Gene name needs to be italized in the title

Author response: Thank you for the comment. We have italicized the gene name in the title as suggested.

Comment 6: A line may be mentioned in the Abstract the reason for selecting the EDU and OsORAP1 gene in O3 studies

Author response: Thank you for the comment. Added in the Abstract.

Comment 7: Ren et al 2020 which reported the 40ppb as threshold is not cited. However, few studies also reported 120 ppb as threshold. The Introduction and discussion should include relevant and recent literatures related to ozone toxicity in rice.

Author response: Thank you for the comment. We have included and cited the recent reference by Ren et al. (2020), which reported the 40 ppb ozone threshold level for crops.

Comment 8: How EDU treatment @ 300 ppm was fixed

Author response: Thank you for the comment. Different study showed that the 300ppm is the best effective EDU treatment dose there by we used this 300 ppm dose once a week after transplanting to harvest (Ashrafuzzaman et al. 2017, Ashrafuzzaman et al. 2018, Frei et al., 2023).

Comment 9: The unit of grain yield in Fig.4 and expression or relative fold change in Fig,2 needs to be updated

Author response: Thank you for the comment. The unit of grain yield in Fig.4 and expression or relative fold change in Fig 2 were updated.

Comment 10: Just want to know, Why in Kasalath, leaf bronzing is more in EDU treatment

Author response: Thank you for the comment. The increase in leaf bronzing score could be influenced by other environmental or genetic factors, despite Kasalath being used as a reference

for ozone tolerance. We are currently exploring these dynamics further to better understand the relationship between EDU treatment and OsORAP1 expression in tolerant varieties like Kasalath.

Comment 11: Surprised to see the sudden increase in Ozone levels from 15th Jan 2022, any specific reason for it

Author response: Thank you for the comment. Thank you for the suggestion. The sharp increase in ozone concentration after January 15 may be attributed to the enhanced intensity of sunlight and the flow of polluted air, which tends to be clearer of dust, fog etc. during daytime. These factors contribute to higher ozone levels in the atmosphere.

Academic editor and Reviewer response

Academic editor

Comments 1: Please ensure that your manuscript meets PLOS ONE's style requirements, including those for file naming. The PLOS ONE style templates can be found at

https://journals.plos.org/plosone/s/file?id=wjVg/PLOSOne_formatting_sample_main_body.pdf and

https://journals.plos.org/plosone/s/file?id=ba62/PLOSOne_formatting_sample_title_authors_affiliations.pdf

Author response: Thank you for your valuable comments. We have revised the manuscript to ensure it fully complies with PLOS ONE's style requirements, including formatting and file naming, as per the provided guidelines.

Comments 2: PLOS ONE now requires that authors provide the original uncropped and unadjusted images underlying all blot or gel results reported in a submission's figures or Supporting Information files. This policy and the journal's other requirements for blot/gel reporting and figure preparation are described in detail at https://journals.plos.org/plosone/s/figures#loc-blot-and-gel-reporting-requirements and https://journals.plos.org/plosone/s/figures#loc-preparing-figures-from-image-files. When you submit your revised manuscript, please ensure that your figures adhere fully to these guidelines and provide the original underlying images for all blot or gel data reported in your submission. See the following link for instructions on providing the original image data: https://journals.plos.org/plosone/s/figures#loc-original-images-for-blots-and-gels.

In your cover letter, please note whether your blot/gel image data are in Supporting Information or posted at a public data repository, provide the repository URL if relevant, and provide specific

details as to which raw blot/gel images, if any, are not available. Email us at plosone@plos.org if you have any questions.

Author response: Thank you for your valuable comments. We have revised our submission to fully comply with PLOS ONE’s guidelines. The original uncropped and unadjusted images underlying all blot or gel results have been provided as per the journal’s requirements. In the cover letter we have mention the blot/gel image data are in Supporting Information.

Comments 3: Please include captions for your Supporting Information files at the end of your manuscript, and update any in-text citations to match accordingly. Please see our Supporting Information guidelines for more information: http://journals.plos.org/plosone/s/supporting-information.

Author response: Thank you for your valuable comments. We have revised our submission to fully comply with PLOS ONE’s guidelines. We have included captions for the Supporting Information files at the end of the manuscript and updated all in-text citations accordingly.

Comments 4: We notice that your supplementary figures are uploaded with the file type 'Other'. Please amend the file type to 'Supporting Information'. Please ensure that each Supporting Information file has a legend listed in the manuscript after the references list.

Author response: Thank you for your valuable comments. We have revised our submission to fully comply with PLOS ONE’s guidelines. We have updated the file type of the supplementary figures to 'Supporting Information' and ensured that each Supporting Information file has a corresponding legend listed in the manuscript after the references list.

Reviewer #1

Comments 1: The study included 20 rice varieties, but the criteria for their selection were not clearly defined. For example, whether these varieties are representative of all major cultivar types and whether they contain sufficient genetic diversity are not addressed. Without a comprehensive and representative sample, the generalizability of the study results may be compromised.

Author response: Thank you for your valuable comment. We selected 20 diverse indica rice varieties to ensure genetic variation and broad representation. These included popular mega varieties of Bangladesh where nine inbred varieties developed by the Bangladesh Institute of Nuclear Agriculture (BINA), seven varieties from the Bangladesh Rice Research Institute (BRRI) (including two hybrid and five inbred), three locally cultivated inbred varieties from Bangladesh (including Kasalath, an ozone-tolerant variety), and one variety from the International Rice Research Institute (IRRI). Details are now provided in Table 1 and elaborated in Section 2.1 of the manuscript.

Comments 2: Although the experiment included four replications, whether the statistical analysis adequately accounted for random error and replication should be detailed.

Author response: Thank you for your comment. We used four replications to ensure data reliability. Our statistical analysis accounted for variability, and no significant random errors or differences among replications were observed. Section 2.5 of the manuscript now details our approach to addressing replication in the analysis

Comments 3: EDU is applied weekly by foliar spraying, but specific times and conditions of application (e.g., light, temperature) are not detailed. These factors may greatly influence the effectiveness of EDU.

Author response: Thank you for this insight. We have now included specific details regarding EDU application conditions, such as light and temperature, in Section 2.4 of the manuscript. We have also refined the research objectives for greater coherence and precision.

Comments 4: The experimental procedure is described in detail in the "Materials and Methods" section, but the order in which the results are presented in the "Results" section does not correspond to the experimental design, which may confuse the reader.

Author response: Thank you for your suggestion. We have revised the "Materials and Methods" section for greater clarity and adjusted the "Results" section to enhance logical flow and coherence while maintaining our focus on the relationship between OsORAP1 expression and yield-related data.

Comments 5: Although the study mentions using R software for statistical analysis, the specific methods (e.g., whether multiple comparison corrections were applied) are not detailed. Moreover, for significant results (e.g., p-values), the biological implications are not further explained.

Author response: Thank you for your valuable comment. Section 2.5 now provides details on our statistical methods, including multiple comparison corrections. We have also expanded the discussion on the biological implications of significant results, particularly regarding EDU effects.

Comments 6: The study does not deeply explore the potential reasons for the differential responses to EDU among varieties. For example, why Kasalath-type varieties show less significant responses to EDU compared to Nipponbare-type varieties is not discussed. Are these differences related to genetic background or environmental adaptability?

Author response: Thank you for your insightful comment. We have examined differential responses by analyzing OsORAP1 expression. Previous studies (Ashrafuzzaman et al., 2017, 2018) suggest that tolerant genotypes exhibit minimal response to EDU compared to sensitive ones. This variation is likely due to genetic background, which may influence EDU's effectiveness in mitigating ozone stress.

Comments 7: The figures in the study could be further optimized. For example, some data labels in the figures are unclear, or sufficient legends are not provided.

Author response: Thank you for your observation. We have revised the figures to improve clarity, including better data labeling and more comprehensive legends for enhanced readability.

Comments 8: When presenting experimental results, some data lack complete statistical information. For example, for certain parameters (e.g., chlorophyll indices), only the mean values are provided without standard deviations or confidence intervals.

Author response: We have reviewed and updated statistical details in Section 2.2, incorporating standard deviations or confidence intervals where necessary. Figure labels have also been improved for better clarity.

Reviewer #2

Comments 1: In line 88-90, authors have mentioned "absence of *OsORAP1*…". Does this mean OsRAP1 gene does not exist in tolerant genotype?

Author response: Thank you for your comment. The phrase "absence of OsORAP1" refers to its low or no expression in tolerant genotypes, not the gene's physical absence. We have revised the text for clarity.

Comments 2: In line 95 – 97, higher induction of *OsORAP1* should lead to susceptibility not tolerance.

Author response: Thank you for pointing this out. We have revised the manuscript to accurately convey this concept.

Comments 3: Please provide methodology used for deriving various indices (NDVI, PRI, SIPI etc.)

Author response: Thank you for your valuable comment. We have provided the methodology for deriving various indices (NDVI, PRI, SIPI, etc.) in the "Materials and Methods" section, specifically in Section 2.2 ("Growing of Varieties and Design of the Experiments").

Comments 4: In line 168-170, authors have mentioned "Twenty genotypes (genotypes with Kasalath-type OsRAP1 allele)". However, all the 20 genotypes were not kasalath-type. This has to be rewritten.

Author response: We apologize for the oversight. The sentence has been revised to: "Among the twenty genotypes, selection was based on the presence or absence of a PCR band for further analyses."

Comments 5: In line no 170-173, results of PCR have been described, which may be shifted to results section.

Author response: Thank you for your valuable comment. Since our study primarily focuses on the expression of *OsORAP1*, we included the PCR band results in the "Materials and Methods" section to provide necessary context for our experimental approach. However, we have reconsidered the placement to ensure clarity and coherence in the manuscript.

Comments 6: Was the primer KAS_1_2 designed in this study or was it sources from literature. If it is designed by the authors, the methodology and the basis has to be elaborated.

Author response: The primer KAS_1_2 was sourced from Ashrafuzzaman et al., 2020. This clarification has been added to the manuscript.

Comments 7: The KAS_1_2 primer validation should be undertaken between Kasalath and Nipponbare. This is essential step for establishing genotypes as Kasalath-type and Nipponbare-type. However, Nipponbare is missing in the study.

Author response: Thank you for your suggestion. Prior studies (Ashrafuzzaman et al., 2020) confirm KAS_1_2 amplifies only in Kasalath-type genotypes, while Nipponbare-type primers produce bands exclusively in Nipponbare-type genotypes. We have clarified this in the manuscript.

Comments 8: Methodology used for scoring leaf bronzing, yield traits and reflectance indices should be included in the materials and methods section.

Author response: Thank you for your valuable comment. We appreciate your suggestion and have now included the methodology for scoring leaf bronzing, yield traits, and reflectance indices in the "Materials and Methods" section of the manuscript.

Comments 9: In line 118 of materials and methods section, it has been mentioned that 20 genotypes were evaluated in field. However, ANOVA for 10 selected genotypes has been presented.

Author response: Thank you for your valuable comment. Out of the 20 genotypes evaluated, we selected 10 for further analysis (5 Kasalath-type and 5 Nipponbare-type). Therefore, the ANOVA was conducted based on these 10 selected genotypes, which is why the results are presented accordingly.

Comments 10: Please shift the content of line no 248-249 to respective discussion section.

Author response: Thank you for your valuable comment. We have moved the content from lines 248-249 to the respective discussion section as suggested.

Comments 11: In fig 3, Kasalath is negatively responding to EDU treatment for leaf bronzing. However, authors have mentioned that Kasalath is not responsive to EDU treatment. This needs to be clarified.

Author response: Thank you for your valuable comment. We acknowledge the discrepancy in Figure 3, where Kasalath appears to negatively respond to EDU treatment for leaf bronzing. This response may be due to environmental factors or other variables influencing the outcome. We have clarified this in the manuscript to address the observed effect.

Comments 12: In Nipponbare-type varieties, BRRI hybrid dhan3 and BRRI dhan29 exhibited significant increase in grain yield/ plant and straw yield/ plant in response to EDU treatment, while other varieties did not show significant changes in either parameter. This needs to be discussed.

Author response: Thank you for your valuable comment. In Nipponbare-type varieties, BRRI hybrid dhan3 and BRRI dhan29 showed a significant increase in grain yield per plant and straw yield per plant in response to EDU treatment, while other varieties did not exhibit statistically significant changes. However, other Nipponbare-type varieties still demonstrated higher grain and straw yields, which may be influenced by genetic or environmental factors. We have included this discussion in the manuscript to provide further clarification.

Comments 13: Among the Kasalath-type allele carrying varieties, EDU treatment in one of the variety (BR11) has increased the grain and straw yield, while none of the remaining varieties have responded. This needs to be discussed.

Author response: Thank you for your valuable comment. Among the Kasalath-type allele-carrying varieties, BR11 exhibited an increase in grain and straw yield following EDU treatment, whereas the other varieties did not respond. This variation may be attributed to genetic or environmental factors. We have already addressed this point in the manuscript’s discussion section.

Comments 14: Association between the reflectance indices with yield traits should be estimated.

Author response: Thank you for your valuable comment. We have analyzed the association between reflectance indices and yield traits for both Kasalath-type and Nipponbare-type

varieties, with and without EDU treatment, and also have incorporated this information into the manuscript.

Comments 15: Line No. 327-329, authors have mentioned that "EDU treatment and its interaction with varieties did not yield significant responses across any of the studied reflectance indices parameters. Nonetheless, significant differences were observed among the varieties themselves for all parameters, suggesting distinct genetic responses irrespective of the EDU treatment". This indicates that there exist inherent differences among the genotypes for the tested traits. This does not imply the variation in genotypes is due to EDU treatment or due to their interaction.

Author response: Thank you for your valuable comment. We acknowledge the clarification. Our statement aimed to highlight that while EDU treatment and its interaction with Kasalath-type and Nipponbare-type varieties where it did not show significant effects on reflectance indices, the inherent genetic differences among the varieties were evident. We agree that this variation is independent of EDU treatment.

Comments 16: In Table 3, among Kasalath-type genotypes, Binadhan-17, Hutra and Kasalath the yield and related parameters have reduced upon EDU treatment over control. Similar situation exists for Nipponbare-type genotypes. This is contradictory. Needs thorough discussion.

Author response: Thank you for your valuable comment. In Table 3, the reduction in yield and related parameters upon EDU treatment in Kasalath-type genotypes (Binadhan-17, Hutra, and Kasalath), as well as in some Nipponbare-type genotypes, may indicate that EDU has little to no protective effect on ozone-tolerant genotypes. Additionally, the observed variations could be

influenced by environmental conditions or other genetic factors. We further elaborated on this in the discussion section to provide a clearer interpretation.

Comments 17: The *OsORAP1* gene expression level is reduced by EDU treatment. However, establishing its association with the yield and other related parameters is essential.

Author response: Thank you for your valuable comment. Throughout our study, we have aimed to establish the association between *OsORAP1* gene expression, yield, and other related parameters. We have further clarified this relationship in the discussion section to enhance the interpretation of our findings.

Comments 18: The treatment × variety interaction is non-significant for majority of the parameters under study. This indicates that the expression levels of OsRAP1 have no association with any of the tested parameters.

Author response: Thank you for your valuable comment. While the treatment × variety interaction is non-significant for some parameters, it is significant for key parameters such as *OsORAP1* expression and grain yield per plant in both Kasalath-type and Nipponbare-type varieties, which form the foundation of our study. We have already ensured these in the manuscript.

Comments 19: In line 335-337, authors have mentioned that "These findings suggest that EDU application can effectively mitigate ambient ozone stress in Nipponbare-type varieties, with different reflectance indices indicating its positive effects on leaf greenness, and photosynthesis content". The statement is contradicting as the variation for reflectance indices under treatment and interaction component is non-significant.

Author response: We appreciate the reviewer's critical observation. We acknowledge that the statement in lines 335–337 may not have been clearly articulated. While the interaction effect and treatment variation for some reflectance indices were non-significant, the overall trends observed in leaf greenness and photosynthetic parameters indicate a positive influence of EDU application. To clarify this, we have revised the statement as follows:

"These findings suggest that EDU application can provide some degree of protection against ambient ozone stress in Nipponbare-type varieties. Although the variation for certain reflectance indices under treatment and interaction effects was not statistically significant, trends in leaf greenness and photosynthesis-related parameters indicate a positive influence of EDU."

This revision ensures consistency with the statistical results while maintaining the interpretation of EDU's protective effects. Thank you for pointing this out.

Comments 20: Although, the ANOVA indicates no significant variation for treatment and interaction components for most of the parameters tested. However, authors indicate that there exists influence of treatment and its interaction with genotype although the text. Which is contradictory.

Author response: Thank you for your valuable comment. We acknowledge that ANOVA indicates no significant variation for treatment and interaction components for most of the parameters tested. However, within the varieties, while some parameters showed non-significant effects, key parameters such as *OsORAP1* expression and grain yield per plant exhibited significant differences in both Kasalath-type and Nipponbare-type varieties. These significant findings form the foundation of our study and support our conclusions regarding the impact of EDU treatment under ozone stress. Thank you for your insightful feedback.

Comments 21: Line no 478 and 479 – Authors have mentioned that Nipponbare-type varieties have increased symptom formation and expression under EDU treatment. This contradicts the hypothesis of the paper.

Author response: Thank you for your valuable comment. We acknowledge that the statement in lines 478–479 may appear contradictory to our hypothesis. However, our findings indicate that Nipponbare-type varieties displayed significant increases in *OsORAP1* expression and visible symptom formation (leaf bronzing score) in response to EDU treatment, while Kasalath-type varieties showed less significant responses due to their inherent tolerance to ozone stress. This suggests that EDU's protective effects may vary depending on the genetic background of the variety. To avoid confusion, we have revised the text for clarity and ensured that our interpretation aligns with the study's hypothesis. We appreciate your feedback in helping us improve the manuscript.
