# Supplementary material for: Ethylenediurea (EDU) inhibits OsORAP1 expression in rice (Oryza sativa L:): Varietal differences in ozone protection efficacy
Source: PLoS One. 2025 Jul 2;20(7):e0327162. doi: 10.1371/journal.pone.0327162 (PMC12220992; doi:10.1371/journal.pone.0327162)
Supplement: Attachment — Submitted filename: Response to Reviewers Comments.docx [file pone.0327162.s010.docx]

**Academic editor and Reviewer response**

**Academic editor**

**Comments 1:**  Please ensure that your manuscript meets PLOS ONE's style requirements, including those for file naming. The PLOS ONE style templates can be found at

https://journals.plos.org/plosone/s/file?id=wjVg/PLOSOne_formatting_sample_main_body.pdf and

https://journals.plos.org/plosone/s/file?id=ba62/PLOSOne_formatting_sample_title_authors_affiliations.pdf

*Author response:* Thank you for your valuable comments. We have revised the manuscript to ensure it fully complies with PLOS ONE's style requirements, including formatting and file naming, as per the provided guidelines.

**Comments 2:**  PLOS ONE now requires that authors provide the original uncropped and unadjusted images underlying all blot or gel results reported in a submission’s figures or Supporting Information files. This policy and the journal’s other requirements for blot/gel reporting and figure preparation are described in detail at https://journals.plos.org/plosone/s/figures#loc-blot-and-gel-reporting-requirements and https://journals.plos.org/plosone/s/figures#loc-preparing-figures-from-image-files. When you submit your revised manuscript, please ensure that your figures adhere fully to these guidelines and provide the original underlying images for all blot or gel data reported in your submission. See the following link for instructions on providing the original image data: <https://journals.plos.org/plosone/s/figures#loc-original-images-for-blots-and-gels>.

In your cover letter, please note whether your blot/gel image data are in Supporting Information or posted at a public data repository, provide the repository URL if relevant, and provide specific details as to which raw blot/gel images, if any, are not available. Email us at plosone@plos.org if you have any questions.

*Author response:* Thank you for your valuable comments. We have revised our submission to fully comply with PLOS ONE’s guidelines. The original uncropped and unadjusted images underlying all blot or gel results have been provided as per the journal’s requirements. In the cover letter we have mention the blot/gel image data are in Supporting Information.

**Comments 3:**  Please include captions for your Supporting Information files at the end of your manuscript, and update any in-text citations to match accordingly. Please see our Supporting Information guidelines for more information: <http://journals.plos.org/plosone/s/supporting-information>.

*Author response:* Thank you for your valuable comments. We have revised our submission to fully comply with PLOS ONE’s guidelines. We have included captions for the Supporting Information files at the end of the manuscript and updated all in-text citations accordingly.

**Comments 4:**  We notice that your supplementary figures are uploaded with the file type 'Other'. Please amend the file type to 'Supporting Information'. Please ensure that each Supporting Information file has a legend listed in the manuscript after the references list.

*Author response:* Thank you for your valuable comments. We have revised our submission to fully comply with PLOS ONE’s guidelines. We have updated the file type of the supplementary figures to 'Supporting Information' and ensured that each Supporting Information file has a corresponding legend listed in the manuscript after the references list.

**Reviewer #1**

**Comments 1:**  The study included 20 rice varieties, but the criteria for their selection were not clearly defined. For example, whether these varieties are representative of all major cultivar types and whether they contain sufficient genetic diversity are not addressed. Without a comprehensive and representative sample, the generalizability of the study results may be compromised.

***Author response:*** Thank you for your valuable comment. We selected 20 diverse indica rice varieties to ensure genetic variation and broad representation. These included popular mega varieties of Bangladesh where nine inbred varieties developed by the Bangladesh Institute of Nuclear Agriculture (BINA), seven varieties from the Bangladesh Rice Research Institute (BRRI) (including two hybrid and five inbred), three locally cultivated inbred varieties from Bangladesh (including Kasalath, an ozone-tolerant variety), and one variety from the International Rice Research Institute (IRRI). Details are now provided in Table 1 and elaborated in Section 2.1 of the manuscript.

**Comments 2:** Although the experiment included four replications, whether the statistical analysis adequately accounted for random error and replication should be detailed.

***Author response:*** Thank you for your comment. We used four replications to ensure data reliability. Our statistical analysis accounted for variability, and no significant random errors or differences among replications were observed. Section 2.5 of the manuscript now details our approach to addressing replication in the analysis

**Comments 3:** EDU is applied weekly by foliar spraying, but specific times and conditions of application (e.g., light, temperature) are not detailed. These factors may greatly influence the effectiveness of EDU.

***Author response:*** Thank you for this insight. We have now included specific details regarding EDU application conditions, such as light and temperature, in Section 2.4 of the manuscript. We have also refined the research objectives for greater coherence and precision.

**Comments 4:** The experimental procedure is described in detail in the “Materials and Methods” section, but the order in which the results are presented in the “Results” section does not correspond to the experimental design, which may confuse the reader.

***Author response:*** Thank you for your suggestion. We have revised the "Materials and Methods" section for greater clarity and adjusted the "Results" section to enhance logical flow and coherence while maintaining our focus on the relationship between OsORAP1 expression and yield-related data.

**Comments 5:** Although the study mentions using R software for statistical analysis, the specific methods (e.g., whether multiple comparison corrections were applied) are not detailed. Moreover, for significant results (e.g., p-values), the biological implications are not further explained.

***Author response:*** Thank you for your valuable comment. Section 2.5 now provides details on our statistical methods, including multiple comparison corrections. We have also expanded the discussion on the biological implications of significant results, particularly regarding EDU effects.

**Comments 6:** The study does not deeply explore the potential reasons for the differential responses to EDU among varieties. For example, why Kasalath-type varieties show less significant responses to EDU compared to Nipponbare-type varieties is not discussed. Are these differences related to genetic background or environmental adaptability?

*Author response:* Thank you for your insightful comment. We have examined differential responses by analyzing OsORAP1 expression. Previous studies (Ashrafuzzaman et al., 2017, 2018) suggest that tolerant genotypes exhibit minimal response to EDU compared to sensitive ones. This variation is likely due to genetic background, which may influence EDU’s effectiveness in mitigating ozone stress.

**Comments 7:** The figures in the study could be further optimized. For example, some data labels in the figures are unclear, or sufficient legends are not provided.

***Author response:*** Thank you for your observation. We have revised the figures to improve clarity, including better data labeling and more comprehensive legends for enhanced readability.

**Comments 8:** When presenting experimental results, some data lack complete statistical information. For example, for certain parameters (e.g., chlorophyll indices), only the mean values are provided without standard deviations or confidence intervals.

***Author response****:* We have reviewed and updated statistical details in Section 2.2, incorporating standard deviations or confidence intervals where necessary. Figure labels have also been improved for better clarity.

**Reviewer #2**

**Comments 1:** In line 88-90, authors have mentioned “absence of *OsORAP1*…”. Does this mean OsRAP1 gene does not exist in tolerant genotype?

***Author response:*** Thank you for your comment. The phrase "absence of OsORAP1" refers to its low or no expression in tolerant genotypes, not the gene’s physical absence. We have revised the text for clarity.

**Comments 2:** In line 95 – 97, higher induction of *OsORAP1* should lead to susceptibility not tolerance.

***Author response:*** Thank you for pointing this out. We have revised the manuscript to accurately convey this concept.

**Comments 3:** Please provide methodology used for deriving various indices (NDVI, PRI, SIPI etc.)

***Author response:*** Thank you for your valuable comment. We have provided the methodology for deriving various indices (NDVI, PRI, SIPI, etc.) in the "Materials and Methods" section, specifically in Section 2.2 ("Growing of Varieties and Design of the Experiments").

**Comments 4:** In line 168-170, authors have mentioned “Twenty genotypes (genotypes with Kasalath-type OsRAP1 allele)”. However, all the 20 genotypes were not kasalath-type. This has to be rewritten.

***Author response****:* We apologize for the oversight. The sentence has been revised to: “Among the twenty genotypes, selection was based on the presence or absence of a PCR band for further analyses.”

**Comments 5:** In line no 170-173, results of PCR have been described, which may be shifted to results section.

*Author response:* Thank you for your valuable comment. Since our study primarily focuses on the expression of *OsORAP1*, we included the PCR band results in the "Materials and Methods" section to provide necessary context for our experimental approach. However, we have reconsidered the placement to ensure clarity and coherence in the manuscript.

**Comments 6:** Was the primer KAS_1_2 designed in this study or was it sources from literature. If it is designed by the authors, the methodology and the basis has to be elaborated.

***Author response:*** The primer KAS_1_2 was sourced from Ashrafuzzaman et al., 2020. This clarification has been added to the manuscript.

**Comments 7:** The KAS_1_2 primer validation should be undertaken between Kasalath and Nipponbare. This is essential step for establishing genotypes as Kasalath-type and Nipponbare-type. However, Nipponbare is missing in the study.

*Author response:* Thank you for your suggestion. Prior studies (Ashrafuzzaman et al., 2020) confirm KAS_1_2 amplifies only in Kasalath-type genotypes, while Nipponbare-type primers produce bands exclusively in Nipponbare-type genotypes. We have clarified this in the manuscript.

**Comments 8:** Methodology used for scoring leaf bronzing, yield traits and reflectance indices should be included in the materials and methods section.

***Author response:*** Thank you for your valuable comment. We appreciate your suggestion and have now included the methodology for scoring leaf bronzing, yield traits, and reflectance indices in the "Materials and Methods" section of the manuscript.

**Comments 9:** In line 118 of materials and methods section, it has been mentioned that 20 genotypes were evaluated in field. However, ANOVA for 10 selected genotypes has been presented.

***Author response:*** Thank you for your valuable comment. Out of the 20 genotypes evaluated, we selected 10 for further analysis (5 Kasalath-type and 5 Nipponbare-type). Therefore, the ANOVA was conducted based on these 10 selected genotypes, which is why the results are presented accordingly.

**Comments 10:** Please shift the content of line no 248-249 to respective discussion section.

***Author response:*** Thank you for your valuable comment. We have moved the content from lines 248-249 to the respective discussion section as suggested.

**Comments 11:** In fig 3, Kasalath is negatively responding to EDU treatment for leaf bronzing. However, authors have mentioned that Kasalath is not responsive to EDU treatment. This needs to be clarified.

***Author response:*** Thank you for your valuable comment. We acknowledge the discrepancy in Figure 3, where Kasalath appears to negatively respond to EDU treatment for leaf bronzing. This response may be due to environmental factors or other variables influencing the outcome. We have clarified this in the manuscript to address the observed effect.

**Comments 12:** In Nipponbare-type varieties, BRRI hybrid dhan3 and BRRI dhan29 exhibited significant increase in grain yield/ plant and straw yield/ plant in response to EDU treatment, while other varieties did not show significant changes in either parameter. This needs to be discussed.

***Author response:*** Thank you for your valuable comment. In Nipponbare-type varieties, BRRI hybrid dhan3 and BRRI dhan29 showed a significant increase in grain yield per plant and straw yield per plant in response to EDU treatment, while other varieties did not exhibit statistically significant changes. However, other Nipponbare-type varieties still demonstrated higher grain and straw yields, which may be influenced by genetic or environmental factors. We have included this discussion in the manuscript to provide further clarification.

**Comments 13:** Among the Kasalath-type allele carrying varieties, EDU treatment in one of the variety (BR11) has increased the grain and straw yield, while none of the remaining varieties have responded. This needs to be discussed.

***Author response:*** Thank you for your valuable comment. Among the Kasalath-type allele-carrying varieties, BR11 exhibited an increase in grain and straw yield following EDU treatment, whereas the other varieties did not respond. This variation may be attributed to genetic or environmental factors. We have already addressed this point in the manuscript’s discussion section.

**Comments 14:** Association between the reflectance indices with yield traits should be estimated.

***Author response:*** Thank you for your valuable comment. We have analyzed the association between reflectance indices and yield traits for both Kasalath-type and Nipponbare-type varieties, with and without EDU treatment, and also have incorporated this information into the manuscript.

**Comments 15:** Line No. 327-329, authors have mentioned that “EDU treatment and its interaction with varieties did not yield significant responses across any of the studied reflectance indices parameters. Nonetheless, significant differences were observed among the varieties themselves for all parameters, suggesting distinct genetic responses irrespective of the EDU treatment”. This indicates that there exist inherent differences among the genotypes for the tested traits. This does not imply the variation in genotypes is due to EDU treatment or due to their interaction.

***Author response:*** Thank you for your valuable comment. We acknowledge the clarification. Our statement aimed to highlight that while EDU treatment and its interaction with Kasalath-type and Nipponbare-type varieties where it did not show significant effects on reflectance indices, the inherent genetic differences among the varieties were evident. We agree that this variation is independent of EDU treatment.

**Comments 16:** In Table 3, among Kasalath-type genotypes, Binadhan-17, Hutra and Kasalath the yield and related parameters have reduced upon EDU treatment over control. Similar situation exists for Nipponbare-type genotypes. This is contradictory. Needs thorough discussion.

***Author response:*** Thank you for your valuable comment. In Table 3, the reduction in yield and related parameters upon EDU treatment in Kasalath-type genotypes (Binadhan-17, Hutra, and Kasalath), as well as in some Nipponbare-type genotypes, may indicate that EDU has little to no protective effect on ozone-tolerant genotypes. Additionally, the observed variations could be influenced by environmental conditions or other genetic factors. We further elaborated on this in the discussion section to provide a clearer interpretation.

**Comments 17:** The *OsORAP1* gene expression level is reduced by EDU treatment. However, establishing its association with the yield and other related parameters is essential.

***Author response:*** Thank you for your valuable comment. Throughout our study, we have aimed to establish the association between *OsORAP1* gene expression, yield, and other related parameters. We have further clarified this relationship in the discussion section to enhance the interpretation of our findings.

**Comments 18:** The treatment × variety interaction is non-significant for majority of the parameters under study. This indicates that the expression levels of OsRAP1 have no association with any of the tested parameters.

***Author response****:* Thank you for your valuable comment. While the treatment × variety interaction is non-significant for some parameters, it is significant for key parameters such as *OsORAP1* expression and grain yield per plant in both Kasalath-type and Nipponbare-type varieties, which form the foundation of our study. We have already ensured these in the manuscript.

**Comments 19:** In line 335-337, authors have mentioned that “These findings suggest that EDU application can effectively mitigate ambient ozone stress in Nipponbare-type varieties, with different reflectance indices indicating its positive effects on leaf greenness, and photosynthesis content”. The statement is contradicting as the variation for reflectance indices under treatment and interaction component is non-significant.

***Author response:*** We appreciate the reviewer's critical observation. We acknowledge that the statement in lines 335–337 may not have been clearly articulated. While the interaction effect and treatment variation for some reflectance indices were non-significant, the overall trends observed in leaf greenness and photosynthetic parameters indicate a positive influence of EDU application. To clarify this, we have revised the statement as follows:

"These findings suggest that EDU application can provide some degree of protection against ambient ozone stress in Nipponbare-type varieties. Although the variation for certain reflectance indices under treatment and interaction effects was not statistically significant, trends in leaf greenness and photosynthesis-related parameters indicate a positive influence of EDU."

This revision ensures consistency with the statistical results while maintaining the interpretation of EDU’s protective effects. Thank you for pointing this out.

**Comments 20:** Although, the ANOVA indicates no significant variation for treatment and interaction components for most of the parameters tested. However, authors indicate that there exists influence of treatment and its interaction with genotype although the text. Which is contradictory.

***Author response:*** Thank you for your valuable comment. We acknowledge that ANOVA indicates no significant variation for treatment and interaction components for most of the parameters tested. However, within the varieties, while some parameters showed non-significant effects, key parameters such as *OsORAP1* expression and grain yield per plant exhibited significant differences in both Kasalath-type and Nipponbare-type varieties. These significant findings form the foundation of our study and support our conclusions regarding the impact of EDU treatment under ozone stress. Thank you for your insightful feedback.

**Comments 21:** Line no 478 and 479 – Authors have mentioned that Nipponbare-type varieties have increased symptom formation and expression under EDU treatment. This contradicts the hypothesis of the paper.

***Author response:*** Thank you for your valuable comment. We acknowledge that the statement in lines 478–479 may appear contradictory to our hypothesis. However, our findings indicate that Nipponbare-type varieties displayed significant increases in *OsORAP1* expression and visible symptom formation (leaf bronzing score) in response to EDU treatment, while Kasalath-type varieties showed less significant responses due to their inherent tolerance to ozone stress. This suggests that EDU’s protective effects may vary depending on the genetic background of the variety. To avoid confusion, we have revised the text for clarity and ensured that our interpretation aligns with the study’s hypothesis. We appreciate your feedback in helping us improve the manuscript.
