# Supplementary material for: Ethylenediurea (EDU) inhibits OsORAP1 expression in rice (Oryza sativa L:): Varietal differences in ozone protection efficacy
Source: PLoS One. 2025 Jul 2;20(7):e0327162. doi: 10.1371/journal.pone.0327162 (PMC12220992; doi:10.1371/journal.pone.0327162)
Supplement: Attachment — Submitted filename: Response to Reviewers.docx [file pone.0327162.s011.docx]

**Academic editor and Reviewer response**

**Academic editor**

**Comments 1:**  Journal Requirements:

Please review your reference list to ensure that it is complete and correct. If you have cited papers that have been retracted, please include the rationale for doing so in the manuscript text, or remove these references and replace them with relevant current references. Any changes to the reference list should be mentioned in the rebuttal letter that accompanies your revised manuscript. If you need to cite a retracted article, indicate the article’s retracted status in the References list and also include a citation and full reference for the retraction notice.

***Author response:*** Thank you for your comment regarding the reference list. We have carefully reviewed all cited references in the manuscript to ensure completeness and accuracy.

- We confirm that none of the references cited in the revised manuscript have been retracted.
- Minor corrections have been made to formatting inconsistencies and citation details (e.g., author names, journal names, volume/page numbers) to ensure compliance with journal guidelines.
- A few outdated references have been replaced with more recent and relevant literature to strengthen the manuscript's background and discussion sections.

All changes to the reference list have been clearly marked in the revised manuscript, and these updates are detailed in the point-by-point responses where applicable.

Thank you for your guidance.

**Reviewers' comments:**

**Reviewer #1**

**Comment 1:** I recommend that the authors consider revising the title of the manuscript to enhance its appeal. A potential title could be “Ethylenediurea inhibits the expression of OsORAP1 in rice, but the protections against ambient ozone vary between varieties”

***Author response:*** We appreciate the reviewer’s thoughtful suggestion regarding the manuscript title. After careful consideration, we have revised the title to better reflect the key findings and improve the manuscript's appeal. Please see below the new title which we amended in the manuscript:

“Ethylenediurea (EDU) inhibits OsORAP1 expression in rice (Oryza sativa L.): varietal differences in ozone protection efficacy”

This revised title captures both the molecular and varietal aspects of the study and aligns well with the reviewer's recommendation.

**Comments 2:** Page 2, line 19, it is suggested that the authors include a brief introduction about OsORAP1 and EDU to provide context for readers.

***Author response:*** We thank the reviewer for this valuable suggestion. In response, we have revised the sentence on Page 2, line 19 to include a brief introduction to both OsORAP1 and EDU. The revised sentence now reads:

“This study investigates the response of the OsORAP1 allele—an important regulator of the plant's response to ozone stress, whose expression is associated with ozone-induced damage—in different rice varieties, particularly Kasalath-type and Nipponbare-type, under treatment with ethylenediurea (EDU), a protective antiozonant used in plant research under ambient ozone stress.”

This revision provides the necessary background to help readers better understand the context of our study.

**Comments 3:** Page 2, line 27, the phrase “more leaf bronzing score (LBS) following EDU treatment” could potentially mislead readers regarding EDU’s protective effect against ambient ozone. It is recommended to revise it to “more leaf bronzing score (LBS) without EDU protection”.

***Author response:*** We thank the reviewer for pointing out the potential for misinterpretation. To accurately reflect EDU's protective role, we have revised the phrase on Page 2, line 27 to read:

“more leaf bronzing score (LBS) without EDU protection.”

This correction ensures clarity and better aligns with the intended meaning regarding the effects of ozone stress and the role of EDU.

**Comments 4:** Page 28, line 519, to enhance clarity, it is advisable to replace “EDU treatment” with another expression.

***Author response:*** Thank you for your helpful comment. We have revised the sentence to enhance clarity. It now reads:

“Nipponbare-type varieties displayed significant increases in *OsORAP1* expression and leaf bronzing score in control plants than the EDU-treated plants, while Kasalath-type varieties showed less significant responses due to their inherent tolerance to ozone stress.”

This revision clarifies the comparison between control and EDU-treated plants and aligns better with the intended meaning.

**Comments 5:** For the conclusion, the author should properly highlight the potential impact of their research. For instance, they could mention adapting rice to higher ozone conditions by screening and cultivating tolerant cultivars, and by optimizing the use of EDU and other protectants. This would emphasize the practical implications and significance of their work.

***Author response:*** We thank the reviewer for this insightful suggestion. In response, we have revised the conclusion to better highlight the practical implications of our findings. Specifically, we now emphasize the potential for adapting rice to increasing ambient ozone levels through the development and cultivation of ozone-tolerant varieties. We also discuss the role of EDU and other protectants as mitigation tools for ozone stress in sensitive varieties, while noting their limitations in terms of availability and use in agricultural practice. These additions underscore the broader significance of our research for crop improvement and the sustainability of rice production under changing environmental conditions.

**Reviewer #3**

**Comments:** Abstract: Line 22-23: What level of ppm of EDU was used in this experiment?

***Author response:*** Thank you for pointing this out. We have now clarified the concentration of EDU used in the experiment in the Abstract section. Specifically, we used 300 ppm of EDU, applied as a foliar spray at regular intervals. The revised sentence now reads:

"The experiment, conducted during the 2022 rainfed season, involved growing of 20 rice varieties under a split-plot design with and without 300 ppm EDU treatment, followed by genomic DNA collection at the vegetative stage to differentiate Kasalath-types and Nipponbare-types, and RNA extraction from 10 selected varieties at the flowering stage for gene expression analysis."

This correction has been incorporated into the revised manuscript.

**Comments:** Introduction: Add literature review (add 4-5 references from similar previous studies) regarding EDU application in rice. Provide limitation of this study.

***Author response:*** We thank the reviewer for this valuable suggestion. In the revised manuscript, we have expanded the Introduction section to include a brief literature review on the use of ethylenediurea (EDU) in rice studies. We have incorporated references to relevant previous research that demonstrates the protective role of EDU against ozone-induced stress in rice. The revised section now includes the following studies:

Previous studies have explored the use of ethylenediurea (EDU) as a protective agent against ozone stress in rice. Several researchers have reported that EDU can enhance rice yield, primarily through improvements in grain weight rather than increases in the number of panicles or grains per plant (Shang et al., 2022). Under conditions of elevated surface ozone, EDU has been shown to provide moderate mitigation of yield loss, suggesting its potential as a practical tool for safeguarding rice productivity (Zhang et al., 2022; Ashrafuzzaman et al., 2018). Interestingly, its effectiveness appears to vary between rice genotypes, with some evidence indicating that inbred cultivars benefit more from EDU treatment than hybrid varieties (Zhang et al., 2010). In addition to its physiological effects, EDU application does not appear to significantly alter gene expression patterns, highlighting its suitability for field-based ozone screening without interfering with molecular responses. More recently, Frei et al. (2024) demonstrated that EDU can serve as a diagnostic tool to differentiate between ozone-sensitive and ozone-tolerant rice genotypes, with yield increases observed up to 21% in responsive varieties. These findings collectively underscore the potential of EDU in both protecting rice from ozone stress and facilitating the identification of tolerant cultivars. These studies provide a strong background for our work and highlight the rationale for using EDU in our experiment.

In addition, we have now clearly stated the limitation of the present study. While the study identifies ozone-tolerant rice varieties and explores their genetic expression profiles, it is limited to a single growing season and location. Furthermore, the effect of EDU was studied at only one concentration (300 ppm), and the long-term agronomic and physiological impacts under varying field conditions were not assessed. These aspects should be explored in future multi-location and multi-season trials.

**Comments:** Line 110-111, Keep this highlighted text before aim of this experiment.

***Author response:*** Thank you for your suggestion. As per your recommendation, the sentence "The study was conducted based on 10 Bangladeshi rice varieties during their flowering stage with and without the application of EDU (300 ppm, once a week after transplanting until harvesting)" has been moved to appear before the statement outlining the aim of the experiment. This revision improves the logical flow of the methodology and clarifies the experimental context prior to presenting the research objective.

**Comments:** Materials and Methods: Provide equation number for formula used.

***Author response:*** Thank you for your valuable comment. We have added equation numbers to all the formulas used in the manuscript to enhance clarity and ease of reference.

**Comments:** Results: Table 2: What does these star sign indicate for what information. Write in footnote of this table.

***Author response:*** Thank you for pointing this out. We have now clarified the meaning of the star signs in Table 2. Specifically, the asterisks indicate the level of statistical significance, as follows:

*p < 0.05, **p < 0.01, ***p < 0.001.

We have added this information as a footnote to Table 2 in the revised manuscript to ensure clarity for readers.

**Comments:** Table 3. Provide SEm along with mean data

***Author response:*** Thank you for your valuable suggestion. We have now included the Standard Error of the Mean (SEm) alongside the mean values in Table 3 to provide a better understanding of the data variability. The revised table with SEm values is now incorporated in the updated manuscript.

**Comments:** Figure 1: Provide name of X-axis. Provide color line graph in Figure 1

***Author response:*** Thank you for your helpful suggestions. We have revised Figure 1 to address both points:

X-axis Label: We have added an appropriate label to the X-axis to clearly indicate the variable being represented.

Color Line Graph: We have converted the graph to a color format to enhance visual clarity and distinguishability of the data series.

The updated figure has been included in the revised manuscript (see revised Figure 1). We hope these improvements meet your expectations.

**Comments:** Figure 2, Figure 3, Figure 4: Provide color bar graph

***Author response:*** Thank you for your valuable feedback. We have revised Figures 2, 3, and 4 to present the bar graphs in color. This modification enhances the visual clarity and makes it easier to differentiate between data groups. The updated figures have been incorporated into the revised manuscript.

We appreciate your suggestion, which has helped improve the overall presentation of our data.

**Comments:** References: All references should follow the journal's rules.

***Author response:*** Thank you for pointing this out. We have carefully reviewed and revised all references to ensure they fully comply with the journal’s formatting guidelines. This includes adjustments to author names, journal titles, volume and issue numbers, page ranges, and DOI formatting where applicable. We appreciate your attention to detail in helping us improve the manuscript’s consistency.
